# Illumination of Hydroxyl Radical in Kidney Injury and High‐Throughput Screening of Natural Protectants Using a Fluorescent/Photoacoustic Probe

**DOI:** 10.1002/advs.202303926

**Published:** 2023-10-23

**Authors:** Han Gao, Lei Sun, Jiwei Li, Qilin Zhou, Haijun Xu, Xiao‐Nan Ma, Renshi Li, Bo‐Yang Yu, Jiangwei Tian

**Affiliations:** ^1^ State Key Laboratory of Natural Medicines Jiangsu Key Laboratory of TCM Evaluation and Translational Research Cellular and Molecular Biology Center School of Traditional Chinese Pharmacy China Pharmaceutical University Nanjing 211198 P. R. China; ^2^ Jiangsu Co‐innovation Center of Efficient Processing and Utilization of Forest Resources, Key Laboratory of Forestry Genetics & Biotechnology of Ministry of Education, Jiangsu Provincial Key Lab for the Chemistry and Utilization of Agroforest Biomass College of Chemical Engineering Nanjing Forestry University Nanjing 210037 P. R. China; ^3^ School of Chemistry and Chemical Engineering Henan Normal University Xinxiang 453002 P. R. China

**Keywords:** acute kidney injury, high‐throughput screening, hydroxyl radical, molecular probe, natural products

## Abstract

The hydroxyl radical (•OH) is shown to play a crucial role in the occurrence and progression of acute kidney injury (AKI). Therefore, the development of a robust •OH probe holds great promise for the early diagnosis of AKI, high‐throughput screening (HTS) of natural protectants, and elucidating the molecular mechanism of intervention in AKI. Herein, the design and synthesis of an activatable fluorescent/photoacoustic (PA) probe (CDIA) for sensitive and selective imaging of •OH in AKI is reported. CDIA has near‐infrared fluorescence/PA channels and fast activation kinetics, enabling the detection of the onset of •OH in an AKI model. The positive detection time of 12 h using this probe is superior to the 48‐hour detection time for typical clinical assays, such as blood urea nitrogen and serum creatinine detection. Furthermore, a method is established using CDIA for HTS of natural •OH inhibitors from herbal medicines. Puerarin is screened out by activating the Sirt1/Nrf2/Keap1 signaling pathway to protect renal cells in AKI. Overall, this work provides a versatile and dual‐mode tool for illuminating the •OH‐related pathological process in AKI and screening additional compounds to prevent and treat AKI.

## Graphical abstract

An activatable fluorescent/photoacoustic probe (CDIA) is developed for sensitive and selective imaging of hydroxyl radical •OH during the progression of acute kidney injury. A high‐throughput screening method using CDIA is established to identify natural •OH inhibitors from herbal medicines, and puerarin is screened out to protect renal cells by activating the Sirt1/Nrf2/Keap1 signaling pathway.

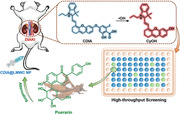

## 1 Introduction

Acute kidney injury (AKI) is a serious complication that affects hospitalized patients at a high risk of developing progressive chronic kidney disease or end‐stage renal disease. It is responsible for over 1.5 million deaths globally each year,^[^ ^1^ ^]^ making the timely diagnosis and treatment of AKI critical for preventing and managing kidney diseases. In vitro diagnostic methods have been employed to monitor renal function and prevent AKI. However, the current diagnostic approach for kidney disease in clinical settings relies on static analysis of blood urea nitrogen (BUN) and serum creatinine (sCr),^[^ ^2^ ^]^ which only increase after a 50% decrease in glomerular filtration rate (GFR).^[^ ^3^ ^]^ Non‐invasive imaging strategies, such as single‐photon emission computed tomography (SPECT) and magnetic resonance imaging (MRI), are limited by in vitro diagnostic methods based on static analysis, which are difficult for dynamic monitoring of renal functional impairment.^[^ ^4^ ^]^ Molecular optical imaging offers a noninvasive way to occurrence and development of different kinds of diseases in organisms in real time with high spatiotemporal resolution,^[^ ^5^, ^6^, ^7^ ^]^ making it a promising tool for in situ detection of molecular processes associated with renal disease.^[^ ^8^ ^]^ Many fluorescent probes have been reported for AKI imaging, but the shallow penetration depth of fluorescence limits their further application for in vivo imaging.^[^ ^9^ ^]^ In contrast, photoacoustic (PA) imaging, a hybrid imaging modality that detects sound rather than photons, has minimal attenuation due to negligible sound scattering in living organisms. This property offers a deeper penetration depth (7–10 cm) and higher spatial resolution for biological imaging.^[^ ^10^ ^]^ Activatable PA molecular probes have already been explored for deep‐tissue imaging,^[^ ^11^, ^12^ ^]^ and the deeper imaging in the kidney avoids biological background signal fluctuations, enabling earlier and more precise detection of renal dysfunction.

Therapeutic options for preventing AKI involve the use of small molecular agents like *N*‐acetyl cysteine (NAC), which help reduce oxidative damage.^[^ ^13^ ^]^ In recent studies, nanoreagents with reactive oxygen species (ROS)‐modulating capabilities have been explored as potential therapeutic strategies for ROS‐associated AKI.^[^ ^14^, ^15^ ^]^ DNA origami nanomaterials, for example, have demonstrated a strong ability to scavenge ROS, preserve kidney structures, and improve AKI condition.^[^ ^16^ ^]^ However, the sensitivity of nanomaterials to the biological environment, including subtle changes in shape, morphology, or physical properties, as well as the lack of practical guidelines, pose significant challenges in this field of research.^[^ ^17^ ^]^ Complementary and alternative medicine could potentially play a unique role in treating AKI. In China, traditional Chinese medicine (TCM) has been used for thousands of years to prevent diseases. Natural products, such as epigallocatechin‐*3*‐gallate,^[^ ^18^ ^]^ saikosaponin‐*D*,^[^ ^19^ ^]^ and resveratrol,^[^ ^20^ ^]^ have been employed to treat drug‐induced nephrotoxicity, which supports the exploration of effective natural products from medicinal plants for AKI treatment. Nevertheless, developing natural products into effective therapies for AKI presents challenges due to the complicated ingredients and multiple‐target nature of natural products, and testing their efficacy and safety in disease models can be a time‐consuming and costly process. Therefore, direct visualization of AKI in vivo and rapid screening of efficient natural products for AKI treatment are highly desirable but challenging goals.

AKI is a complex condition triggered by ischemic and inflammatory components that ultimately result in the loss of renal epithelial cells and tubular cell dysfunction. Among various pathological factors associated with AKI, oxidative stress (OS) has been identified as the most crucial.^[^ ^21^ ^]^ OS is caused by ROS such as superoxide anion, hydrogen peroxide, and hydroxyl radical (•OH). While ROS play a crucial role in maintaining physiological functions, their hyper‐regulated or unregulated accumulation can cause oxidative damage to biomolecules, perturb cell membrane, macromolecular, and organelle functions. Severe pathophysiological conditions can enhance ROS production, leading to vascular dysfunction, inflammation, and tubular cytotoxicity, commonly observed in the pathogenesis of AKI.^[^ ^22^ ^]^ Animal studies have shown that the occurrence of AKI increases oxidative damage in the body while decreasing tissue antioxidant status.^[^ ^23^, ^24^ ^]^ Although the exact mechanism of ROS generation in AKI remains unknown, ROS has been identified as an early hallmark of AKI,^[^ ^25^, ^26^ ^]^ especially •OH with the highest reduction potential (2.31 V) compared with other ROS.^[^ ^27^ ^]^ Therefore, it can be chosen as a target for intervention of AKI.

**Figure 1:**
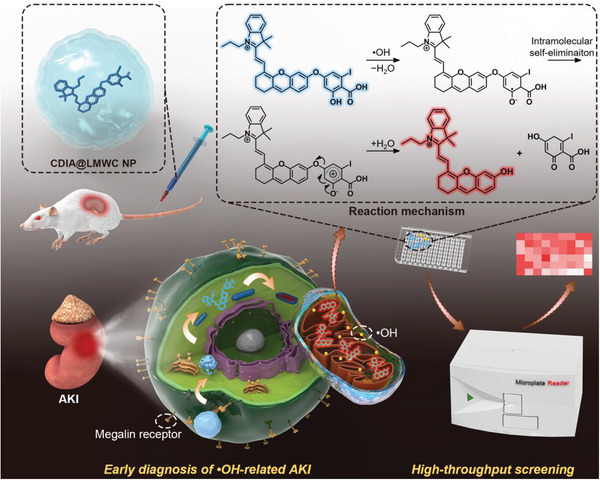
Schematic diagram to indicate the design of a NIR fluorescent/photoacoustic probe, CDIA, for monitoring •OH in AKI and demonstration of the strategy for HTS of antioxidant natural products to attenuate AKI.

Herein, a •OH activatable fluorescent/photoacoustic probe (termed CDIA) has been designed and synthesized for imaging of AKI in murine model. Previous research has shown that the reaction of •OH with a bulky phenol like diiodophenol favors an electron transfer reaction, resulting in the formation of a phenoxyl radical that leads to the decomposition of aromatic compounds.^[^ ^28^ ^]^ Additionally, taking cues from the efficient reaction between •OH and aspirin,^[^ ^29^ ^]^ a turn‐on molecular probe can be rationally designed and synthesized. Thus, the probe CDIA is designed to have two components: 1) the signaling moiety near‐infrared (NIR) hemi‐cyanine dye^[^ ^30^ ^]^ and 2) a target‐responsive substrate iodosalicylic acid, which is recognized and cleaved by •OH (**Figure** **1**). The probe is initially non‐fluorescent and non‐photoacoustic in an aqueous solution, because the hydroxyl group is in a “caged” state with diminished electron‐donating ability. Upon exposure to •OH, the iodosalicylic acid component of the molecule undergoes a specific reaction to release the free dye (CyOH) and transition into an “uncaged” state. As a result of the enhanced electron‐donating ability from the oxygen atom of the free dye, the product exhibits turned‐on NIR fluorescence and PA signals (Figure 1). Thus, the probe can be activated by •OH to produce its NIR fluorescence and PA signal. With its exceptional sensitivity and selectivity to •OH, CDIA has proven effective in visualizing the endogenous •OH production in living cells and mice subjected to various stimuli, without being affected by other ROS. Moreover, CDIA can be applied for the high‐throughput screening (HTS) of natural •OH scavengers from herbal medicines in live cells, which could reveal further mechanisms involved in the chemical regulation of natural products to attenuate AKI.

## 2 Results and Discussion

### 2.1 Synthesis and Characterization of CDIA

The construction of CDIA involved three main steps: i) Synthesis of the symmetric cyanine dye 1 (CyCl), ii) synthesis of the hemi‐cyanine precursor 2 (CyOH), and iii) synthesis of the probe CDIA by conjugating CyOH and 3,5‐diiodosalicyclic acid. The hemi‐cyanine precursor CyOH was via a retro‐knoevenagel reaction using the symmetric cyanine dye CyCl and resorcinol,^[^ ^31^ ^]^ followed by its reaction with a 3,5‐diiodosalicyclic acid to afford the hemi‐cyanine CDIA. According to the integration ratio of proton in the ^1^H NMR spectrum (Figures S1–S4, Supporting Information), ^13^C NMR spectrum (Figure S5, Supporting Information) and mass spectrum (Figure S6, Supporting Information), the dual‐mode probe CDIA had been successfully conjugated with a molecular weight of 674.55.

### 2.2 Spectral Response of CDIA to •OH In Vitro

**Figure 2:**
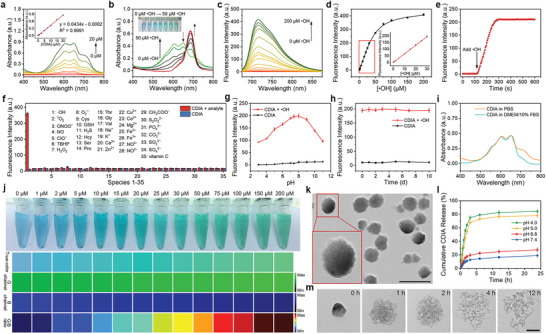
In vitro detection of •OH using CDIA. a) UV–vis–NIR absorption spectra of CDIA with different concentrations. Inset: The standard curve of CDIA determined by measuring the absorbance at 650 nm. b) UV–vis–NIR absorption spectra of CDIA (10 µm) in the absence or presence of •OH with different concentrations in PBS (10 mm, pH 7.4) at 37 °C. Inset: The corresponding white light images in the absence or presence of different concentrations •OH. c) Fluorescence spectra of CDIA (10 µm) in the absence or presence of •OH with different concentrations. d) Fluorescence intensity of CDIA as a function of •OH concentration. Inset: Linear correlation between the fluorescence intensity and •OH concentration. All measurements were acquired in PBS (10 mm, pH 7.4) at 37 °C, λex/em = 650/720 nm. e) Time‐dependent fluorescence changes of 10 µM CDIA towards 100 µm •OH in PBS buffer (10 mm, pH 7.4) at 37 °C. f) Fluorescence responses of 10 µm CDIA toward various analytes: 1) 100 µm •OH; 2) 100 µm 1O2; 3) 100 µm ONOO−; 4) 100 µm NO; 5) 100 µm ClO−; 6) 100 µm TBHP; 7) 100 µm H2O2; 8) 30 µm O2•−; 9) 5 mm Cys; 10) 5 mm GSH; 11) 100 µm H2S; 12) 100 µm Hcy; 13) 100 µm Ser; 14) 100 µm Pro; 15) 100 µm Thr; 16) 100 µm Gly; 17) 100 µm Val; 18) 100 µm Na+; 19) 100 µm K+; 20) 100 µm Ca2+; 21) 100 µm Zn2+; 22) 100 µm Cu2+; 23) 100 µm Co2+; 24) 100 µm Mg2+; 25) 100 µm Fe2+; 26) 100 µm Fe3+; 27) 100 µm NO3−; 28) 100 µm NO2−; 29) 100 µm CH3COO−; 30) 100 µm S2O32−; 31) 100 µm PO43−; 32) 100 µm CO32−; 33) 100 µm SO32−; 34) 100 µm SO42−; 35) 100 µm vitamin C. g) Effect of pH on the fluorescence of CDIA (10 µm) in the absence or presence of 100 µM •OH in PBS buffer (10 mm, pH 7.4) at 37 °C. h) Fluorescence intensity of CDIA for long‐term‐stability study. i) UV–vis–NIR absorption spectra of stability study of CDIA in PBS or DMEM with 10% FBS. j) True‐color and G/B ratio images calculated of the green‐ and blue‐ channels at different •OH concentrations from 0 to 200 µm. k) TEM image of CDIA@LMWC NP. Scale bar: 100 nm. l) In vitro release profile of CDIA from CDIA@LMWC NP at different pH conditions. m) TEM images of CDIA@LMWC NP in PBS buffer (10 mM, pH 5.0) at different time points. Scale bar: 50 nm. Data represent mean ± SD (*n* = 5).

In order to confirm the response of CDIA to •OH, optical spectra analyses were utilized to track the changes of the probe both qualitatively and quantitatively. Initially, CDIA was non‐fluorescent and had an NIR absorption peak at 650 nm (**Figure** **2**a). Upon exposure to •OH at 37 °C, the probe exhibited a significant decrease in the absorption peak at 650 nm and a new peak at 690 nm emerged (Figure 2b), corresponding to the free dye CyOH. Additionally, CDIA demonstrated an increase in fluorescence intensity at 720 nm (Figure 2c; Figure S7, Supporting Information) and a rise in fluorescent quantum yield to 0.27 following its reaction with •OH (Table S1, Supporting Information). This was attributed to the effective reaction between •OH and iodosalicylic acid subunit, leading to the formation of a CyOH moiety with a strong electron‐donating phenolate group of the fluorophore and thus strong fluorescence. Good linearity between the fluorescence intensities (Figure 2d) and the concentrations of •OH was observed with a limit of detection (LOD) of 5.30 nm (Figure S8, Supporting Information), indicating its ultrasensitivity towards •OH compared with other previously reported works (Table S2, Supporting Information). The probe exhibited remarkable sensitivity and fast response to Fenton reagent, completing the oxidation within 3 min (Figure 2e). In addition, CDIA showed negligible NIR fluorescence responses to other interfering analytes, including other ROS and metal ions (Figure 2f; Figures S9 and S10, Supporting Information), indicating its high specificity of NIR activation channel towards •OH. The probe functioned well across the physiological pH range of 6.0–9.0 (Figure 2g; Figure S11, Supporting Information) and demonstrated excellent stability (Figure 2h,i; Figure S12, Supporting Information), suggesting its potential for detecting •OH in living organisms. The PA properties of CDIA were also studied, and the PA signal of the probe at 690 nm significantly increased in the presence of •OH, consistent with the absorption changes upon treatment with •OH (Figure S13, Supporting Information). All the results confirmed that CDIA had excellent photoactivation properties to detect •OH in vitro. Furthermore, high‐performance liquid chromatography (HPLC) and mass spectrum further demonstrated that the probe (HPLC retention time, CDIA: T_R_ = 6.949 min) was totally hydrolyzed and converted into free hemi‐cyanine dye CyOH (T_R_ = 10.596 min) after incubation with •OH (Figure S14, Supporting Information).

We proceeded to test whether the probe was capable of enabling ratiometric imaging of •OH in vitro using RGB image analysis with a smartphone.^[^ ^32^ ^]^ The color of CDIA changed noticeably from blue to green upon reacting with various concentrations of •OH (Figure 2j). True‐color photos captured with a smartphone were separated into red (R), green (G), and blue (B) channels and digitized through an image‐processing algorithm. The ratio of green and blue channel intensities (G/B ratio) could then be used to measure the concentration of •OH. Therefore, it could readily detect concentrations of •OH as low as 2 µm with the naked eye from the G/B ratio, indicating that CDIA has a wide range of applications for identifying •OH.

Numerous chemical compounds, including biologically significant ones such as DMSO,^[^ ^33^ ^]^ thiourea,^[^ ^34^ ^]^ glutathione (GSH),^[^ ^35^ ^]^ NAC,^[^ ^36^ ^]^ 2,2,6,6‐tetramethyl‐1‐piperidinyloxy (TEMPO),^[^ ^37^ ^]^ and thyroid hormones,^[^ ^38^ ^]^ like triiodothyronine (T_3_) and thyroxine (T_4_), that have the capability to scavenge •OH. Unfortunately, a reliable and sturdy technique for measuring their reactivity has not yet accessible, which obstructs the understanding of many essential mechanisms in both biological and chemical systems.^[^ ^39^ ^]^ The use of CDIA probe makes it possible to calibrate and quantify the scavenging efficiency of known scavengers, thereby facilitating deeper exploration of cellular antioxidant capability. By detecting the response of CDIA to Fenton reagents in the presence of different scavenger concentrations, we were able to determine their scavenging efficiency. Figures S15 and S16 (Supporting Information) showed that ranking of scavenging effectiveness was as follows: T_4_ > T_3_ > TEMPO > GSH ≈ thiourea > NAC ≫ DMSO. The reason for the excellent effectiveness of T_4_ and T_3_ was their ability to directly react with •OH, which was similar to CDIA. Therefore, we utilized the extremely sensitive CDIA probe to validate and compare the antioxidant properties of several typical •OH scavengers in vitro.

### 2.3 NIR Fluorescent Response of CDIA to Dynamic Changes of •OH in Living Cells

**Figure 3:**
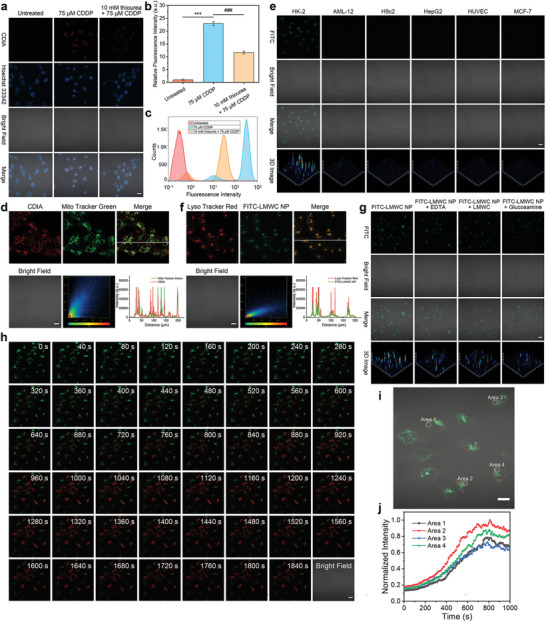
Imaging •OH fluxes in living cells with CDIA. a) Confocal fluorescence imaging of HK‐2 cells with untreated, 75 µm CDDP or pre‐treated with 10 mm thiourea for 2 h before 75 µm CDDP for 24 h, then incubated with 10 µm CDIA at 37 °C for 30 min. b) Fluorescence intensity plots of HK‐2 cells for different groups. Data represent mean ± SD (*n* = 5), ^*^ *P* < 0.05, ^**^ *P* < 0.01, ^***^ *P* < 0.001, #*P* < 0.05, ##*P* < 0.01, ###*P* < 0.001; one‐way ANOVA with multiple comparisons test. c) Flow cytometric assays of HK‐2 cells for different groups. d) Confocal fluorescence images of 150 µm CDDP‐treated HK‐2 cells co‐stained with CDIA and Mito Tracker Green (Pearson's correlation Rr = 0.8237, the value of the Pearson's correlation coefficient is between 1 and −1. The closer to 1, the more perfect correlation; the closer to −1, the more complete negative correlation). e) Confocal fluorescence imaging of HK‐2, AML‐12, H9c2, HepG2, HUVEC, and MCF‐7 cells incubated with 20 µg mL^−1^ FITC‐LMWC NP at 37 °C for 2 h. f) Confocal fluorescence images of HK‐2 cells co‐stained with CDIA and Lyso Tracker Red (Pearson's correlation Rr = 0.7703). g) Competitive inhibition of cellular uptake of 20 µg mL^−1^ FITC‐LMWC NP by different inhibitors (1 µm EDTA, 50 µg mL^−1^ LMWC, or 50 µg mL^−1^ glucosamine) at 37°C for 2 h. h) Time lapse monitoring of CDIA@FITC‐LMWC NP in CDDP stimulated HK‐2 cells. Cells were successively incubated with CDDP for 24 h and CDIA@FITC‐LMWC NP for 2 h, the images were recorded every 40 s per frame. i) Merged fluorescence and bright field image of CDIA@FITC‐LMWC NP in CDDP stimulated HK‐2 cells. j) Time course of fluorescence intensity collected at circles 1–4 shown in i), corresponding to the areas (1–4). Scale bar: 20 µm.

After confirming that the probe has good cytocompatibility (Figure S17, Supporting Information), CDIA was applied to detect •OH in cultured cells. HK‐2 cells (an immortalized proximal tubule epithelial cell line from normal adult human kidney) were subjected to nephrotoxic drug cisplatin (cis‐diammineplatinum dichloride, CDDP).^[^ ^40^ ^]^ The half maximal inhibitory concentration (IC_50_) was defined as the concentration which was necessary to induce the apoptosis percentage to 50% (Figure S18, Supporting Information). HK‐2 cells were treated with CDDP at the corresponding IC_50_ for 24 h, respectively. In control groups, HK‐2 cells were preloaded with 10 mm thiourea (an acknowledged scavenger of •OH) for 2 h before treated CDDP. All groups were then incubated with CDIA (10 µm) for NIR fluorescence imaging. After incubation for 30 min, the NIR fluorescence of CDDP‐treated HK‐2 cells were remarkably enhanced by 22.97‐fold (Figures 3a–c), confirming the feasibility of CDIA for drug‐induced cell injury imaging. Co‐staining experiments demonstrated that CDIA, following •OH activation, was predominantly localized in the mitochondria region (**Figure** **3**d; Figures S19–S25, Supporting Information). This finding was consistent with previous studies in which cationic fluorescent probes, such as cyanine dyes, were found to exhibit selective accumulation within the mitochondria of living cells.^[^ ^41^, ^42^ ^]^ Conversely, a significant reduction in fluorescence intensity was observed in HK‐2 cells pre‐treated with thiourea, further verifying that the fluorescence amplification originated from CDIA after activation by overexpressed •OH in HK‐2 cells. Meantime, monitoring of intracellular •OH over time using CDIA showed no evidence of photoactivating or photobleaching (Figures S26 and 27 and Movie S1, Supporting Information).

In order to detect endogenous •OH in a broad range of contexts, we investigated the usage of CDIA in various model cells. Specifically, we evaluated its performance in RAW264.7 mouse macrophages and HeLa cells by treating them with PMA, a protein kinase C (PKC) activator that induces endogenous •OH.^[^ ^39^ ^]^ The cells were co‐incubated with PMA (at concentrations of 200 or 500/ng mL) for 4 h, followed by incubation with 10 µm CDIA at 37 °C for 30 min. The results showed a significant increase in NIR fluorescence signal compared to the control group (Figures S28, S29, and S31a,b, Supporting Information). Additionally, we tested the effectiveness of CDIA in detecting OS in HepG2 cells caused by acetaminophen (APAP), which was a component of many cold medications that could cause liver injury and OS when taken in excess.^[^ ^43^, ^44^ ^]^ The results showed a dose‐dependent increase in NIR fluorescence signal with different concentrations of APAP (Figures S30 and S31c, Supporting Information). These findings suggested that CDIA was a highly effective tool for intracellular •OH imaging.

The PA signals and spectra of CDIA were examined after it was incubated with cells. Figure S32 (Supporting Information) showed that the PA signal at 690 nm of HK‐2 cell pellets treated with CDDP was higher than that of HK‐2 cell pellets treated with both CDDP along with thiourea. The PA signals of RAW264.7, HeLa, and HepG2 cell pellets were consistent with the results obtained from confocal imaging (Figures S33–S35, Supporting Information). These above findings not only demonstrated that CDIA could be selectively activated by •OH produced by cells, but also indicated its potential for use in PA imaging in living cells.

Low molecular weight chitosan (LMWC),^[^ ^45^ ^]^ a copolymer derived from chitin and composed of *D*‐glucosamine and *N*‐acetylglucosamine, has been selected as a nanocarrier for renal targeting owing to its biocompatibility and biodegradability.^[^ ^46^ ^]^ LMWC has the ability to bind to the megalin receptor,^[^ ^47^, ^48^ ^]^ which is predominantly present on renal tubular epithelial cells. CDIA was encapsulated in LMWC to create the nanoprobe CDIA@LMWC NP. Theoretically, CDIA@LMWC NP remained stable under physiological conditions and could be selectively internalized into the lysosome through megalin receptor‐mediated endocytosis, after which CDIA was released due to the acidic pH environment. Utilizing an ionic cross‐linking technique (Figure S36, Supporting Information),^[^ ^49^ ^]^ the CDIA@LMWC NP had been synthesized, and exhibited a uniformly dispersed, spherical morphology measuring 50 nm on average, as observed through transmission electron microscopy (TEM) (Figure 2k). The average hydrodynamic diameter of 58.3 ± 1.9 nm, as observed by dynamic light scattering (DLS) analysis, was attributed to the presence of hydration layers (Figure S37a, Supporting Information). The targeting efficacy of the LMWC‐based nanocarrier was evaluated using an FITC‐labeled LMWC nanoparticle, which demonstrated good linearity in fluorescence intensity and successful conjugation of FITC to LMWC NP. To assess renal cell targeting, an FITC‐labeled LMWC nanoparticle (FITC‐LMWC NP) had been synthesized. The successful conjugation of FITC to LMWC NP was validated by a notable fluorescence peak observed at 518 nm in the fluorescence spectra (Figure S38, Supporting Information) and a strong correlation between the concentration of the sample and the fluorescence intensity at 518 nm within the range of 0–10 µg mL^−1^. The amount of FITC that had been conjugated to the FITC‐LMWC NP was measured to be 63.84 ± 0.22 µg mg^−1^ using a standard curve method (Figure S39, Supporting Information). The zeta potentials of LMWC NP, FITC‐LMWC NP, and CDIA@LMWC NP were measured to be +37.4, +36.8, and +32.9 mV, respectively (Figure S37 and Table S3, Supporting Information), indicating good stability and transportation in glomerular filtration (Figure S40, Supporting Information). These above findings suggested that LMWC could serve as a suitable nanocarrier for renal targeting.^[^ ^50^ ^]^

To determine the encapsulation efficiency (EE) and loading content (LC) of the nanoprobe, the ultraviolet–visible–near infrared (UV–vis–NIR) standard curves of CDIA were used (Figure 2a). The EE and LC of CDIA were then measured to be 83.62 ± 0.17% and 10.57 ± 0.04%, respectively (Table S4, Supporting Information). In vitro release behaviors of the nanoprobe CDIA@LMWC NP was investigated using the dialysis method at different pH levels.^[^ ^51^ ^]^ The nanoprobe remained stable over a 24‐hour incubation period at pH 7.4 and 6.8 (Figure 2l), while its release rate was significantly accelerated in an acidic environment of pH 5.0 (Figure 2m). After incubation for 4 h, the accumulative release rate of CDIA reached 72.96 ± 0.53%, indicating effective delivery and release of the nanoprobe into the acidic lysosome of renal cells. It is worth noting that the introduction of LMWC did not have a noticeable effect on the reaction of CDIA and •OH (Figure S41, Supporting Information).

With the good biocompatibility (Figure S42, Supporting Information), we further optimized the incubation time and concentration of FITC‐LMWC NP by using confocal laser scanning microscopy. The results demonstrated that fluorescence signals of FITC increased gradually with improving incubation time, and almost no extracellular fluorescence was observed (Figure S43, Supporting Information). Thus, a concentration of 20 µg mL^−1^ of FITC‐LMWC NP and CDIA@LMWC NP with an incubation time of 2 h were chosen for subsequent experiments. In order to investigate the cell specificity of cellular uptake, HK‐2 cells, HepG2 cells, alpha mouse liver 12 AML‐12 cells, rat heart muscle H9c2 cells, human umbilical vein endothelial HUVEC cells, human mammary cancer MCF‐7 cells were incubated with FITC‐LMWC NP. The selective accumulation of FITC‐LMWC NP in HK‐2 cells was shown by the increased green fluorescence observed through confocal fluorescence imaging. In contrast, minimal fluorescence could be observed in the other cell lines (Figure 3e; Figure S44, Supporting Information). To investigate the subcellular distribution of the nanocarrier in cells, FITC‐LMWC NP was incubated with HK‐2 cells and co‐stained with Lyso Tracker Red, a lysosome probe, and Hoechst 33342, a nucleus dye. By confirming the localization of FITC‐LMWC NP in the lysosomal compartment (Figure 3f; Figure S45, Supporting Information) and ruling out mitochondrial regions (Figure S46, Supporting Information), the yellow fluorescence resulting from the overlap of green FITC and red LysoTracker signals provided evidence for the targeting capability of nanocarrier to HK‐2 cells. Further investigation was carried out to understand the mechanism underlying the increased cellular uptake of FITC‐LMWC NP in HK‐2 cells. Studies using ethylene diamine tetraacetic acid (EDTA), free LMWC, or glucosamine as megalin receptor inhibitors significantly reduced the uptake of FITC‐LMWC NP (Figure 3g; Figure S47, Supporting Information),^[^ ^52^ ^]^ demonstrating that the LMWC nanocarrier was proposed to have been taken up by HK‐2 cells via an endocytosis pathway that was mediated by the megalin receptor.

The production of •OH induced by nephrotoxic drugs in HK‐2 cells was quantified using the commercial ROS probe dichlorodihydrofluorescein diacetate (H_2_DCFDA). HK‐2 cells were treated with CDDP, aristolochic acid I (AAI), and citrinin (CTN) at IC_50_ concentrations for 24 h (Figure S14, Supporting Information), respectively.^[^ ^53^, ^54^ ^]^ The fluorescence intensity in HK‐2 cells experienced a gradual increase with increasing concentration of the nephrotoxic drugs (Figure S48, Supporting Information). Confocal fluorescence imaging and flow cytometric assays were then performed to evaluate the feasibility of using CDIA@LMWC NP for imaging nephrotoxic drug‐induced injury in HK‐2 cells. The cells were incubated with CDDP for 24 h, and the fluorescent changes of 20 µg mL^−1^ CDIA@FITC‐LMWC NP‐loaded HK‐2 cells were monitored in real‐time using confocal fluorescence imaging (Movie S2, Supporting Information). Initially, bright green fluorescence was observed in the lysosome (Figure 3h), indicating good lysosomal location and cell viability. Subsequently, an increment in the red fluorescence of CDIA within HK‐2 cells was observed in the confocal fluorescence images, which signified an escalation in •OH production throughout CDDP treatment. By collecting the fluorescence signals in the mitochondria (Figure 3i), the fluorescence changes could be observed (Figure 3j), demonstrating the excellent application of CDIA@FITC‐LMWC NP for discriminative imaging of CDDP‐induced HK‐2 cell injury. Meanwhile, CDIA@LMWC NP was also utilized for monitoring •OH generation during the process of nephrotoxic drugs AAI and CTN stimulation. The NIR fluorescence of AAI‐ and CTN‐treated HK‐2 cells were remarkably enhanced by 22.63‐ and 24.57‐fold, respectively (Figures S49 and S50, Supporting Information). Meanwhile, a remarkable decrease in fluorescence intensity was observed in HK‐2 cells pretreated with thiourea, which confirmed the feasibility of CDIA@LMWC NP for drug‐induced cell injury imaging.

Likewise, PA images and spectra of CDIA@LMWC NP after incubation with HK‐2 cells were also studied. On the premise that LMWC would not interfere with the reaction between the probe CDIA and •OH (Figure S51, Supporting Information), PA signal of HK‐2 cell pellets after treatment with AAI or CTN at 690 nm was higher than the PA signal of HK‐2 cell pellets treated with AAI or CTN along with thiourea (Figures S52 and S53, Supporting Information). These above results displayed that the dual‐mode nanoprobe CDIA@LMWC NP could be employed to visualize the dynamic changes of •OH during nephrotoxic drugs‐induced renal injury.

### 2.4 High‐Throughput Screening of Natural Products

**Figure 4:**
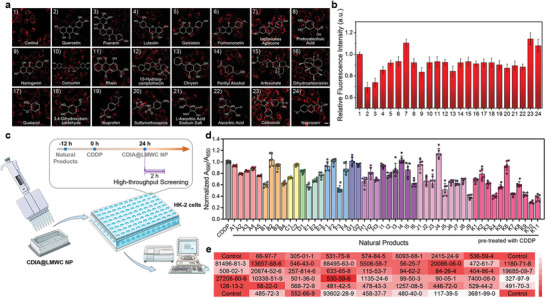
HTS of natural products with CDIA@LMWC NP in living HK‐2 cells for AKI. a) HK‐2 cells were pretreated with 50 µm various natural antioxidants for 12 h before 75 µm CDDP for 24 h, and then images were obtained by high‐content analysis after incubation with 20 µg mL^−1^ CDIA@LMWC NP at 37 °C for 2 h. Scale bar: 20 µm. b) Quantification performed on the relative ratio of fluorescence intensity shown in (a). Data represent mean ± SD (*n* = 3). c) Diagram of the strategy for HTS of antioxidant natural products using microplate reader. d) Antioxidant effects of natural products on •OH scavenging by utilizing CDIA@LMWC NP. A: coumarins, A1: psoralen, A2: 6,7‐dihydroxycoumarin, A3: esculin hydrate, A4: fraxetin, A5: 6‐hydroxycoumarin; B: sesquiterpenes, B1: dihydroartemisinin, B2: curdione, B3: alantolactone, B4: artesunate; C: monoterpenes, C1: catalpol, C2: perillyl alcohol; D: diterpenes, D1: andrographolide; E: triterpenes, E1: oleanolic acid, E2: saikosaponin D, E3: ginsenoside Re; F: others, F1: cantharidin, F2: diosbulbin B, F3: astaxanthin, F4: limonin; G: steroids, G1: ursodeoxycholic acid, G2: testosterone; H: quinones, H1: tanshinone IIA, H2: 5‐hydroxy‐2‐methyl‐1,4‐naphthoquinon, H3: rhein; I: alkaloids, I1: berberine, I2: sinomenine, I3: piperine, I4: rutecarpine, I5: capsaicin, I6: 10‐hydroxy camptothecin; J: phenolics, J1: polydatin, J2: salidroside, J3: resveratrol, J4: sinapic acid, J5: ferulic acid, J6: protocatechuic acid, J7: guaiacol, J8: p‐hydroxy‐cinnamic acid, J9: chlorogenic acid; K: flavones, K1: (‐)‐epicatechin gallate, K2: genistein, K3: myricetin, K4: luteolin, K5: formononetin, K6: isoflavone aglycone, K7: naringenin, K8: curcumin, K9: chrysin, K10: Que, K11: Pue. Data represent mean ± SD (*n* = 6). e) The CAS numbers of the natural compounds corresponding to each microplate for antioxidant screening.

With the impressive results obtained using CDIA@LMWC NP for live‐cell imaging, we proceeded to investigate its potential as a screening tool for identifying antioxidants that could reduce •OH formation. To this end, we established a straightforward and effective HTS platform using CDIA@LMWC NP. Specifically, HK‐2 cells were pre‐treated with various natural products with potential antioxidative activity at 50 µm for 12 h before administering 75 µm of CDDP for 24 h. Subsequently, images and quantitative analysis of endogenous •OH levels were conducted after the cells treated with 20 µg mL^−1^ CDIA@LMWC NP (**Figure** **4**a).

By utilizing this approach, the rapid and quantitative detection of endogenous •OH generation was ensured (Figure 4b). Our findings demonstrated that partial natural products selected were able to significantly reduce fluorescence signals, indicating a down‐regulation of •OH generation. Of particular note, puerarin (Pue), a key active ingredient found in the herbal medicine Kudzu root,^[^ ^55^ ^]^ was shown to be highly effective in reducing fluorescence signals compared to the untreated control. This phenomenon suggested that this flavonoid compound could have potential as an agent for controlling the accumulation of nephrotoxic drug‐generated •OH in live cells. The results demonstrated that CDIA@LMWC NP could serve as a valuable tool for screening natural compounds that regulate endogenous •OH variations in a simple yet powerful way. Most kits for the activity assay and screening of biological molecules work on the basis of optical density (OD) values at specific wavelengths for the designed chromogenic reagents. Here, to achieve more portability and efficiency, we used CDIA@LMWC NP with high sensitivity to detect •OH content and establish a HTS system to rapidly discover natural products to attenuate AKI (Figure 4c). In this work, 50 natural compounds with a variety of chemical skeletons (e.g., coumarins, sesquiterpenes, monoterpenoids, diterpenoids, triterpenoids, flavones, steroids, alkaloids, polyphenols, and quinones) from a natural compound library have been evaluated for their antioxidant effects on AKI (Table S5, Supporting Information). Compared with the control group, weak absorption intensity ratio at 690/650 nm (*A*_690_/*A*_650_) of CDIA was observed for the wells corresponding to compounds J9 (chlorogenic acid), K10 (quercetin, Que), and K11 (puerarin, Pue) (Figure 4d), indicating potential •OH scavenging effect. In comparison with the control group, the relative *A*_690_/*A*_650_ of each well was interpreted intuitively as shown in Figure 4e. Because the •OH scavenging effect of chlorogenic acid and Que had been clearly demonstrated by some previous works,^[^ ^56^, ^57^ ^]^ compound K11 (Pue) was selected as a potential therapeutic agent to attenuate AKI.

### 2.5 In Vivo Duplex Imaging of AKI

**Figure 5:**
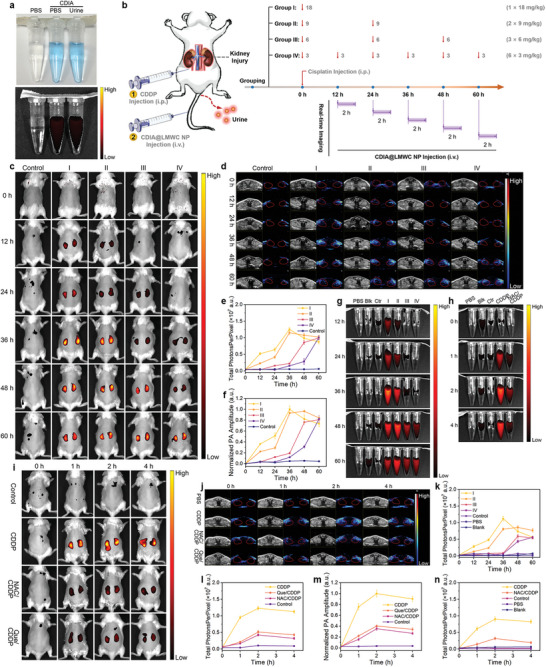
NIR fluorescence and PA imaging of DIAKI in vivo. a) White light and NIR images of CDIA in PBS and CDIA in the urine excreted from living mice. The corresponding NIR images acquired at 720 nm on excitation at 650 nm with Tanon ABL X6 imaging system. b) Schematic diagram of CDDP treatment in different groups of mice and the timeline of experimental design. Mice were randomly assigned to four experimental groups and a control group. The mice in four groups were treated intraperitoneally with CDDP at the same total dosage of 18 mg k^−1^ g body weight. Mice in group I were treated once with CDDP at a dosage of 18 mg k^−1^ g; mice in group II were treated twice with CDDP at each dosage of 9 mg k^−1^ g; mice in group III were treated thrice with CDDP at each dosage of 6 mg k^−1^ g; mice in group IV were treated with CDDP at each dosage of 3 mg k^−1^ g for six times. The mice in control group were treated with PBS. Red arrows indicate for the CDDP injection at different time points. Time‐dependent fluorescence and PA imaging were recorded for 2 h after i.v. injection of 10 mg k^−1^ g CDIA@LMWC NP, *n* = 5 for each group. c) In vivo NIR fluorescence images of mice in each group at different time points post‐treatment with CDDP and CDIA@LMWC NP. d) In vivo PA images of mice kidney in each group treatment with different doses of CDDP, followed by i.v injection of CDIA@LMWC NP (10 mg k^−1^ g body weight) at different time points. e) Fluorescence intensities of mice kidney in (c). f) The normalized PA amplitude of mice kidney in (d). g) Fluorescence images of mice urine under the excitation at 650 nm in each group treatment with different doses of CDDP. h) Fluorescence images of mice urine under the excitation at 650 nm at different post‐treatment timepoints. Representative NIR images (i) and PA images (j) of living mice after injection of CDIA@LMWC NP at different post‐treatment timepoints. The mice were treated with saline, 36 h post‐treatment of 18 mg k^−1^ g CDDP, or 10 mg k^−1^ g NAC/Que (i.p. injection) 3 days prior to CDDP administration. k) Fluorescence intensities of mice urine in (g). l) Fluorescence intensities of mice kidney in (i). m) The normalized PA amplitude of mice kidney in (j). n) Fluorescence intensities of mice urine in (h). Data represent mean ± SD, *n* = 5 independent mice.

CDIA had almost identical fluorescence in the PBS and urine (**Figure** **5**a; Figure S54, Supporting Information), indicating the excellent stability of CDIA in mice. Moreover, the absence of acute or chronic toxicity of CDIA and CDIA@LMWC NP was confirmed in mice (Figure S55, Supporting Information), along with negligible hemolysis toxicity to red blood cells (Figure S56, Supporting Information), indicating the potential of CDIA@LMWC NP for in vivo applications, specifically for monitoring renal diseases. In order to explore the pharmacokinetics of CDIA@LMWC NP, the blood concentration of CDIA@LMWC NP was examined using HPLC after a solitary intravenous injection in live mice. The blood concentration curves revealed a two‐compartment pattern in the in vivo kinetics of CDIA@LMWC NP (Figure S57, Supporting Information). The elimination half‐life (t_1/2β_) for the blood concentration of CDIA@LMWC NP was calculated to be 65.35 min, underscoring its efficient removal from the body through systemic clearance. The capacity of CDIA@LMWC NP for duplex imaging of AKI was assessed in a mouse model utilizing CDDP, a known nephrotoxic cancer treatment drug, as the model drug.^[^ ^58^ ^]^ A total of five groups, including a control group receiving saline and four treatment groups receiving the same total CDDP dosage but with different dosing frequencies, were established by random assignment of mice in this experiment. (Figure 5b).^[^ ^59^ ^]^ CDIA@LMWC NP was injected intravenously (i.v.) after CDDP administration, and in vivo imaging of the mice was conducted within the NIR window at various time intervals. Simultaneously, transurethral catheters were used to collect urine samples from the mice, which were subsequently imaged while being exposed to 650 nm irradiation. Mice administrated with a high initial dose of CDDP exhibited signs of kidney damage after 12 h (Figures 5c,d, Group I and Group II). There was a gradual increase in the NIR fluorescence signals and PA intensities in the kidneys over time, which suggested renal damage progressed from mild to severe levels (Figures 5e,f; Figure S58, Supporting Information). Group IV that received a low initial dose of CDDP treatment experienced delayed onset of kidney dysfunction, while repeated doses of CDDP also resulted in kidney damage. The urine of the mice displayed comparable fluorescence signal variations to those observed in the in vivo NIR fluorescence (Figures 5g,k). Whereas the NIR fluorescence and PA amplitude in the kidney signaled the status of kidney injury that had built up over time, the urine signal indicated the degree of transient renal damage. The pathological analysis of kidney slices further validated the above findings (Figure S59, Supporting Information). Notably, the use of a dual‐mode imaging approach for monitoring kidney dysfunction allowed for the identification of CDDP‐induced kidney injury 36 h prior to clinical diagnosis based on BUN and sCr levels (Figure S60, Supporting Information), providing additional confidence for AKI diagnosis. The LMWC‐targeted kidney monitoring method facilitated the timely detection of renal disease and furnished valuable information on the evolution of kidney injury during treatment, which could be potentially contributed to treatment optimization.

In order to further explore the effect of remission on renal injury, the control group of mice received treatment with saline or nephroprotective antioxidant Que or NAC (10 mg kg^−1^ day^−1^, intraperitoneal (i.p.) injection) 3 d prior to CDDP administration. At different post‐treatment timepoints, whole‐body longitudinal NIR fluorescence imaging (Figure 5i) and renal site PA imaging (Figure 5j) were conducted simultaneously. The NIR fluorescence and PA intensities increased gradually over time and peaked at 2 h after injection. However, these signals were significantly reduced in mice that received pre‐treatment with Que or NAC (Figure 5l,m). Similar results were observed in the analysis of the mice urine (Figures 5h,n). To confirm that CDIA@LMWC NP was activated in situ in the kidneys, ex vivo fluorescence images of the kidneys and other organs outside the kidneys were taken. Fluorescence signals were detected solely in the excised kidneys of mice 3 h after the injection of CDIA@LMWC NPs, consistent with the in vivo NIR imaging data (Figure S61, Supporting Information). This signal was 8.25 times greater than that observed in the control mice. The NIR fluorescence and PA signals indicated that CDIA@LMWC NP accumulated efficiently in the kidney region and reacted with •OH, which was overexpressed in AKI mice.

### 2.6 Molecular Mechanism Responsible for the Protective Effects of Pue on AKI

**Figure 6:**
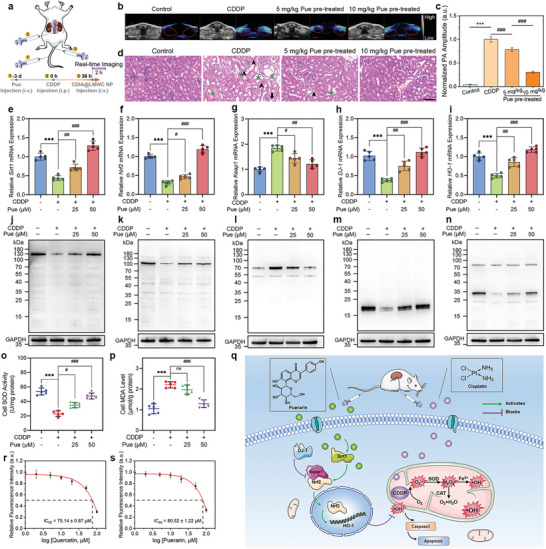
The counteraction of Pue against CDDP‐induced AKI. a) Schematic diagram of administration of mice. Mice were treated with saline, 18 mg k^−1^ g CDDP, or Pue (5 mg k^−1^ g or10 mg k^−1^ g, i.v. injection) 3 days prior to CDDP administration. b) In vivo PA images of mice kidney with different administrations. c) The normalized PA amplitude of mice kidneys in (b). Data represent mean ± SD, *n* = 5 independent mice. d) H&E staining images of the kidneys from mice in different administration groups. Scale bar: 100 µm. Relative mRNA expressions of e) Sirt1, f) Nrf2, g) Keap1, h) DJ‐1, and i) HO‐1 in HK‐2 cells after respective treatments with CDDP, and Pue plus CDDP. Western blot analysis of the j) Sirt1, k) Nrf2, l) Keap1, m) DJ‐1, and n) HO‐1 levels in HK‐2 cells after treatments, *n* = 5 independent experiments. Measurement of o) SOD activities and p) MDA levels in HK‐2 cells following various treatments. q) Schematic illustration of the remaining quantities of Pue reducing the levels of •OH by decomposing CDDP‐induced •OH into H2O and O2, via activating the Sirt1/Nrf2/Keap1 signaling pathway. Chemical •OH scavenging by r) Que and s) Pue with CDIA (10 µm) in PBS buffer at 37 °C, λex/em = 650/720 nm. Data represent mean ± SD (*n* = 5), ^*^ *P* < 0.05, ^**^ *P* < 0.01, ^***^ *P* < 0.001, #*P* < 0.05, ##*P* < 0.01, ###*P* < 0.001; one‐way ANOVA with multiple comparisons test.

Initial research indicates that Pue has hepatoprotective properties in rats with CCl_4_‐induced liver damage by reducing OS and inflammation, leading to improved intrahepatic metabolism.^[^ ^60^ ^]^ Considering Pue was screened to be a potential antioxidant agent in vitro, we further tested the protective effects of Pue in vivo and explored the molecular mechanism for the antioxidant effects of Pue on AKI. Mice were treated with saline, 18 mg k^−1^g CDDP, or Pue (5 or 10 mg k^−1^g, i.v. injection) 3 days prior to CDDP administration (**Figure** **6**a). Dual‐mode images of mice kidney revealed that NIR fluorescence intensity and PA amplitude increased after CDDP administration while efficiently reduced if the mice were pre‐treated with Pue (Figures 6b,c; Figure S62, Supporting Information). Pathological analysis of the kidney slices showed the similar results (Figure 6d). In order to gain a deeper understanding of how Pue achieved the restoration of redox homeostasis in the progression of AKI, we examined alterations in crucial signaling molecules within the ROS regulatory network. Sirtuin 1 (Sirt1), a NAD^+^‐dependent histone deacetylase, is a critical component in maintaining cellular redox balance and resistance to OS.^[^ ^61^ ^]^ The transcription factor NF‐E2 p45‐related factor 2 (Nrf2), a transcription factor that plays a crucial role in cellular antioxidant pathways, is under the control of Sirt1 and can be reduced by excessive ROS levels.^[^ ^62^, ^63^, ^64^ ^]^ To evaluate the levels of Sirt1, Nrf2, and associated proteins such as haem oxygenase‐1 (HO‐1), DJ‐1, and Kelch‐like ECH‐associated protein 1 (Keap1) in vitro, both qRT‐PCR and western blot analyses were utilized.^[^ ^65^, ^66^, ^67^ ^]^ A qRT‐PCR analysis was performed to determine the status of the Sirt1/Nrf2 and DJ‐1/Nrf2/Keap1 axis. The results showed that the relative expressions of Sirt1, Nrf2, DJ‐1, and HO‐1 were significantly increased, while Keap1 was decreased at the mRNA level in HK‐2 cells that were pre‐treated with Pue (Figures 6e–i). These findings were further supported by the western blot analysis. The results revealed that Pue could elevate the expression of DJ‐1, the protein that was typically suppressed under OS.^[^ ^68^ ^]^ Subsequently, there was an upregulation of Sirt 1, Nrf2, and HO‐1, as well as a downregulation of Keap1 (Figures 6j–n; Figure S63, Supporting Information). No significant changes in the expression of these proteins were observed in HK‐2 cells treated with Pue alone (Figure S64, Supporting Information). After translocation to the nucleus, Nrf2 bound to antioxidant response elements, resulting in the upregulation of the antioxidant gene (HO‐1) and a decrease in •OH content. Moreover, DJ‐1 prevented the association of Nrf2 with its inhibitor protein, Keap1, and subsequent ubiquitination of Nrf2. In cases of low DJ‐1 expression, Nrf2 was ubiquitinated to a greater extent, which was associated with a decrease of Nrf2 protein. Furthermore, the treatment of CDDP inhibited the activity of superoxide dismutase (SOD) and increased the level of malondialdehyde (MDA) in HK‐2 cells. However, pre‐treatment of Pue could reversed the ROS‐related imbalance induced by CDDP (Figures 6o,p). The results suggested that Pue may exert a protective effect on cells by modulating the expressions of key proteins involved in antioxidant defense pathways. In addition, Pue was found to have an IC_50_ value of 80.52 ± 1.22 µm (Figure 6s) for •OH scavenging. Like Que (Figure 6r), Pue could directly react with •OH to achieve the effect of antioxidation. In brief, the data showed that Pue effectively decomposed the predominant cellular ROS, •OH, induced by CDDP into harmless substances like H_2_O and O_2_. Additionally, the remaining Pue activated the Sirt1/Nrf2/Keap1 signaling pathway. These results highlighted the significance of the reaction between Pue and •OH, as well as the crucial role of Pue in activating the Sirt1/Nrf2/Keap1 signaling pathway to quench ROS (Figure 6q). Ultimately, this protective mechanism prevented renal cell apoptosis and underscored the potential therapeutic value of Pue in protecting kidney.

## 3 Conclusion

To sum up, a probe has been created that can detect AKI through the activation of NIR fluorescence and PA signal in response to •OH. The dual‐mode imaging probe comprises a NIR hemi‐cyanine dye (CyOH) coupled with a •OH‐responsive substrate iodosalicylic acid. With high sensitivity and specificity toward •OH, CDIA possesses a fast activation kinetics and high biocompatibility. After introduction of LMWC for enhancing renal targeting, CDIA@LMWC NP accumulates in the kidney without any active intervention and triggers its fluorescence and PA signals to indicate the expression level of OS related to AKI. The positive time of 12 h using this probe is superior to that of 48 h for typical clinical assays including BUN and sCr detection. Moreover, CDIA@LMWC NP displays excellent ability for HTS of natural •OH scavengers from herbal medicines, uncovering additional mechanisms in the chemical regulation of natural products to attenuate AKI. Additional investigations have revealed that Pue, a natural antioxidant medication selected through screening, shields renal cells in cases of AKI by disintegrating CDDP‐induced •OH. Furthermore, it activates the Sirt1/Nrf2/Keap1 signaling pathway to regulate ROS‐related genes, which restores redox homeostasis in the renal cells. Given its effortless preparation and reactive activity that is dependent on the situation, we predict that the duplex imaging probe CDIA could be a potential option for detecting clinical AKI and other ROS‐related ailments in patients, thus illuminating the ROS‐related pathological process and improving the overall diagnostic efficacy.

## 4 Experimental Section

The detailed descriptions about experimental materials, physical characterizations, and the procedures of the synthesis, purification, and characterization of probe CDIA were described in the Supporting Information.

### Synthesis of CDIA@LMWC NP

0.25 mL of dimethyl sulfoxide (DMSO) containing 5 mg of CDIA was added to 4.25 mL of LMWC solution (5 mg of LMWC) to obtain a mixed solution. 2 mL 1.25 mg mL^−1^ TPP solution was added dropwise into the above mixture solution while stirring at 1000 rpm. The conjugation reaction was maintained for 1 h, and then the product was then purified by continuous dialysis (molar weight cutoff, MWCO 10000) against deionized water for 48 h. The final product was freeze‐dried and kept in desiccators for use.

### Confocal Fluorescence Imaging of •OH In Model Cells

Several different cell models were established: i) PMA (phorbol myristate acetate) was applied to induce endogenous •OH. RAW264.7 mouse macrophages and HeLa cells were exposed to PMA (200 or 500 ng mL^−1^) diluted with serum‐free medium for 4 h at 37 °C, respectively. ii) Acetaminophen (APAP) is a component of many cold medications, which may cause liver injury and oxidative stress in liver with overdose. HepG2 cells were exposed to APAP (5 or 10 mm) diluted with serum‐free medium for 24 h at 37 °C. iii) HK‐2 cells were exposed to 75 µm CDDP diluted with serum‐free medium for 24 h at 37 °C. iv) HK‐2 cells were exposed to 44 µm AAI diluted with serum‐free medium for 24 h at 37 °C. v) HK‐2 cells were exposed to 30 µm CTN diluted with serum‐free medium for 24 h at 37 °C. After incubation, the above cells were stained with CDIA (10 µm for 30 min) or CDIA@LMWC NP (20 µg mL^−1^ for 2 h), and then incubated with 5 µg mL^−1^ Hoechst 33342 for 25 min, rinsed three times with PBS (pH 7.4) to perform fluorescence imaging with a CLSM at stationary parameters including the laser intensity, exposure time, and objective lens. Hoechst 33342 was excited at 405 nm with a violet laser diode and the emission was collected from 420 to 480 nm. CDIA or CDIA@LMWC NP was excited with a 640 nm helium−neon laser and emission was collected from 650 to 750 nm. All images were digitized and analyzed by a ZEN imaging software.

### In Vivo NIR Fluorescence Imaging in Living Mice

NIR fluorescence imaging were conducted for 2 h after i.v. injection of CDIA@LMWC NP (10 mg k^−1^ g body weight). Fluorescence images of CDIA@LMWC NP were acquired using a live animal imaging system (Tanon ABL X6, China) with excitation at 650 ± 10 nm and emission at 720 ± 10 nm and an acquisition time of 0.1 s. NIR fluorescence intensities of kidneys in living mice were analyzed by the region of interest (ROI) analysis. Mice were euthanized after imaging at different timepoints post‐treatment of cisplatin. Major organs were collected and placed into 4% paraformaldehyde for histological examination.

### PA Imaging

PA imaging was performed using a LAZR Tight TM imaging enclosure coupled with a Vevo LAZR PA imaging system (Fujifilm Visual Sonics) equipped with a MS‐250 linear array transducer (21 MHz, 70% 6 dB two‐way bandwidth, 256 elements) to detect ultrasound (US) signals and a tunable Nd: YAG laser system (OPOTEK, 680−970 nm, 20 Hz repetition rate, 5 ns pulse width, and 50 mJ pulse peak energy) to generate optical pulses. PA spectra were acquired using a transducer at a wavelength of 690 nm and with an acquisition rate of 5 frames/s. US/PA signals were processed and reconstructed in a workstation. The energy supplied by each pulse of the tunable laser was 1.2 mJ cm^−2^, well below the standard set by the American National Standard Institute across the wavelength range. PA imaging in living mice were conducted for 2 h after i.v. injection of CDIA@LMWC NP with excitation at 690 nm.

## Conflict of Interest

The authors declare no conflict of interest.

## Author Contributions

J.T. and H.G. conceived and designed the study. L.S., J.L., and Q.Z. performed the experiments with assistance from X.M. and B.Y.; L.S. synthesized and characterized the materials; H.G. and Q.Z. performed the cell and animal experiments. H.G., J.L., H.X., and R.L. contributed to the discussion. J.T., H.G., L.S., Q.Z., H.X., R.L., and B.Y. analyzed the data and wrote the manuscript. J.T., R.L., and H.X. provided project supervision. All the authors discussed the results and approved the final version of the manuscript.

## Supporting information

Supporting Information

Supplemental Movie 1

Supplemental Movie 2

## Data Availability

The data that support the findings of this study are available from the corresponding author upon reasonable request.

## References

[1] C. Ronco , R. Bellomo , J. A. Kellum , Lancet 2019, 394, 1949. 31777389 10.1016/S0140-6736(19)32563-2

[2] A. J. Kirtane , D. M. Leder , S. S. Waikar , G. M. Chertow , K. K. Ray , D. S. Pinto , D. Karmpaliotis , A. J. Burger , S. A. Murphy , C. P. Cannon , E. Braunwald , C. M. Gibson , J. Am. Coll. Cardiol. 2005, 45, 1781. 15936606 10.1016/j.jacc.2005.02.068

[3] F. Priem , H. Althaus , M. Birnbaum , P. Sinha , H. S. Conradt , K. Jung , Clin. Chem. 1999, 45, 567. 10102918

[4] A. S. Minhas , J. Sharkey , E. A. Randtke , P. Murray , B. Wilm , M. D. Pagel , H. Poptani , Mol. Imaging Biol. 2020, 22, 494. 31529408 10.1007/s11307-019-01429-z PMC7250811

[5] A. J. Shuhendler , K. Pu , L. Cui , J. P. Uetrecht , J. Rao , Nat. Biotechnol. 2014, 32, 373. 24658645 10.1038/nbt.2838 PMC4070437

[6] Xu Zhen , J. Zhang , J. Huang , C. Xie , Q. Miao , K. Pu , Angew. Chem., Int. Ed. 2018, 57, 7804. 10.1002/anie.201803321 29665259

[7] P. Cheng , J. Zhang , J. Huang , Q. Miao , C. Xu , K. Pu , Chem. Sci. 2018, 9, 6340. 30310562 10.1039/c8sc01865k PMC6115726

[8] P. Wei , Q. Wang , T. Yi , Chin. J. Chem. 2022, 40, 1964.

[9] J. Zhang , L. Ning , J. Huang , C. Zhang , K. Pu , Chem. Sci. 2020, 11, 618. 10.1039/c9sc05460j PMC8145638 34123034

[10] Q. Miao , Y. Lyu , D. Ding , K. Pu , Adv. Mater. 2016, 28, 3662. 27000431 10.1002/adma.201505681

[11] L.i‐L.i Li , H.‐L. Ma , G.‐B. Qi , D.i Zhang , F. Yu , Z. Hu , H. Wang , Adv. Mater. 2016, 28, 254. 26568542 10.1002/adma.201503437

[12] K. Pu , A. J. Shuhendler , J. V. Jokerst , J. Mei , S. S. Gambhir , Z. Bao , J. Rao , Nat. Nanotechnol. 2014, 9, 233. 24463363 10.1038/nnano.2013.302 PMC3947658

[13] N. Chen , K. Aleksa , C. Woodland , M. Rieder , G. Koren , Br. J. Pharmacol. Chemother. 2008, 153, 1364. 10.1038/bjp.2008.15 PMC2437918 18278066

[14] Z. Zhou , J. Song , R. Tian , Z. Yang , G. Yu , L. Lin , G. Zhang , W. Fan , F. Zhang , G. Niu , L. Nie , X. Chen , Angew. Chem., Int. Ed. 2017, 129, 6492. 10.1002/anie.201701181 PMC5634745 28470979

[15] J. Hou , H. Wang , Z. Ge , T. Zuo , Q. Chen , X. Liu , S. Mou , C. Fan , Y.i Xie , L. Wang , Nano Lett. 2020, 20, 1447. 31975594 10.1021/acs.nanolett.9b05218

[16] D. Jiang , Z. Ge , H.‐J. Im , C. G. England , D. Ni , J. Hou , L. Zhang , C. J. Kutyreff , Y. Yan , Y. Liu , S. Y. Cho , J. W. Engle , J. Shi , P. Huang , C. Fan , H. Yan , W. Cai , Nat. Biomed. Eng. 2018, 2, 865. 30505626 10.1038/s41551-018-0317-8 PMC6258029

[17] A. P. Blum , J. K. Kammeyer , A. M. Rush , C. E. Callmann , M. E. Hahn , N. C. Gianneschi , J. Am. Chem. Soc. 2015, 137, 2140. 25474531 10.1021/ja510147n PMC4353031

[18] K. Sahin , M. Tuzcu , H. Gencoglu , A. Dogukan , M. Timurkan , N. Sahin , A. Aslan , O. Kucuk , Life Sci. 2010, 87, 240. 20619277 10.1016/j.lfs.2010.06.014

[19] X. Ma , C. Dang , H. Kang , Z. Dai , S. Lin , H. Guan , X. Liu , X. Wang , W. Hui , Int. Immunopharmacol. 2015, 28, 399. 26118633 10.1016/j.intimp.2015.06.020

[20] K. P. Reddy , P. Madhu , P. S. Reddy , Food Chem. Toxicol. 2016, 91, 65. 26925769 10.1016/j.fct.2016.02.017

[21] S. Palipoch , Palipoch. Afr. J. Tradit. Complement Altern. Med. 2013, 10, 88. 24146507 10.4314/ajtcam.v10i4.15 PMC3794397

[22] B. B. Ratliff , W. Abdulmahdi , R. Pawar , M. S. Wolin , Antioxid. Redox Signal 2016, 25, 119. 26906267 10.1089/ars.2016.6665 PMC4948213

[23] M. S. Paller , J. R. Hoidal , T. F. Ferris , J. Clin. Invest. 1984, 74, 1156. 6434591 10.1172/JCI111524 PMC425281

[24] R. Baliga , N. Ueda , P. D. Walker , S. V. Shah , Drug Metab. Rev. 1999, 31, 971. 10575556 10.1081/dmr-100101947

[25] S. N. Heyman , S. Rosen , M. Khamaisi , J.‐M. Idée , C. Rosenberger , Invest Radiol. 2010, 45, 188. 20195159 10.1097/RLI.0b013e3181d2eed8

[26] A. Pisani , E. Riccio , M. Andreucci , T. Faga , M. Ashour , A. Di Nuzzi , A. Mancini , M. Sabbatini , Biomed. Res. Int. 2013, 2013, 868321. 24459673 10.1155/2013/868321 PMC3891610

[27] C. C. Winterbourn , Nat. Chem. Biol. 2018, 4, 278. 10.1038/nchembio.85 18421291

[28] X. Fang , H.‐P. Schuchmann , C. Von Sonntag , J. Chem. Soc. Perkin Trans. 2000, 2, 1391.

[29] N. Aubin , O. Curet , A. Deffois , C. Carter , J. Neurochem. 1998, 71, 1635. 9751197 10.1046/j.1471-4159.1998.71041635.x

[30] L. Yuan , W. Lin , S. Zhao , W. Gao , B. Chen , L. He , S. Zhu , J. Am. Chem. Soc. 2012, 134, 13510. 22816866 10.1021/ja305802v

[31] Q. Miao , D. C. Yeo , C. Wiraja , J. Zhang , X. Ning , C. Xu , K. Pu , Angew. Chem., Int. Ed. 2018, 57, 1256. 10.1002/anie.201710727 29316083

[32] H. Chen , J. Yu , X. Men , J. Zhang , Z. Ding , Y. Jiang , C. Wu , D. T. Chiu , Angew. Chem., Int. Ed. 2021, 60, 2. 10.1002/anie.202100774 PMC8119375 33730372

[33] M. K. Eberhardt , R. Colina , J. Org. Chem. 1988, 53, 1071.

[34] M. Wasil , B. Halliwell , M. Grootveld , C. P. Moorhouse , D. C. S. Hutchison , H. Baum , Biochem. J. 1987, 243, 867. 2821995 10.1042/bj2430867 PMC1147938

[35] A. Yadav , P. C. Mishra , J. Mol. Model 2013, 19, 767. 23053011 10.1007/s00894-012-1601-2

[36] N. Agnihotri , P. C. Mishra , J. Phys. Chem. B 2009, 113, 12096. 19768848 10.1021/jp903604s

[37] H. Yu , L. Cao , F. Li , Q. Wu , Q. Li , S. Wang , Y. Guo , RSC Adv. 2015, 5, 63655.

[38] L. Oziol , P. Faure , C. Vergely , L. Rochette , Y. Artur , P. Chomard , P. Chomard , J. Endocrinol. 2001, 170, 197. 11431152 10.1677/joe.0.1700197

[39] X. Bai , Y. Huang , M. Lu , D. Yang , Angew. Chem., Int. Ed. 2017, 56, 12873. 10.1002/anie.201705873 28845918

[40] M. Pavkovic , B. Riefke , H. Ellinger‐Ziegelbauer , Toxicology 2014, 324, 147. 24880025 10.1016/j.tox.2014.05.005

[41] K. Xu , L. Wang , M. Qiang , L. Wang , P. Li , B.o Tang , Chem. Commun. 2011, 47, 7386. 10.1039/c1cc12473k 21625714

[42] L. V. Johnson , M. L. Walsh , B. J. Bockus , L. B. Chen , J. Cell Biol. 1981, 88, 526. 6783667 10.1083/jcb.88.3.526 PMC2112765

[43] W. Feng , Y. Zhang , Z. Li , S. Zhai , W. Lv , Z. Liu , Anal. Chem. 2019, 91, 15757. 31724390 10.1021/acs.analchem.9b04002

[44] K. Du , A. Ramachandran , H. Jaeschke , Redox Biol. 2016, 10, 148. 27744120 10.1016/j.redox.2016.10.001 PMC5065645

[45] H. Qiao , M. Sun , Z. Su , Y. Xie , M. Chen , L.i Zong , Y. Gao , H. Li , J. Qi , Q. Zhao , X. Gu , Q. Ping , Biomaterials 2014, 35, 7157. 24862442 10.1016/j.biomaterials.2014.04.106

[46] F. P. Ramanery , A. A. P. Mansur , H. S. Mansur , S. M. Carvalho , M. C. Fonseca , Nanoscale Res. Lett. 2016, 11, 187. 27067735 10.1186/s11671-016-1417-6 PMC4828355

[47] A. E. Perez Bay , R. Schreiner , I. Benedicto , M. Paz Marzolo , J. Banfelder , A. M. Weinstein , E. J. Rodriguez‐Boulan , Nat. Commun. 2016, 7, 11550. 27180806 10.1038/ncomms11550 PMC4873671

[48] R. Nielsen , E. I. Christensen , H. Birn , Kidney Int. 2016, 89, 58. 26759048 10.1016/j.kint.2015.11.007

[49] Y. Tong , X. Huang , M.i Lu , B.o‐Y. Yu , J. Tian , Anal. Chem. 2018, 90, 3556. 29443497 10.1021/acs.analchem.7b05454

[50] M. E. M. Dolman , S. Harmsen , G. Storm , W. E. Hennink , R. J. Kok , Adv. Drug Delivery Rev. 2010, 62, 1344. 10.1016/j.addr.2010.07.011 20719242

[51] Y. Luo , L. Huang , Y.e Yang , X. Zhuang , S. Hu , H. Ju , B.o‐Y. Yu , J. Tian , Theranostics 2017, 7, 1245. 28435462 10.7150/thno.18187 PMC5399590

[52] Z.‐X. Yuan , Z.‐R. Zhang , D.i Zhu , X. Sun , T. Gong , J. Liu , C.‐T. Luan , Mol. Pharmaceutics 2009, 6, 305. 10.1021/mp800078a 19035784

[53] F. D. Debelle , J.‐L. Vanherweghem , J. L. Nortier , Kidney Int. 2008, 74, 158. 18418355 10.1038/ki.2008.129

[54] A. Bouslimi , Z. Ouannes , E. E.l Golli , C. Bouaziz , W. Hassen , H. Bacha , Toxicol. Mech. Methods 2008, 18, 341. 20020900 10.1080/15376510701556682

[55] X. Xu , N.i Zheng , Z. Chen , W. Huang , T. Liang , H. Kuang , Gene 2016, 591, 411. 27317894 10.1016/j.gene.2016.06.032

[56] Y.‐Z. Zheng , G. Deng , Q. Liang , D.a‐F.u Chen , R. Guo , R.‐C. Lai , Sci. Rep. 2017, 7, 7543. 28790397 10.1038/s41598-017-08024-8 PMC5548903

[57] X.u Zhang , H. Huang , T. Yang , Y. Ye , J. Shan , Z. Yin , L. Luo , Injury, Int. J. Care Injured 2010, 41, 746. 10.1016/j.injury.2010.02.029 20227691

[58] Q. Weng , H. Sun , C. Fang , F. Xia , H. Liao , J. Lee , J. Wang , A.n Xie , J. Ren , X. Guo , F. Li , B.o Yang , D. Ling , Nat. Commun. 2021, 12, 1436. 33664241 10.1038/s41467-021-21714-2 PMC7933428

[59] Y. Chen , P. Pei , Z. Lei , X. Zhang , D. Yin , F. Zhang , Angew. Chem., Int. Ed. 2021, 60, 15809. 10.1002/anie.202103071 33876514

[60] C.‐F. Luo , B. Cai , N. Hou , M.u Yuan , S.‐M. Liu , H. Ji , L.‐G. Xiong , W. Xiong , J.‐D. Luo , M.‐S. Chen , Arch. Toxicol. 2012, 86, 1681. 22648071 10.1007/s00204-012-0874-7

[61] M. T. Do , H. G. Kim , J. H. Choi , H. G. Jeong , Free Radical Bio. Med. 2014, 74, 21. 24970682 10.1016/j.freeradbiomed.2014.06.010

[62] S. R. Kulkarni , A. C. Donepudi , J. Xu , W. Wei , Q. C. Cheng , M. V. Driscoll , D. A. Johnson , J. A. Johnson , X. Li , A. L. Slitt , Antioxid. Redox Signaling 2014, 20, 15. 10.1089/ars.2012.5082 PMC3880903 23725046

[63] Y. Zhang , X. Tao , L. Yin , L. Xu , Y. Xu , Y. Qi , X.u Han , S. Song , Y. Zhao , Y. Lin , K. Liu , J. Peng , Br. J. Pharmacol. Chemother. 2017, 174, 2512. 10.1111/bph.13862 PMC5513863 28514495

[64] G. M. Denicola , F. A. Karreth , T. J. Humpton , A. Gopinathan , C. Wei , K. Frese , D. Mangal , K. H. Yu , C. J. Yeo , E. S. Calhoun , F. Scrimieri , J. M. Winter , R. H. Hruban , C. Iacobuzio‐Donahue , S. E. Kern , I. A. Blair , D. A. Tuveson , Nature 2011, 475, 106. 21734707 10.1038/nature10189 PMC3404470

[65] F. Zhao , T. Wu , A. Lau , T. Jiang , Z. Huang , X.‐J. Wang , W. Chen , P. K. Wong , D. D. Zhang , Free Radical Bio. Med. 2009, 47, 867. 19573594 10.1016/j.freeradbiomed.2009.06.029 PMC2748111

[66] C. M. Clements , R. S. Mcnally , B. J. Conti , T. W. Mak , J. P.‐Y. Ting , Proc. Natl. Acad. Sci. USA 2006, 103, 15091. 17015834 10.1073/pnas.0607260103 PMC1586179

[67] A. Cuadrado , A. I. Rojo , G. Wells , J. D. Hayes , S. P. Cousin , W. L. Rumsey , O. C. Attucks , S. Franklin , A.‐L. Levonen , T. W. Kensler , A. T. Dinkova‐Kostova , Nat. Rev. Drug Discovery 2019, 18, 295. 30610225 10.1038/s41573-018-0008-x

[68] J. N. Guzman , J. Sanchez‐Padilla , D. Wokosin , J. Kondapalli , E. Ilijic , P. T. Schumacker , D. J. Surmeier , Nature 2010, 468, 696. 21068725 10.1038/nature09536 PMC4465557

