# Supplementary material for: Illumination of Hydroxyl Radical in Kidney Injury and High‐Throughput Screening of Natural Protectants Using a Fluorescent/Photoacoustic Probe
Source: Adv Sci (Weinh). 2023 Oct 23;10(33):2303926. doi: 10.1002/advs.202303926 (PMC10667829; doi:10.1002/advs.202303926)
Supplement: Supplementary file 1 — Supporting Information [file ADVS-10-2303926-s001.pdf]

ADVANCED SCIENCE
Open Access

Supporting Information

for *Adv. Sci.*, DOI 10.1002/advs.202303926

Illumination of Hydroxyl Radical in Kidney Injury and High-Throughput Screening of Natural Protectants Using a Fluorescent/Photoacoustic Probe

Han Gao, Lei Sun, Jiwei Li, Qilin Zhou, Haijun Xu, Xiao-Nan Ma, Renshi Li*, Bo-Yang Yu and Jiangwei Tian**

Supporting Information

Illumination of Hydroxyl Radical in Kidney Injury and High-Throughput Screening of Natural Protectants Using A Fluorescent/Photoacoustic Probe

Han Gao, Lei Sun, Jiwei Li, Qilin Zhou, Haijun Xu, Xiao-Nan Ma, Renshi Li,* Bo-Yang Yu, and Jiangwei Tian**

H. Gao, J. Li, Q. Zhou, X.-N. Ma, R. Li, B.-Y. Yu, J. Tian
State Key Laboratory of Natural Medicines, Jiangsu Key Laboratory of TCM Evaluation and Translational Research, Cellular and Molecular Biology Center,
School of Traditional Chinese Pharmacy, China Pharmaceutical University, Nanjing 211198, P.R. China
E-mail: jwtian@cpu.edu.cn, li-renshi@cpu.edu.cn

L. Sun, H. Xu
Jiangsu Co-innovation Center of Efficient Processing and Utilization of Forest Resources, Key Laboratory of Forestry Genetics & Biotechnology of Ministry of Education, Jiangsu Provincial Key Lab for the Chemistry and Utilization of Agroforest Biomass,
College of Chemical Engineering, Nanjing Forestry University, Nanjing 210037, P.R. China
E-mail: xuhaijun@njfu.edu.cn

H. Xu
School of Chemistry and Chemical Engineering, Henan Normal University, Xinxiang, 453002, P.R. China

Table of Contents

Experimental Section 3

Chemical Synthesis and Characterization 13

Supporting Figures and Tables 20

References 65

Experimental Section

Materials and Reagents. All the oligonucleotides used in this study were custom-synthesized by Sangon Biological Engineering Technology & Services Co., Ltd. (Shanghai, China). Low molecular weight chitosan (LMWC > 90% deacetylation) with number-average molecular weight of 5000 was purchased from Sigma-Aldrich (St. Louis, MO, USA). Sodium tripolyphosphate (TPP, purity > 98%) was purchased from Macklin Biochemical Co., Ltd (Shanghai, China). Sulfo-Cyanine7 carboxylic acid (Cy7) was obtained from Xi'an ruixi Biological Technology Co., Ltd (Xi'an, China). 3-(4,5-dimethylthiazol)-2,5-diphenyltetrazolium-bromide (MTT), dimethyl sulphoxide (DMSO), Dulbecco's Modified Eagle Medium (DMEM), Dulbecco's Modified Eagle Medium/Nutrient Mixture F-12 (DMEM/F12, a mixture of DMEM and Ham's F-12 Nutrient Mixture (F12)), fetal bovine serum (FBS) and phosphate buffer solutions (PBS, 137 mM NaCl, 2.7 mM KCl, 4.3 mM Na_2HPO_4, 1.4 mM KH_2PO4, pH 7.4) were purchased from Gibco (Gaithersburg, USA). Fluorescein isothiocyanate (FITC, purity > 95%), lipopolysaccharide (LPS), vitamin C (VC), citrinin (CTN), aristolochic acid I (AAI), cis-diammineplatinum dichloride (CDDP), quercetin (Que), puerarin (PUE) and other reagents were purchased from Sigma-Aldrich Reagent, Ltd. (Shanghai, China). Trizol RNA isolation reagent, LysoTracker® Green, MitoTracker® Green, and Hoechst 33342 were purchased from Invitrogen Company (Carlsbad, CA, USA). A stock solution (1 mM) of CDIA was prepared by dissolving an appropriate amount of CDIA in chromatogram class DMSO. The HiScript II Q RT SuperMix for qPCR, HiScript II One Step qRT-PCR SYBR Green Kit, 180 kDa Prestained Protein Marker, One-Step PAGE Gel Fast Preparation Kit (10%) and enhanced chemiluminescence (ECL) detection kit were purchased from Vazyme Biotech Co., Ltd. (Nanjing, China). Enhanced BCA Protein Assay Kit, Total Superoxide

Dismutase Assay Kit with WST-8 and Lipid Peroxidation MDA Assay Kit, ER Tracker Green and Golgi Tracker Green were purchased from Beyotime Biotech Co., Ltd. (Shanghai, China). Serum creatinine (sCr) and blood urea nitrogen (BUN) assay kits were obtained from Nanjing Jiancheng Biotech Co., Ltd (Nanjing, China). All the organic solvents were of analytical grade or the best grade available. All aqueous solutions were prepared in ultra-pure water obtained from a Milli-Q water purification system (18 M Ω cm).

Apparatus. The compound was characterized by ^{1}H NMR and mass spectra. ^{1}H NMR spectrum was measured on a Bruker DRX600 spectrometer and referenced to the residual proton signals of the solvent. Absorption spectra were recorded on an UV-2550 UV-Vis-NIR spectrophotometer (Shimadzu Company, Japan). Fluorescence spectra were measured on an F-7000 spectrofluorometer (Hitachi, Japan). HPLC spectrum was measured with a 1260 Infinity II spectrometer (Agilent Technologies, USA). Mass spectrum was measured with a InfinityLab LC/MSD Series spectrometer (Agilent Technologies, USA). All pH measurements were carried out with a Sartorius PB-10 (Sartorius scientific instruments, Beijing, China) containing a composite glass electrode. The morphology of the nanoprobe was characterized at a HITACHI HT7700 transmission electron microscope (TEM) operated at 120 kV. The particle size and size distribution were obtained by dynamic light scattering (DLS) at 25 °C by means of a 90 Plus/BI-MAS equipment (Brookhaven, USA). Zeta potential measurement was performed at 25 °C on a Malvern Zetasizer-Nano Z instrument. MTT assay was performed using a microplate reader (Infinite M200 Pro, Tecan). Confocal fluorescence imaging of cells was performed on a confocal laser scanning microscope (LSM800, Zeiss, Germany) and processed using the ZEN imaging software. Flow cytometric assay was performed using MACSQuant Analyzer 10 (Miltenyi Biotec, Germany). PCR reactions were performed on the Quant Studio 6 Flex Real-Time PCR System (Applied Biosystems, Carlsbad, California, USA). In vivo fluorescence imaging experiments were performed on a live animal imaging system (Tanon ABL X6, China). The hematoxylin and eosin (H&E) staining images were acquired on a digital pathology slice scanner using NanoZoomer 2.0 RS (Hamamatsu, China). The photoacoustic (PA) signal of diseases areas were measured by Visualsonic Vevo 2100 LAZER system.

Measurement of Fluorescence Quantum Yields. The fluorescence quantum yields were measured using Cy7 in DMSO ($\Phi_F = 0.3$) as the standard and calculated with the following equation,

$$\Phi_{F(x)} = \Phi_{F(std)} \left(\frac{A_{std}}{A_x}\right) \left(\frac{I_x}{I_{std}}\right) \left(\frac{\eta_x}{\eta_{std}}\right)^2$$

where subscript x designates CDIA, subscript std designates Cy7, *A* stands for the absorbance at

excitation wavelength, *I* stands for the integrated fluorescence intensity, and η stands for the refractive index of the solvent in the measurement.

Preparation of Analyte Solutions. •OH: Hydroxyl radical was generated by Fenton reaction. To generate •OH, various amounts of Fenton reagent (Fe^{2+}/H_2O_2 = 1/6, molar ratio) were added into DMF/0.1 mM H_2SO_4 = 1/1 (v/v).[1] **$^1O_2$:** Singlet oxygen was generated in situ by addition of the H_2O_2 stock into a solution containing 10 eq of HClO. **$ONOO^-$:** A mixture of sodium nitrite (0.6 M) and hydrogen peroxide (0.7 M) was acidified with hydrochloric acid (0.6 M), and sodium hydroxide (1.5 M) was added within 1–2 s to make the solution alkaline. The excess hydrogen peroxide was removed by passing the solution through a short column of manganese dioxide. The peroxynitrite concentration was estimated by using an extinction coefficient of 1670 M^{-1}•cm^{-1} at 302 nm. C_{ONOO-} = Abs_{302} nm /1.67 (mM).[2] **NO:** Nitric oxide was used from a stock solution prepared by sodium nitroprusside. **ClO^-:** hypochlorite was delivered from commercial aqueous solution. **TBHP:** *Tert*-Butyl hydroperoxide was delivered from commercial aqueous solution. **H_2O_2:** Hydrogen peroxide was delivered from commercial aqueous solution. **$O_2^{\bullet-}$:** Superoxide solution was prepared by adding KO_2 to dry dimethylsulfoxide and stirring vigorously for 10 min. **T_4:** Freshly prepared thyroxine solution (5 mM) was added directly. **T_3:** Freshly prepared triiodothyronine solution (5 mM) was added directly. **TEMPO:** Freshly prepared stock solution of 2,2,6,6-tetramethyl-1-piperidinyloxy (100 mM) was added directly. **Thiourea:** Freshly prepared thiourea (100 mM) was added directly. **GSH:** Glutathione (100 mM) was added directly. **NAC:** *N*-Acetylcysteine (100 mM) was added directly.

Synthesis of FITC-LMWC. 5 mg of FITC was dissolved in 500 μL DMSO and added to 50 mg LMWC immersed in 5 mL deionized water. After 16 h of incubation at room temperature in the dark, the mixture was dialyzed (MWCO 1000) for 48 h at room temperature in the dark, followed by lyophilization.

Synthesis of FITC-LMWC NP. FITC-LMWC NP was synthesized by using an ionic crosslinking method.[3] In brief, 5 mg FITC-LMWC was dissolved in 5 mL 1% acetic acid solution, 2 mL 1.25 mg/mL TPP solution was added dropwise into FITC-LMWC solution while stirring at 1000 rpm. The conjugation reaction was maintained for 30 min, and the product was then purified by successive dialysis (MWCO 10000) against deionized water for 48 h. Then the product solution was adjusted to 7.4. The final product was freeze-dried and kept in desiccators for use. The amount of FITC conjugated on FITC-LMWC NP was determined by absorption at 490 nm according to a previous method.[4]

Fluorescence Measurement of FITC-LMWC NP. FITC-LMWC NP was diluted with deionized water to obtain final concentrations: 0, 1, 2, 3, 4, 5, 6, 7, 8, 9, 10 μg/mL. The solution was excited at 490 nm and the emission wavelengths were collected from 500 to 700 nm. The fluorescence spectra was measured on Cary Eclipse Fluorescence Spectrophotometer and both excitation and emission slits were set to 5.0 nm. The standard curve of fluorescence intensity was recorded at $\lambda_{ex}/\lambda_{em}$ = 490/518 nm using a spectrofluorometer.

Characterization of Nanoparticles. TEM measurement was performed with a JEOL JEM-200CX TEM at an accelerating voltage of 200 kV. The samples were prepared by dropping 10 μL of 10 μM CDIA onto copper grids, and dried for 3 min. Then the residual solution was blotted off using filter paper. Dynamic light scattering and zeta potential measurements of LMWC NP, FITC-LMWC NP and CDIA@LMWC NP were performed at 25 °C on Mastersizer 2000 particle size analyzer and Malvern Zeta sizer-Nano Z instrument. The samples were dispersed in double distilled water and the pH value of the solution was adjusted to 7.4 before the analysis.

Determination of Encapsulation Efficiency and Loading Content. To determine the encapsulation efficiency (EE) of CDIA@LMWC NP, a dialysis method has been used.[5] 10 mL freshly prepared CDIA@LMWC NP containing 7.14 mg CDIA was placed in a dialysis bag (MWCO 5000), immersed in 90 mL phosphate buffer solution and shaken at 500 rpm using a magnetic stirrer. 5 mL dialysis sample solution was collected and the concentration of probe was measured by the UV-Vis-NIR absorption method. The EE of CDIA was calculated as: EE (%) = $(W_1-W_2)/W_1\times100$, where W_1 and W_2 were the weights of added CDIA and unloaded CDIA, respectively. The loading content (LC) of CDIA was calculated as: LC (%) = $(W_1-W_2)/W_t\times100$, where W_1 and W_2 were the weights of added CDIA and unloaded CDIA, respectively, and W_t was the weight of LMWC NP. The determinations were performed at least five times for each calculation.

In Vitro Release of CDIA. The in vitro release profile of CDIA release from CDIA@LMWC NP was carried out at pH 7.4, 6.8, 5.0, and 4.0 using a dialysis method with free CDIA solution at the same concentration as a control. 1 mL of 1 mg/mL CDIA@LMWC NP was placed in a dialysis bag (MWCO 5000), immersed in 50 mL PBS and incubated at 37 °C in a shaking platform at 500 rpm. At predetermined intervals, 1 mL dissolution sample was collected and replaced with the same volume of release medium. The concentration of CDIA released from CDIA@LMWC NP was measured by the UV absorption method as mentioned.

General Procedure for UV-Vis-NIR Absorption Spectra Measurement. All the UV-Vis-NIR absorption spectra measurements were performed in 10 mM phosphate buffer (pH 7.4). To build a standard curve of CDIA content, different final concentrations (0, 1, 2.5, 5, 7.5, 10, 15, 20 μM) of CDIA was added into PBS buffer. The absorbance at 650 nm was measured using a spectrophotometer. To build a standard curve of FITC content, different final concentrations (0, 3, 6, 9, 12, 15 μg/mL) of FITC was added into PBS buffer. The absorbance at 490 nm was measured using a spectrophotometer. For •OH detection, 10 μM CDIA was added into different concentration of freshly prepared •OH (0 – 50 μM, prepared by Fenton reaction). After rapidly mixing the solution and incubating at 37 °C for 30 min in a thermostat, a 3 mL portion of the reaction solution was transferred to a 10 × 10 mm quartz cell for *in vitro* detection.

General Procedure for Fluorescence Spectra Measurement. CDIA was dissolved in DMSO to make a 5 mM stock solution, which was diluted to the required concentration of testing solution for measurement. Fluorescence spectra was recorded with $\lambda_{ex}/_{em}$ = 650/720 nm and both excitation and emission slit widths were set to 5.0 nm. Meanwhile, a blank solution without •OH (control) was prepared and measured under the same condition for comparison. For •OH response detection, 50 μL 200 μM CDIA was dissolved in 450 μL PBS, and the solution was incubated with 500 μL Fenton reagent at various concentrations (0−200 μM) at 37 °C for 30 min. The fluorescence spectra of CDIA were recorded using a spectrofluorometer with the excitation wavelength at 650 nm and the emission wavelengths from 675 to 875 nm.

For CDIA selectivity detection, different kinds of analyte solutions were incubated with 10 μM CDIA solution at 37 °C for 30 min, respectively, and then recorded using a spectrofluorometer. All the experiments were repeated at least five times.

For fluorescence kinetics measurement, 10 μM CDIA was added into Fenton reagent at various concentrations (0, 10, 30, 50, 100 μM) at 37 °C, respectively. The fluorescence intensity was examined with increasing time (0, 0.5, 1, 2.5, 5, 7.5, 10, 12.5, 15, 17.5, 20, 22.5, 25, 27.5, 30 minutes). Then the fluorescence was excited at 650 nm and measured at 720 nm. All the experiments were repeated at least five times.

Cell Culture. Alpha mouse liver 12 AML-12 cells, rat heart muscle H9c2 cells, human umbilical vein endothelial HUVEC cells, human mammary cancer MCF-7 cells, human proximal renal tubular epithelial HK-2 cells were obtained from KeyGEN Biotech Co., Ltd (Nanjing, China). AML-12, H9c2, HUVEC and MCF-7 cells were routinely grown in Dulbecco's modified Eagle's medium (DMEM) containing 10% FBS, 100 μg/mL streptomycin, and 100 U/mL penicillin. HK-2 cells were cultured in

Dulbecco's modified Eagle's medium/Ham's nutrient mixture F12 (DMEM/F12) supplemented with 10% FBS, 100 μg/mL streptomycin, and 100 U/mL penicillin. The above cells were grown in a 100% humidified atmosphere containing 5% CO_2 at 37 °C. The medium was replenished every day and the cells were subcultured after reaching confluence.

Cytotoxicity Assay. To investigate the cytotoxicity of CDIA, standard MTT tests were performed in AML-12, H9c2, HUVEC, MCF-7 and HK-2 cells. HepG2 cells growing in log phase were seeded into 96-well plates at a seeding density of 1×10^4 cells per well in 200 μL complete medium and allowed to attach for 24 h. After rinsing with PBS, HepG2 cells were incubated with 200 μL culture media containing serial concentrations of CDIA (0, 1, 2, 5, 10, 15, 20 μg/mL) for 24 h. Then 20 μL of MTT (5 mg/mL in PBS) was added to each well and followed by incubated for 4 h under the respective conditions. After 4 h, the supernatants containing unreacted MTT were discarded carefully, and 150 μL DMSO was added to each well to dissolve the produced blue formazan. The absorbance was recorded at 570 nm using a microplate reader after 10 min of shaking. Cell viability was determined by the following formula: Cell viability (%) = 100 × (OD_1-OD_2)/(OD_3-OD_2), where OD_1, OD_2, and OD_3 were OD value of treatment group, OD value of blank group, and OD value of control group, respectively. All the experiments were repeated at least five times. The similar cytotoxicity assay was applied to nanoparticles (LMWC NP, FITC-LMWC NP and CDIA@LMWC NP) of concentrations from 0 to 2 mg/mL to HK-2 AML-12, H9c2, MCF-7 and HK-2 cells, respectively.

To evaluate the cytotoxicity of CDDP, AAI, and CTN to HK-2 cells, HK-2 cells were dispersed in 96-well microtiter plates at 1×10^4 cells per well in a total volume of 200 μL. The plates were maintained in a humidified atmosphere with 5% CO_2 at 37 °C for 24 h. After the original medium has been discarded, all the drugs with 0.1% DMSO at final concentrations of 1 ~ 1000 μM was diluted in fresh medium, then the medium was added to the HK-2 cells respectively, and incubated for 24 h. The cell viability test methods were the same as above.

Confocal Fluorescence Imaging for Determination of Optimal Incubation Conditions. HK-2 cells were seed into 35-mm confocal dishes (Glass Bottom Dish) at a density of 1×10^4 per dish and incubated in complete medium at 37 °C. After 24 h, the medium was replaced with fresh serum-free culture medium containing FITC-LMWC NP (0.5 μg/mL, 1 μg/mL, 2 μg/mL, 5 μg/mL, 10 μg/mL, 20 μg/mL) and incubated at 37 °C for 10 min, 40 min, 100 min and 130 min, respectively. Cell imaging was then carried out after washing cells three times with PBS buffer (pH 7.4) to remove any FITC-LMWC NP on the surface of cells. The fluorescence of cells was visualized using LSM800 with a Plan-Apochromat 63×/1.40 Oil DIC M27 lens (Zeiss) at stationary parameters including the laser

intensity and exposure time. The fluorescence signal of cells incubated with FITC-LMWC NP was excited at 488 nm with an argon ion laser and the emission was collected from 500 to 650 nm.

Cellular Uptake Assay. The cellular uptake assay of FITC-LMWC NP was observed using confocal fluorescence imaging, HK-2 AML-12, H9c2, HUVEC, MCF-7 and HK-2 cells were seeded into 35-mm confocal dishes (Glass Bottom Dish) at a density of 1×10^4 per dish and incubated in complete medium for 24 h at 37 °C. Then the above cells were exposed to 20 μg/mL FITC-LMWC NP diluted with serum-free medium for 100 min at 37 °C, respectively. After incubation, the cells were stained with 1.0 μM Hoechst 33342 for 25 min, rinsed three times with PBS (pH 7.4) to perform fluorescence imaging with a LSM800 at stationary parameters including the laser intensity, exposure time, and objective lens. Hoechst 33342 was excited with a violet 405 nm laser diode and the emission was collected from 420 to 500 nm. FITC was excited at 488 nm with an argon ion laser and the emission was collected from 500 to 650 nm. All images were digitized and analyzed by a ZEN imaging software. For competitive inhibition assay, HK-2 cells were seeded into 35-mm confocal dishes (Glass Bottom Dish) at a density of 1×10^4 per dish and incubated with 10 μg/mL FITC-LMWC NP diluted into serum-free medium in the absence or presence of 1 μM EDTA, 50 μg/mL LMWC, or 50 μg/mL glucosamine for 2 h at 37 °C. Once uptake was terminated, the cells were rinsed three times with PBS (pH 7.4) to remove extracellular FITC-LMWC NP. The fluorescence of cells was visualized using LSM800 and fluorescence signal of cells incubated with FITC-LMWC NP was excited at 488 nm with an argon ion laser and the emission was collected from 500 to 650 nm. All images were analyzed by a ImageJ software.

Flow Cytometric Assay. For flow cytometric assay, cells were seeded at a density of 2×10^5 cells per well in 6-well plates and incubated with different treatments at 37 °C for different times to produce normoxic or hypoxic cells. After a certain time, the medium was replaced with cell culture medium containing CDIA (10 μM) or CDIA@LMWC NP (20 μg/mL). After incubation, the different treatment groups of cells were washed three times with PBS solution, trypsinized, harvested, rinsed with PBS and resuspended in 500 μL PBS medium, and subjected to flow cytometric assay using MACSQuant Analyzer 10. The data were analyzed with FCS Express V3.

Cellular ROS Detection. For detection of •OH production, HK-2 cells were dispersed in 96-well microtiter plates at 1×10^4 cells per well in a total volume of 200 μL. The plates were maintained in a humidified atmosphere with 5% CO_2 at 37 °C for 24 h. After rinsing with PBS three times, HK-2 cells were incubated with 200 μL of culture media containing serial concentrations (0, 5, 10, 20, 40, 60, 80,

100 μM) of CDDP, AAI and CTN for 24 h at 37 °C, respectively. After incubation, the HK-2 cells were treated with 10 μM commercial ROS probe dichlorodihydrofluorescein diacetate (H_2DCFDA) for 30 min, and then the fluorescence intensity was measured using a spectrofluorometer at $\lambda_{ex}/\lambda_{em}$ = 492/524 nm.

RNA Extraction and qRT-PCR. To study the protective effect of Pue on the HK-2 cells, 75 μM CDDP and 25 μM Pue or 50 μM Pue were administered, respectively. HK-2 cells were pretreated with 25 μM Pue or 50 μM Pue for 12 h before 75 μM CDDP administration. The total RNA was extracted from the HK-2 cells of each group to detect Sirt11, HO-1, Nrf2, Keap,1, and DJ-1 mRNA levels.
Total RNA was isolated from HK-2 cells using Trizol reagent, and cDNA was prepared using a HiScript II Q RT SuperMix for qPCR. Quantitative RT-PCR was carried out using HiScript II One Step qRT-PCR SYBR Green Kit with 20 μL reaction mixtures. Primer names and sequences for qRT-PCR are listed in Supplementary Table S6. PCR reactions were performed on the Quant Studio 6 Flex Real-Time PCR System with the following program: step 1, 95 °C for 3 min to activate the Taq polymerase; step 2, 95 °C for 3 s to denaturize DNA; step 3, 60 °C for 31 s for annealing/extension (39 cycles for steps 2 and 3). The relative mRNA levels were quantified by the $2^{-\Delta\Delta Ct}$ method and all data were normalized to GAPDH (the internal control).

Western Blot Analysis. Immunoblotting analysis of proteins in HK-2 cells was performed by homogenizing frozen cells in the lysis buffer containing 50 mM Tris-HCl, 150 mM sodium chloride, 2 mM ethylene diamine tetraacetic acid (EDTA), 2 mM ethylene glycol tetraacetic acid (EGTA), 1% Triton-X 100, and a protease inhibitor (pH 7.4). The supernatants of the mixture were obtained by centrifugation at 4 °C (30 min, 16,000 × g), and their total protein concentrations were further determined using the Enhanced BCA Protein Assay Kit. Protein extracts were separated on sodium dodecyl sulfatepolyacrylamide gel electrophoresis (SDS-PAGE, 8-12% gels) and blotted onto PVDF membranes. After blocking with 5% fatfree milk, the membranes were incubated at 4 °C overnight with the following primary antibodies: anti-Nrf2 (1:1000, A21176), anti-Keap1 (1:1000, A1820), anti-HO-1 (1:1000, A19062), anti-DJ-1 (1:1000, A19097), anti-Sirt1 (1:1000, A19667), and anti-GAPDH (1:1000, A19056) from ABclonal Technology (WuHan, China). The bound antibodies were detected using horseradish peroxidase (HRP)-conjugated IgG (ABclonal) and visualized with enhanced chemiluminescence (ECL) detection reagents. GAPDH was used as a loading control.

MDA and SOD Assays. To assess the OS level in HK-2 cells, the levels of SOD and MDA were tested with commercially available kits (Beyotime Biotechnology, China). Briefly, HK-2 cells were

homogenized in ice-cold 0.1 M phosphate buffer (pH 7.4), then the homogenates were filtered and centrifuged using a refrigerated centrifuge at 4 °C (20 min, 16,000 × g). The obtained supernatants were used to determine the SOD enzyme activity and the lipid peroxidation level by measuring MDA content. The SOD enzyme activity was expressed as a unit of activity per milligram of protein and the MDA content was expressed as micromole per gram of protein.

Animal Model. All animal treatments were approved by the Animal Ethics Committee of China Pharmaceutical University (protocol no. 2020-07-008). Animal testing and research conformed to all relevant ethical regulations. ICR-Swiss male mice (20-25 g), 5-6 weeks of age, were purchased from the Laboratory Animal Center and Institute of Comparative Medicine at Yangzhou University (certificate no. 202012014). The animals were bred in stainless steel metabolic cages with free access to food and double distilled water under standard conditions of humidity (50 ± 10%), temperature (25 ± 2 °C) and light (12 h light/12 h dark cycle). All animal study protocols were designed in according to guidelines set by the National Institute of Health Guide for the Care and Use of Laboratory Animals, and approved by the Animal Ethical Experimentation Committee of China Pharmaceutical University. The mice were randomly assigned to 4 experimental groups (group I: mice were treat once with CDDP at a dosage of 18 mg/kg; group II: mice were treated twice with CDDP at a dosage of 9 mg/kg; group III: mice were treated thrice with CDDP at a dosage of 6 mg/kg; group IV: mice were treated with CDDP at a dosage of 3 mg/kg for six times.). The control groups were treated with PBS (0.2 ml) or NAC (10 mg kg^{-1} day^{-1} during the study, i.p. injection) or quercetin (Que, 10 mg kg^{-1} day^{-1} during the study, i.p. injection) 3 days prior to CDDP administration.[6] NIR fluorescence and PA imaging were performed for 2 h after i.v. injection of 10 mg/kg CDIA@LMWC NP.

Hemolysis Assay. Hemolytic acitvity of CDIA was carried out according to the previous protocol.[7] Fresh mouse blood was diluted by physiological saline and centrifuged to isolate red blood cells (RBCs) from serum. Then RBCs were incubated with physiological saline (negative control), triton X-100 (10 g/L, positive control), or CDIA at concentrations of 1, 2.5, 5, 10, 20, and 40 μM at 37 °C for 2 h. After incubation, RBCs were centrifuged at 3000 r.p.m. for 10 min and the supernatant of each sample was collected and transferred to a 96-well plate. The absorbance of hemoglobin at 540 nm was measured by using a microplate reader. Because CDIA had intrinsic absorption at 540 nm, the absorption of sample group (A_{sample}) was deducted by the absoption of CDIA at 540 nm at the same concentration. The hemolysis ratio of RBCs was calculated as follows: Hemolysis (%) = ($A_{\text{sample}} - A_{\text{negative}}$)/ ($A_{\text{positive}} - A_{\text{negative}}$) ×100%, where A_{sample}, A_{negative}, A_{positive} were denoted as the absortion of samples (after deduction by the intrinsic absorption of CDIA), negative and positive control, respectively. The same

operation was used for the hemolysis test of CDIA@LMWC NP.

Histology. The organs were fixed with 4% paraformaldehyde (PFA), dehydrated in ethanol solution, and embedded in paraffin prior to 10 μm section. Histology samples were stained by hematoxylin and eosin under standard protocols. Images were acquired using a slide scanner (NanoZoomer 2.0 RS, Hamamatsu).

Pharmacokinetic Studies. Mice were anesthetized with isoflurane for the entire duration of the experiment. The end of the tail was cut for blood extraction. Blood was sampled in heparinized capillary tubes as a reference before injection. Mice were i.v. injected with CDIA@LMWC NP (10 mg/kg body weight) and blood was sampled at 1, 5, 10, 15, 25, 35, 50, 75, 100, 150, 200- and 300-min post-injection. Collected blood samples were stored in an ice box to prevent clotting before centrifugation at 4500 r.p.m for 15 min. CDIA@LMWC NP in the blood was quantified using HPLC and plotted as a function of time to calculate elimination half-life value ($t_{1/2\beta}$).

Blood Sample Analysis. The blood samples were collected at different time post treatment in the heparin capillaries through orbital venous of mice, and centrifuge for 20 min (4 °C, 2000 rpm). The supernatant serum were collected and stored at –80 °C. BUN and sCr concentration were determined using commercial kits according to the manufacturer’s protocol (5 mice were analyzed for each sample).

Data Analysis. Results were expressed as the mean ± standard deviation unless stated otherwise. Cell imaging data was analyzed and quantified with Image J and ZEN software. Statistical comparisons between groups were determined by t-test and more than three groups were determined by one-way ANOVA with multiple comparisons test. For all tests, $P < 0.05$ was considered statistically significant. All statistical calculations were performed using GraphPad Prism.

Chemical Synthesis and Characterization

Scheme 1. Synthesis of **1a**

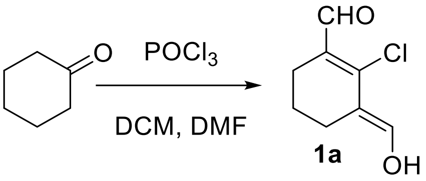

To a chilled solution of dimethylformamide (10 mL, 129 mmol, 5.4 eq.) in 10 mL CH_2Cl_2 under N_2 atmosphere, $POCl_3$ (10 ml, 107 mmol, 4.5 eq.) in 10 mL CH_2Cl_2 were added dropwise under an ice bath. After 15 min, cyclohexanone (2.5 mL, 24 mmol, 1 eq.) was added, and the resulting mixture was refluxed with vigorous stirring for 8 h at 80 °C, then poured into ice-cold water, and kept it for 2 hours to obtain **1a** as a yellow solid (3.8 g, 91%), and the obtained product could be used for next step directly without further purification. The product was stored in argon at -20 °C.

Scheme 2. Synthesis of **1b**

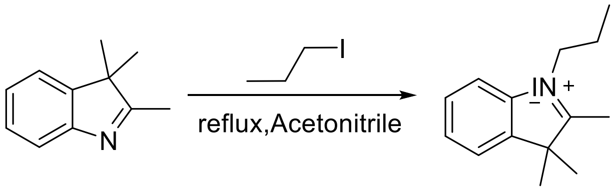

To a solution of 2,3,3-trimethyl-3H-indole (2 g, 12.5 mmol, 1 eq.) in 50 mL acetonitrile, 1-iodopropane (8.3 mL, 62.5 mmol, 5 eq.) was added, and the mixture was heated to reflux with continuous stirring for 14 h. The mixture was dried in vacuum and washed by Et_2O and CH_2Cl_2. The resulting solid was recrystallized with acetone to obtain **1b** a light red solid (3.3 g, 80%). ^1H NMR (600 MHz, $CDCl_3$) δ = 7.53-7.52(m, 1H), 7.40 (d, J = 4.2 Hz, 1H),7.32-7.27 (m, 2H), 4.33 (t, J = 7.2 Hz, 2H), 2.82 (s, 3H), 1.76 (dd, J = 14.4, 7.2 Hz, 2H), 1.37 (s, 6H), 0.79 (t, J = 7.2 Hz, 3H).

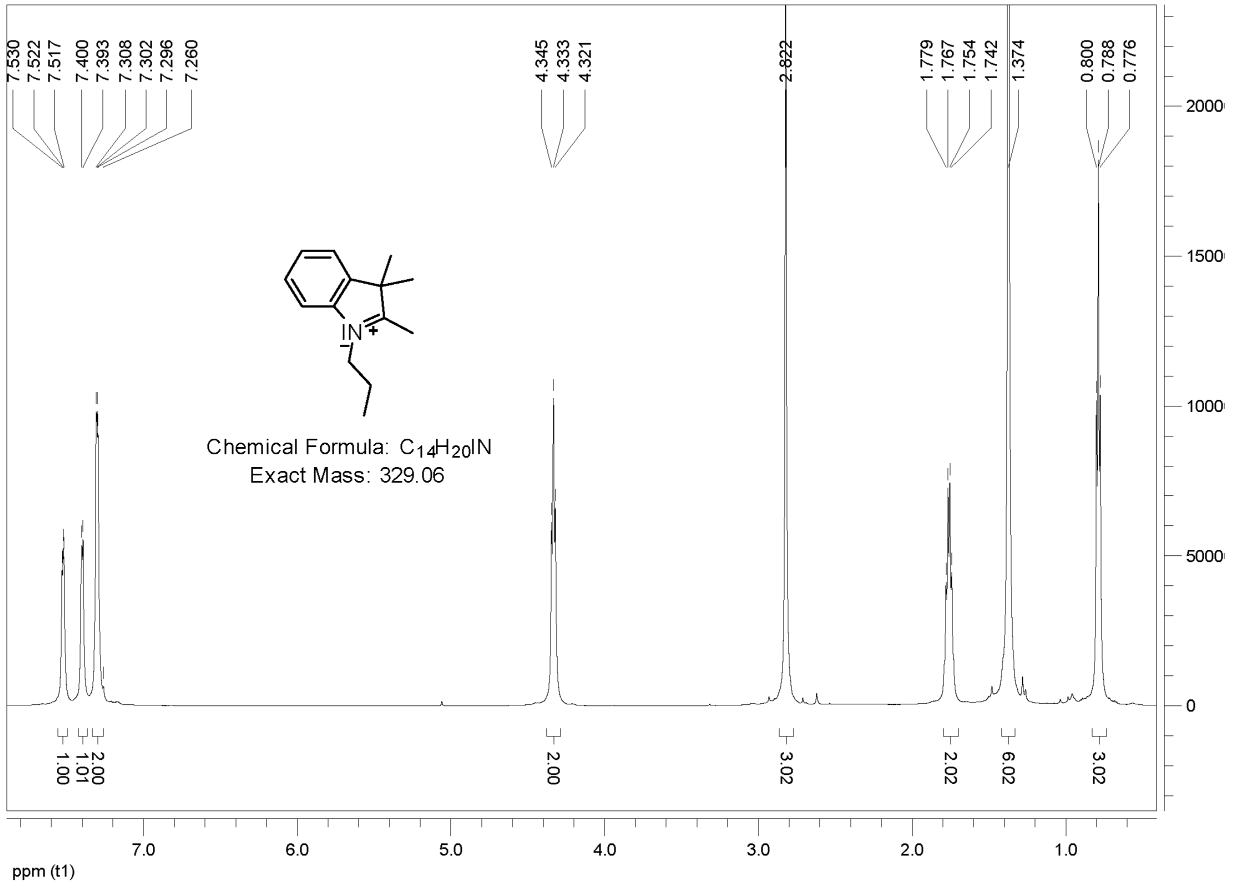

Figure S1. ^{1}H NMR spectrum of compound **1b** (600 MHz, $CDCl_3$).

Scheme 3. Synthesis of **1**

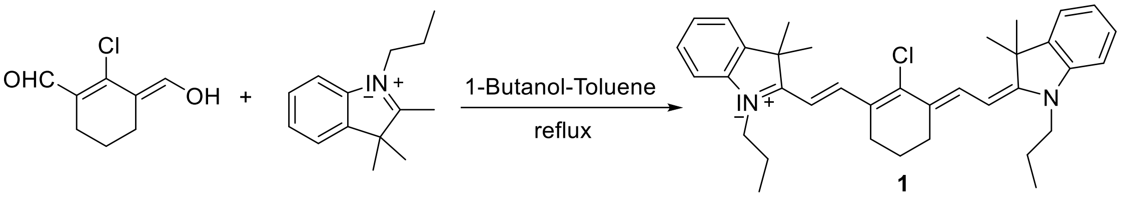

1a (500 mg, 2.9 mmol, 1 eq.) and **1b** (1.91 g, 5.81 mmol, 2 eq.) were dissolved in 50 mL solution of BuOH-Toluene (7: 3) under N_2 atmosphere, and the mixture refluxed at 160 °C for 18 h with a Dean-Stark condenser. Afterwards, the solvent was evaporated, and the resulting green solid mixture was washed with Et_2O and purified by silica gel flash chromatography (eluent: DCM-MeOH, 50: 1) to obtain **1** as a green solid (1.8 g, 96%). ^1H NMR (600 MHz, $CDCl_3$) δ = 8.34 (d, J = 14.4 Hz, 2H), 7.40-7.36 (m, 4H), 7.26-7.20 (m, 2H), 7.17 (dd, J = 8.4, 3.6Hz, 2H), 6.26 (d, J = 14.4 Hz, 2H), 4.19 (dd, J = 9.6, 4.8 Hz, 4H), 2.76 (dd, J = 16.2, 10.2 Hz, 4H), 1.78 (s, 2H), 1.72 (s, 12H), 1.60 (s, 4H), 1.07 (t, J = 7.2 Hz, 6H).

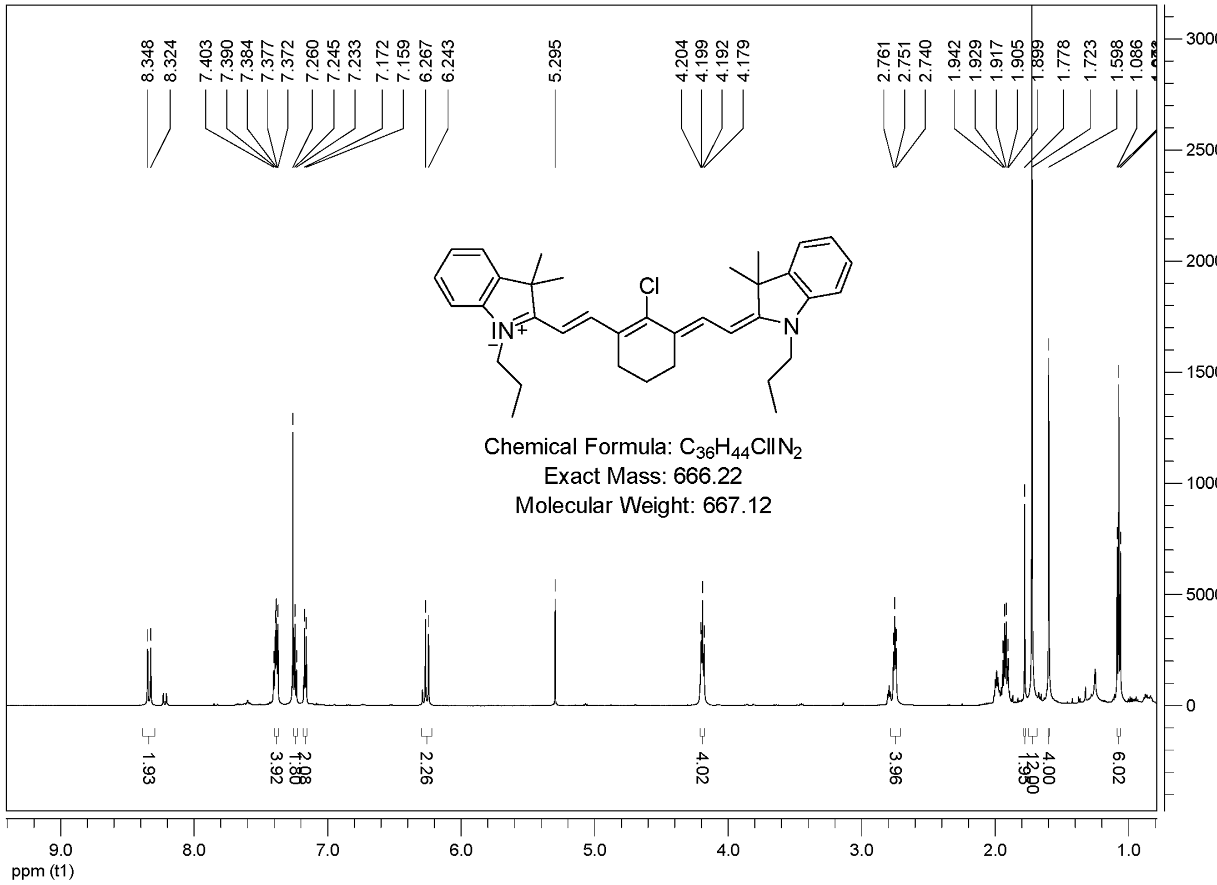

Figure S2. ^{1}H NMR spectrum of compound **1** (600 MHz, $CDCl_3$).

Scheme 4. Synthesis of **2**

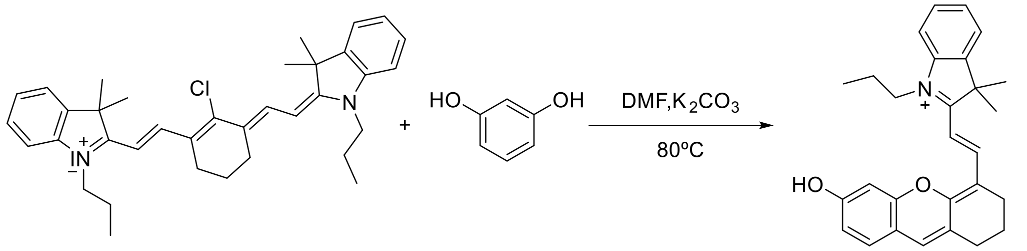

Under N_2, resorcinol (12 mg, 0.11 mmol, 1 eq.) and K_2CO_3 (15 mg, 0.11 mmol, 1 eq.) were dissolved in 10 mL of dimethylformamide, and the mixture was stirred at 80 °C until the solution turned brown. Then, a mixture of **1** (73.4 mg, 0.11 mmol, 1 eq.) was added to the reaction system. The reaction was stirred at 80 °C for 4 h, then allowed to cool to room temperature, and then condensed under reduced pressure, and the residue was purified by silica gel chromatography with CH_2Cl_2/MeOH (100: 1) as eluent to yield **2** (44 mg, 74%). ^{1}H NMR (600 MHz, $CDCl_3$) δ = 8.07 (d, J = 13.2 Hz, 1H), 7.32 (s, 1H), 7.30-7.25 (m, 2H). 7.20 (t, J = 9.6 Hz, 1H), 7.05 (t, J = 7.2 Hz, 1H), 6.85 (d, J = 7.8 Hz, 1H), 6.76 (d, J = 8.4 Hz, 1H), 6.55 (s, 1H), 5.65 (d, J = 13.2 Hz, 1H), 3.78 (t, J = 7.2Hz, 2H), 2.64 (td, J =

34.2, 5.4 Hz, 4H), 1.93-1.87 (m, 2H), 1.82 (dd, J = 14.4, 7.2 Hz, 2H), 1.67 (s, 6H), 1.04 (t, J = 7.2 Hz, 3H).

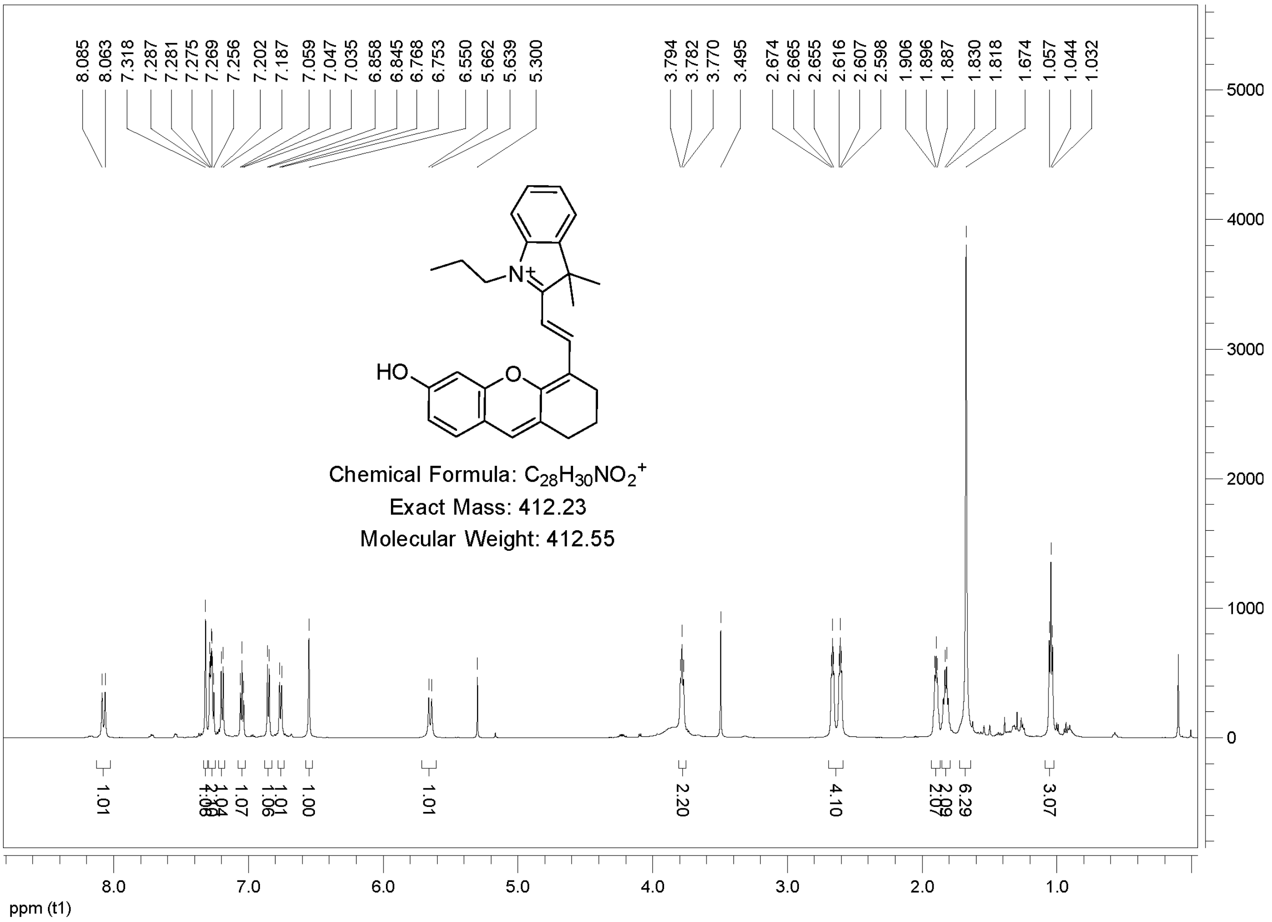

Figure S3. ^{1}H NMR spectrum of compound **2** (600 MHz, $CDCl_3$).

Scheme 5. Synthesis of **CDIA**

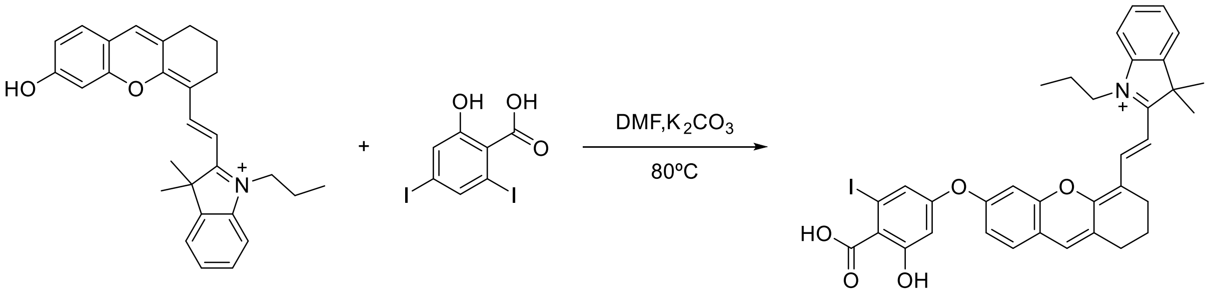

Compound **2** (140 mg, 0.26 mmol, 1 eq.) and K_2CO_3 (196 mg, 1.4 mmol, 5.4 eq.) were mixed and stirred in anhydrous 20 mL dimethylformamide under N_2 atmosphere for 30 min before 3,5-diiodosalicyclic acid (546 mg, 1.4 mmol, 5.4 eq.) dissolved in 5 mL anhydrous dimethylformamide

was added *via* a syringe. The mixture was stirred for 3 h under 80 °C and concentrated under reduced pressure. and the residue was purified by silica gel chromatography with CH_2Cl_2/ MeOH as eluent (100: 1, then 10: 1) to yield **CDIA** (25 mg, 12 %). ^{1}H NMR (600 MHz, $CDCl_3$): δ = 8.79-8.61 (m, 1H), 8.33 (s, 1H), 8.00 (s, 1H), 7.83 (s, 1H), 7.76-7.75 (m, 1H), 7.43-7.28 (m, 4H), 7.10 (d, *J* = 7.8 Hz, 1H), 7.09-7.04 (m, 1H), 5.98 (d, *J* = 13.8 Hz, 1H), 4.00 (t, *J* = 7.2 Hz, 2H), 2.77-2.75 (m, 2H), 2.62-2.60 (m, 2H), 1.95-1.90 (m, 4H), 1.77 (s, 6H), 1.08 (t, *J* = 7.2 Hz, 3H). ^{13}C NMR (400 MHz, $CDCl_3$): δ =175.38, 174.04, 171.92, 171.53, 166.03, 164.01, 161.50, 155.51, 148.87, 144.37, 141.42, 139.54, 138.54, 128.77, 128.65, 126.23, 124.09, 123.43, 120.29, 118.08, 114.44, 114.02, 110.64, 103.32, 99.52, 86.32, 50.16, 31.61, 29.73, 28.57, 24.02, 22.68, 20.85, 14.16, 11.70. ESI-MS: $C_{35}H_{33}INO_5$ calculated for: 674.1[M – I$^-$]; found: 674.3 [M-I$^-$]$^+$.

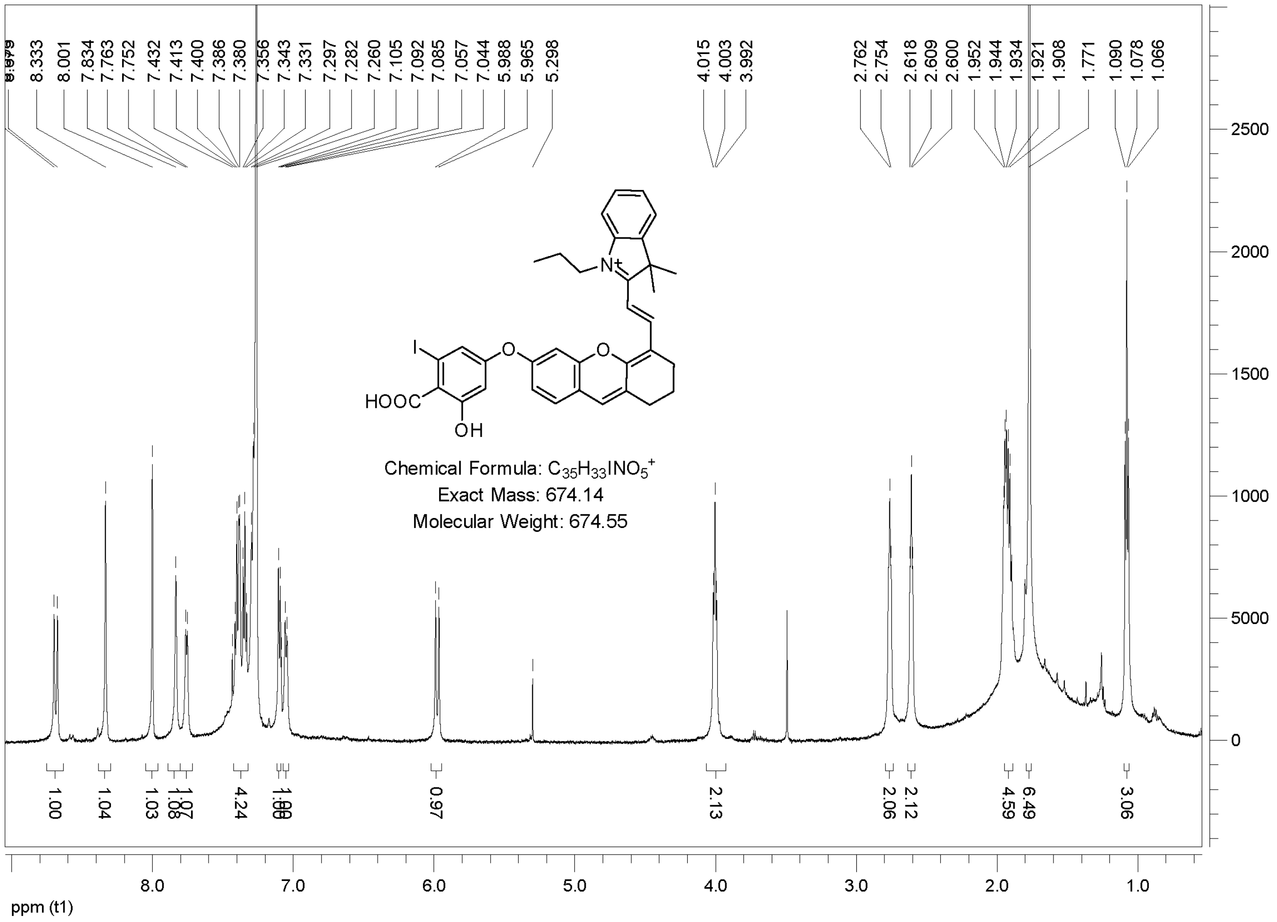

Figure S4. ^{1}H NMR spectrum of compound **CDIA** (600 MHz, $CDCl_3$).

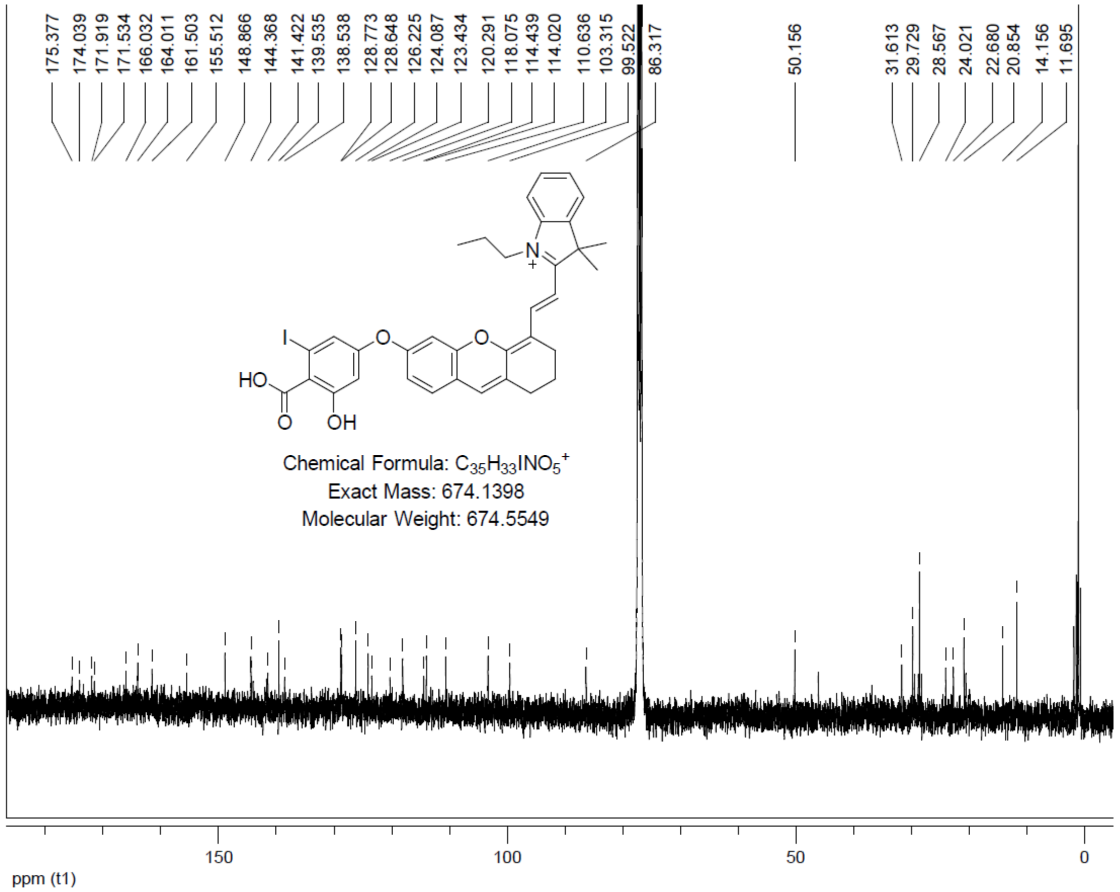

Figure S5. ^{13}C NMR spectrum of compound **CDIA** (400 MHz, $CDCl_3$).

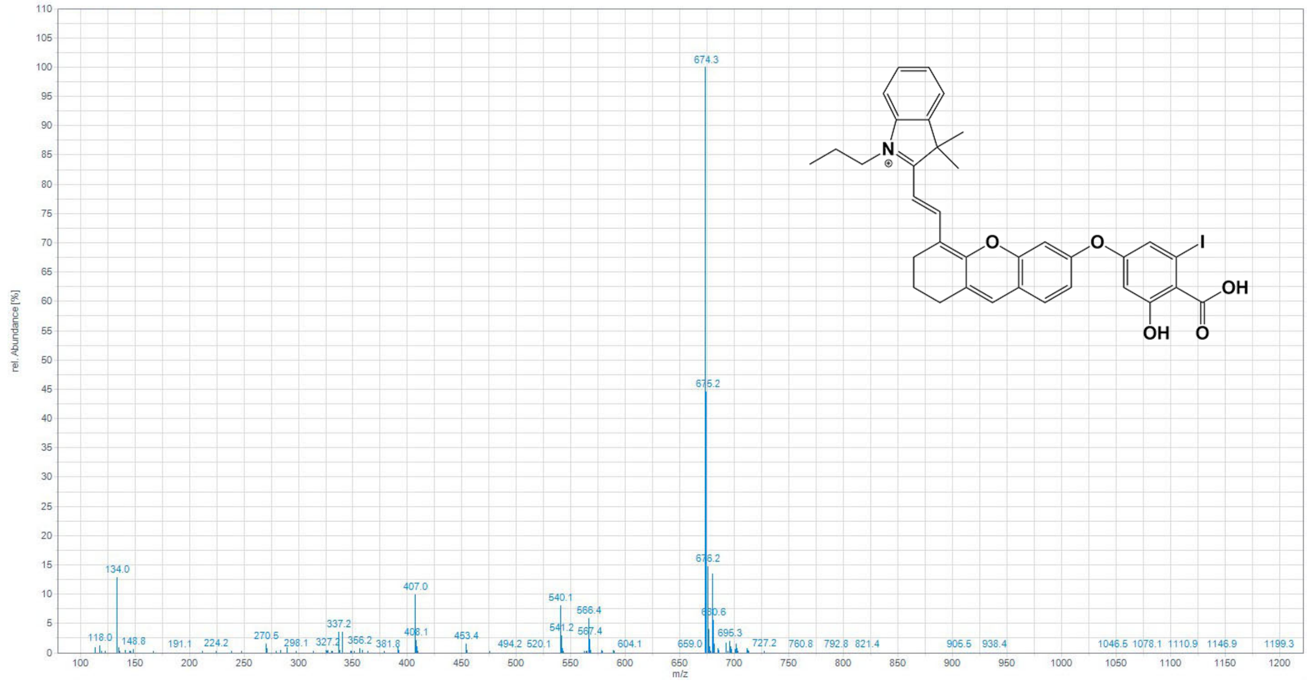

Figure S6. Mass spectrum of CDIA.

Supporting Figures and Tables

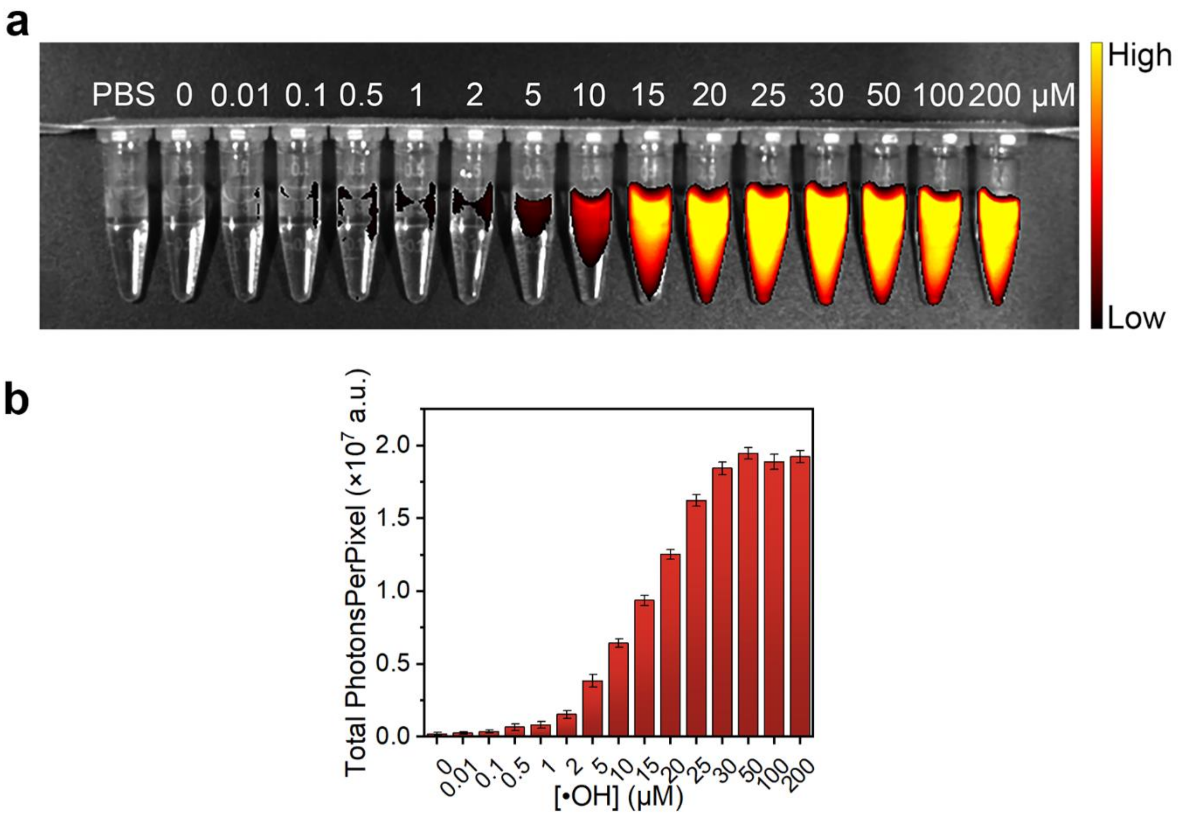

Figure S7. (a) NIR fluorescence images of CDIA (10 μM) incubated with different concentration of •OH in PBS buffer (pH = 7.4) at 37 ºC for 30 min. The fluorescence images were acquired at 720 nm upon excitation at 650 nm. (b) Quantification of NIR fluorescence intensities of CDIA (10 μM) after treatment with various conditions in (a). Data represent mean ± SD (n = 5).

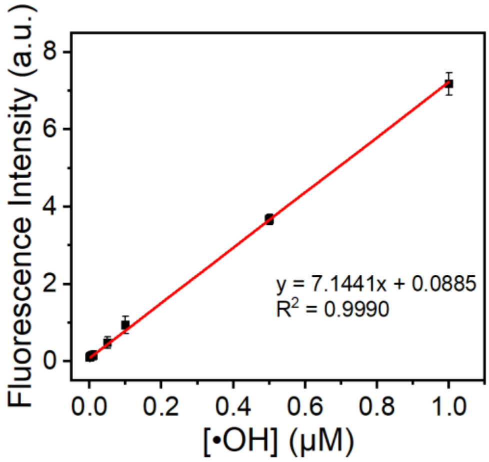

Figure S8. Linear correlation between the fluorescence intensity and •OH concentration. All

measurements were acquired in PBS (10 mM, pH 7.4) at 37 °C, $\lambda_{ex/em}$ = 650/720 nm. Data represent mean ± SD (n = 5).

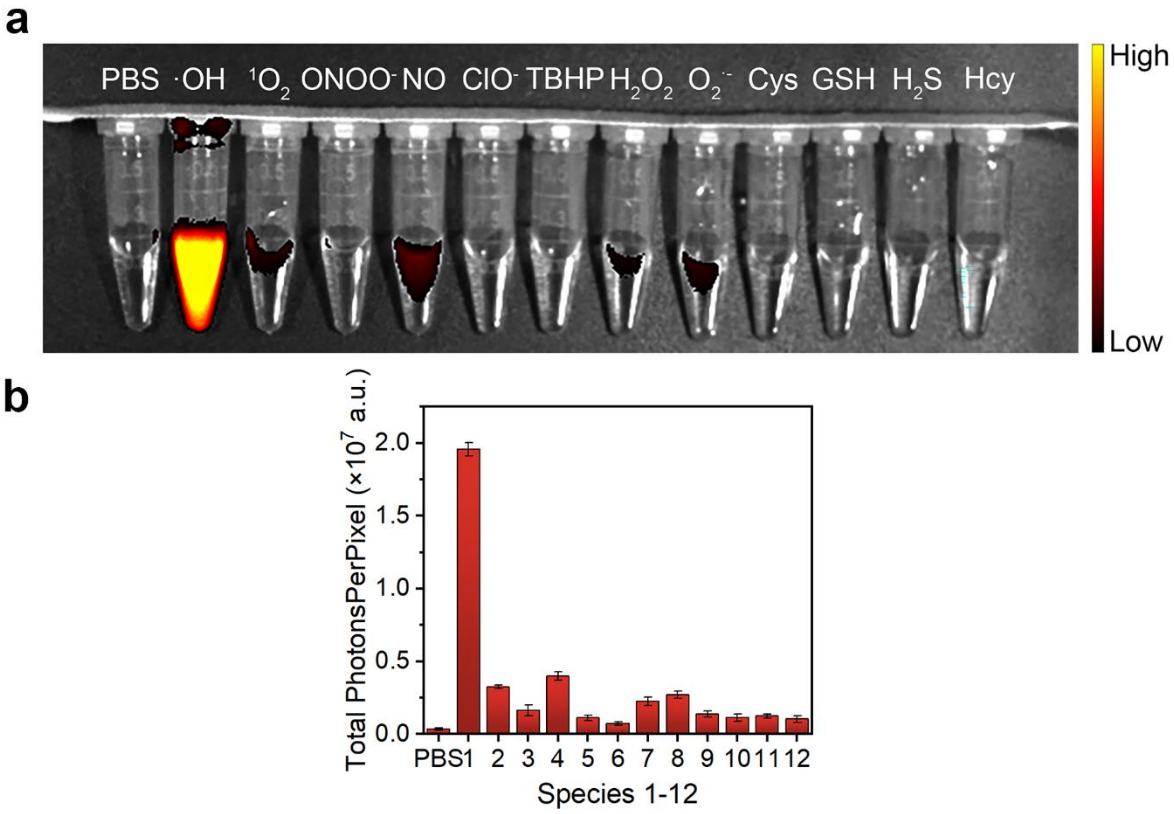

Figure S9. (a) NIR fluorescence images of CDIA (10 µM) incubated with different analytes in PBS buffer (pH = 7.4) at 37 ºC for 30 min. The fluorescence images were acquired at 720 nm upon excitation at 650 nm. (b) Quantification of NIR fluorescence intensities of CDIA (10 µM) after treatment with various conditions in (a). Data represent mean ± SD (n = 5).

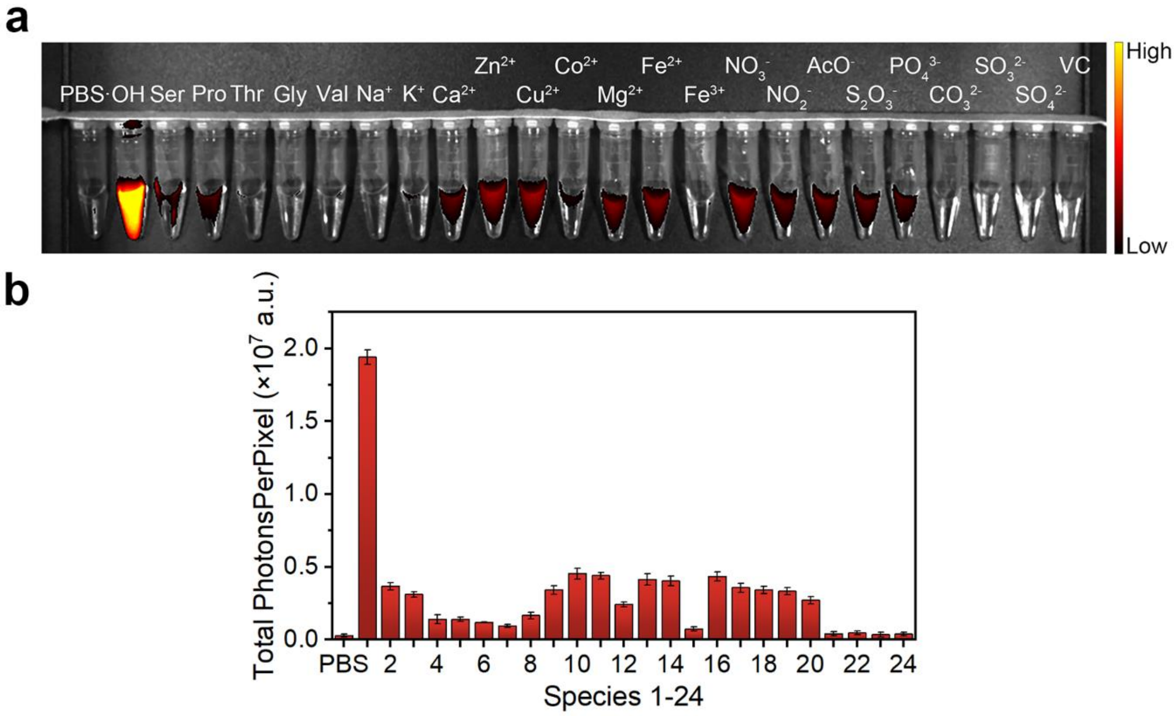

Figure S10. (a) NIR fluorescence images of CDIA (10 μM) incubated with different analytes in PBS buffer (pH = 7.4) at 37 ºC for 30 min. The fluorescence images were acquired at 720 nm upon excitation at 650 nm. (b) Quantification of NIR fluorescence intensities of CDIA (10 μM) after treatment with various conditions in (a). Data represent mean ± SD (n = 5).

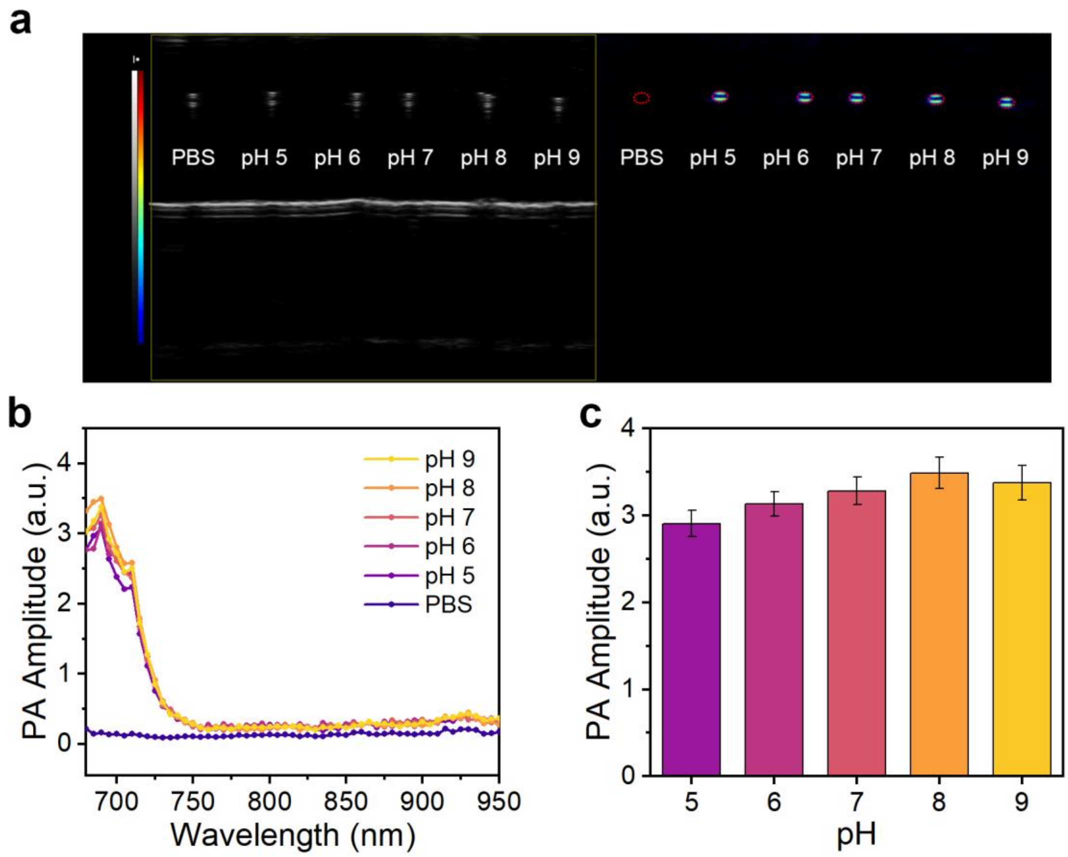

Figure S11. (a) PA imaging of CDIA (10 μM) with •OH (50 μM) at various pH values at 37 °C for 30 min. (b) The PA spectra of CDIA (10 μM) with •OH (50 μM) at various pH values at 37 °C for 30 min. (c) The PA amplitude plots of CDIA (10 μM) with •OH (50 μM) at various pH values at 37 °C for 30 min. Data represent mean ± SD (n = 5).

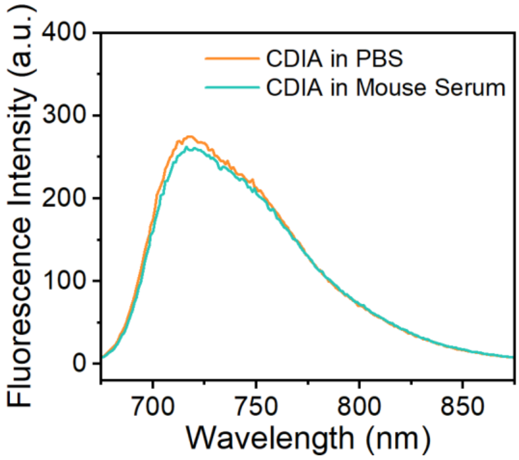

Figure S12. Fluorescence spectra of CDIA (10 μM) in the absence or presence of 50 μM •OH in PBS or DMEM with 10% FBS at 37 °C, λ_{ex} = 650 nm.

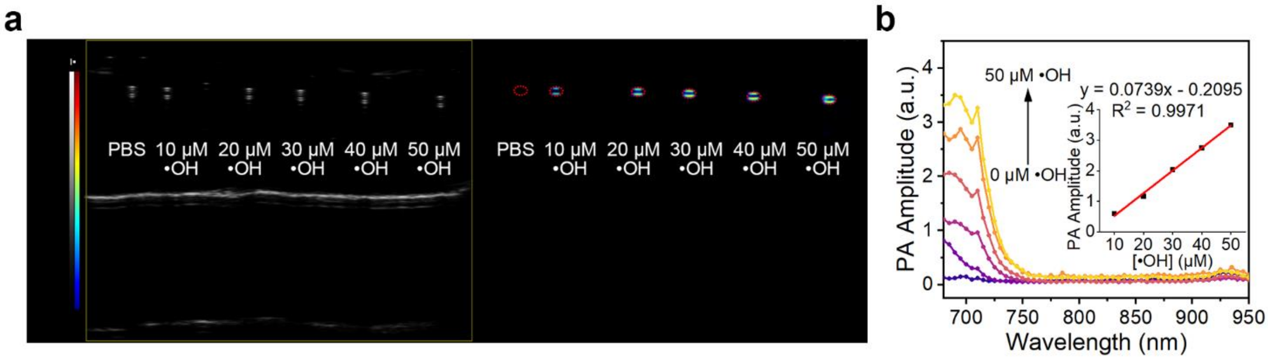

Figure S13. (a) PA imaging of CDIA (10 μM) after incubation with varied concentrations of •OH (0−50 μM) in PBS buffer (10 mM, pH 7.4) at 37 °C for 30 min. (b) PA spectra of CDIA (10 μM) after incubation with varied concentrations of •OH (0−50 μM) in PBS buffer (10 mM, pH 7.4) at 37 °C for 30 min. Inset: The relationship between the concentration of •OH and the PA amplitude of CDIA.

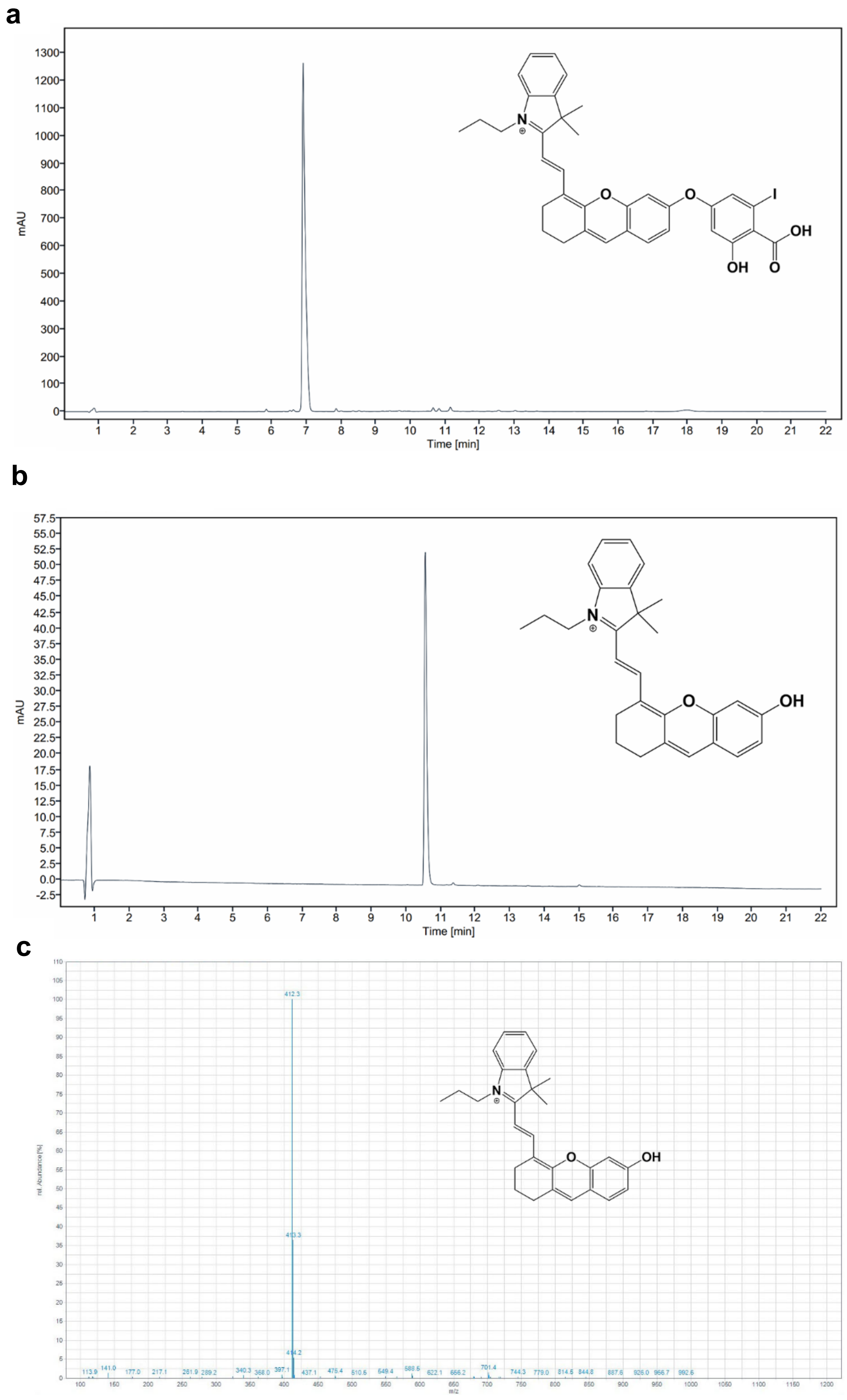

Figure S14. (a) HPLC chromatogram of CDIA. (b) HPLC chromatogram of reaction solution of CDIA with •OH. (c) Mass spectrum of reaction solution of CDIA with •OH. Detection of products obtained after reaction of CDIA with •OH.

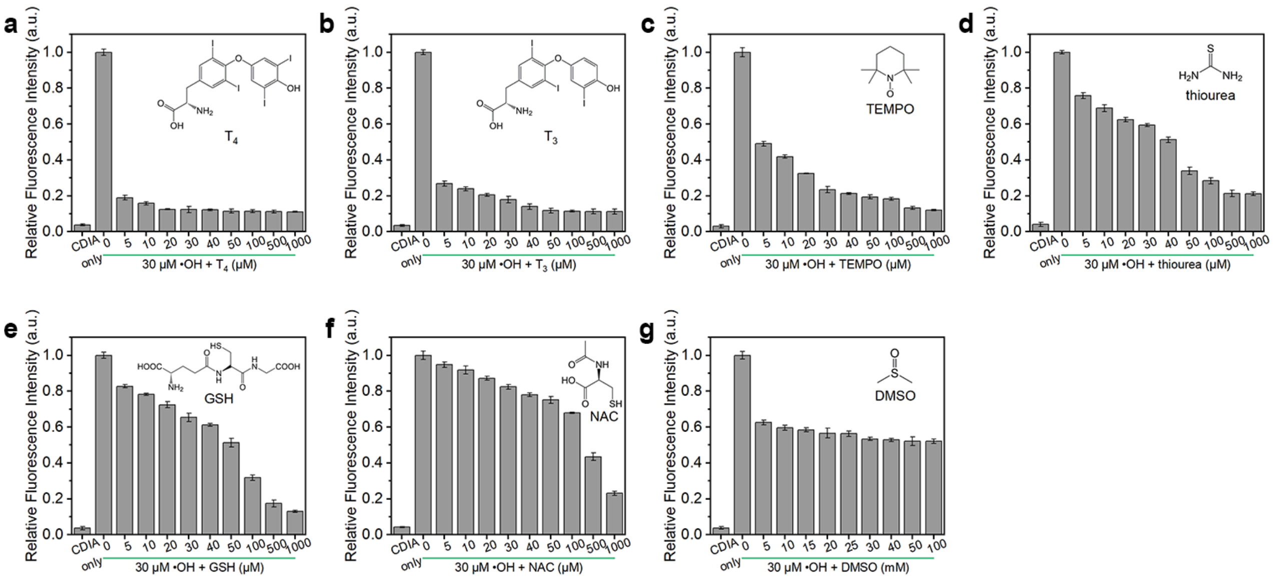

Figure S15. Investigating the •OH scavenging ability of (a) T_4; (b) T_3; (c) TEMPO; (d) thiourea; (e) GSH; (f) NAC and (g) DMSO with CDIA (10 μM) in PBS buffer (10 mM, pH 7.4) at 37 °C for 30 min, $\lambda_{ex}/_{em}$ = 650/720 nm. Data represent mean ± SD (n = 5).

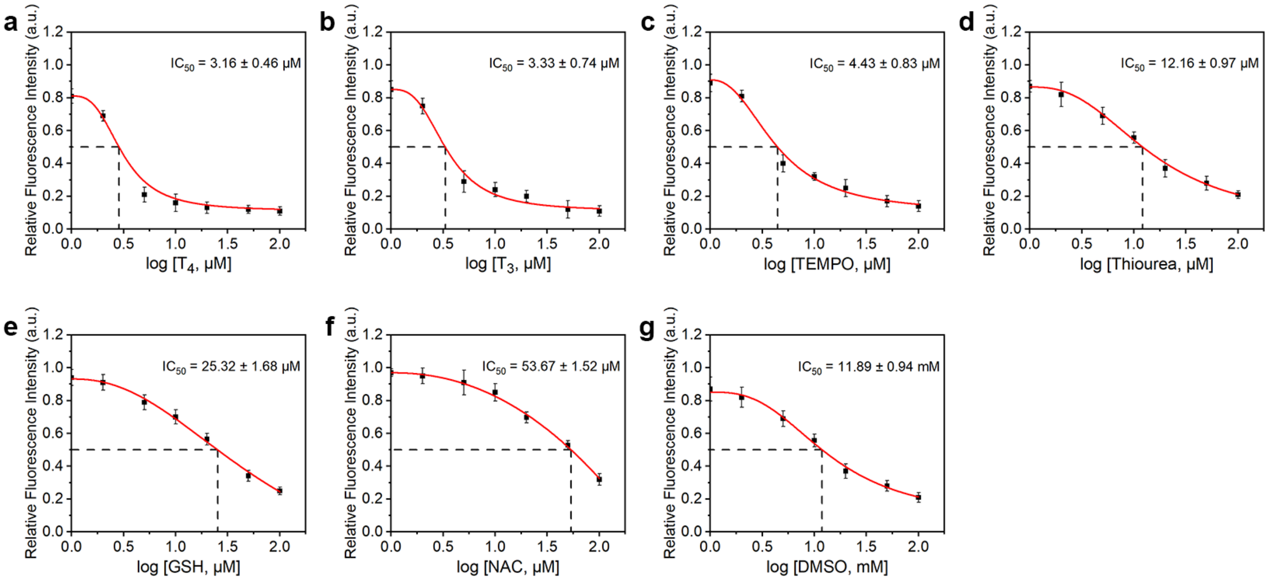

Figure S16. Chemical •OH scavenging by (a) T_4; (b) T_3; (c) TEMPO; (d) thiourea; (e) GSH; (f) NAC and (g) DMSO with CDIA (10 μM) in PBS buffer at 37 °C, $\lambda_{ex/em}$ = 650/720 nm. Data represent mean ± SD (n = 5).

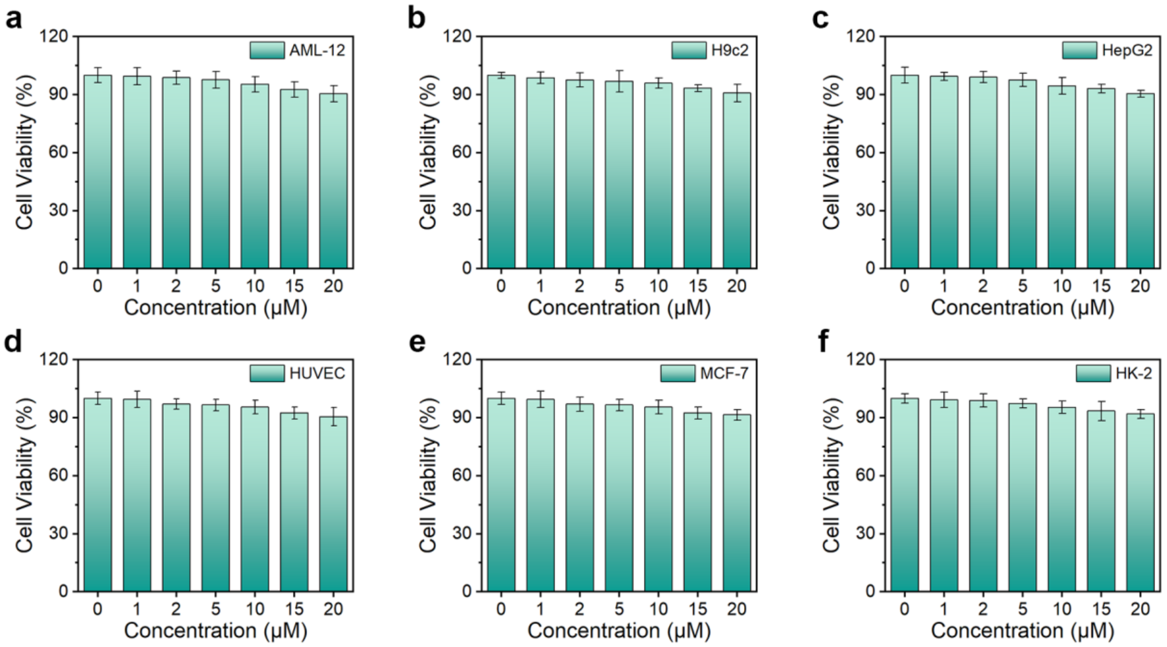

Figure S17. Cell viability assay of AML-12, H9c2, HepG2, HUVEC, MCF-7 and HK-2 cells after being incubated with different concentrations of CDIA at 37 °C for 24 h. Data represent mean ± SD (n = 5).

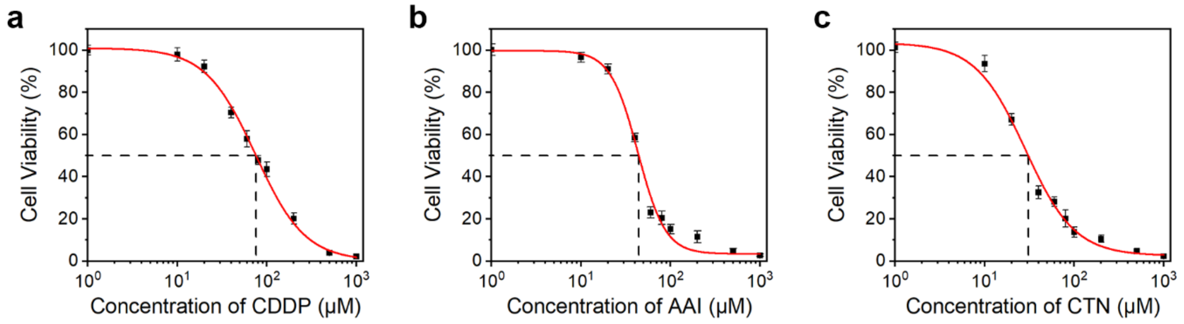

Figure S18. MTT assay for the cell viability of HK-2 cells incubated with drugs at different concentrations. Dose-response curves of HK-2 cells after 24 h treatment with 1 ~ 1000 μM (a) CDDP, (b) AAI, (c) CTN, respectively. Data represent means ± SD (n = 5).

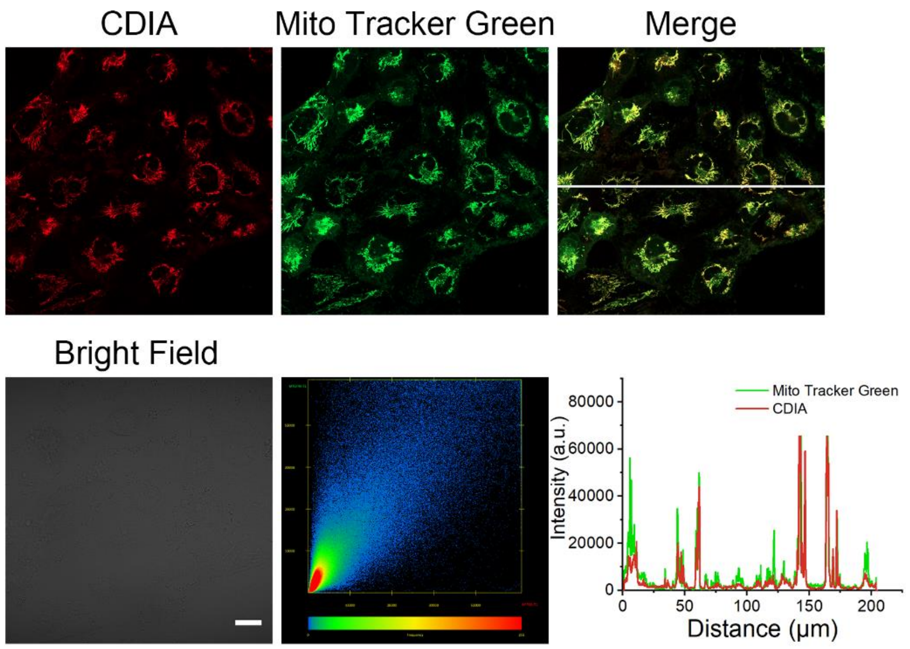

Figure S19. Confocal fluorescence images of 75 μM CDDP-treated HK-2 cells co-stained with CDIA and Mito Tracker Green (Pearson's correlation R_r = 0.8652). Scale bar: 20 μm.

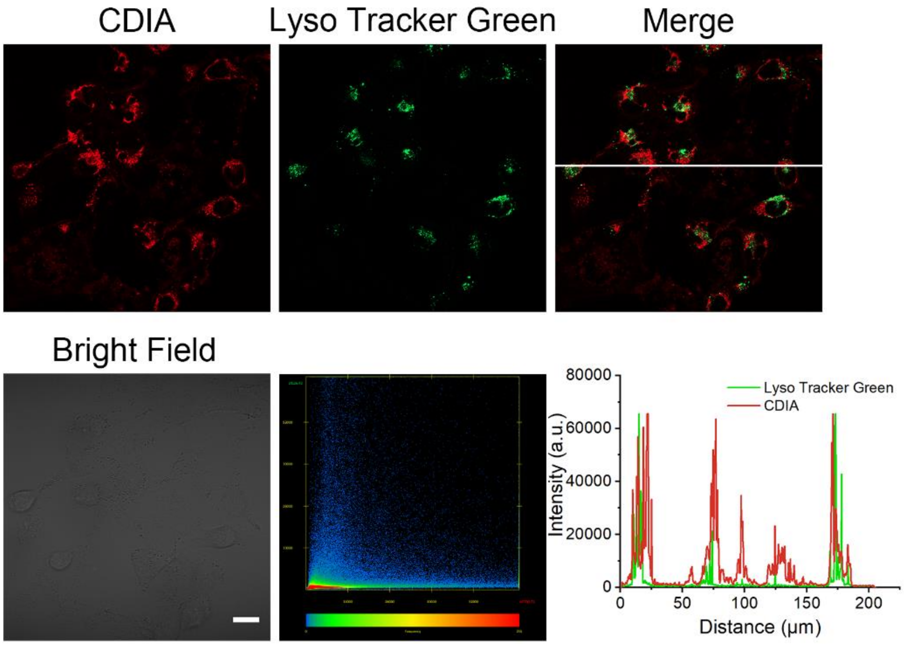

Figure S20. Confocal fluorescence images of 150 μM CDDP-treated HK-2 cells co-stained with CDIA and Lyso Tracker Green (Pearson's correlation R_r = 0.2532). Scale bar: 20 μm.

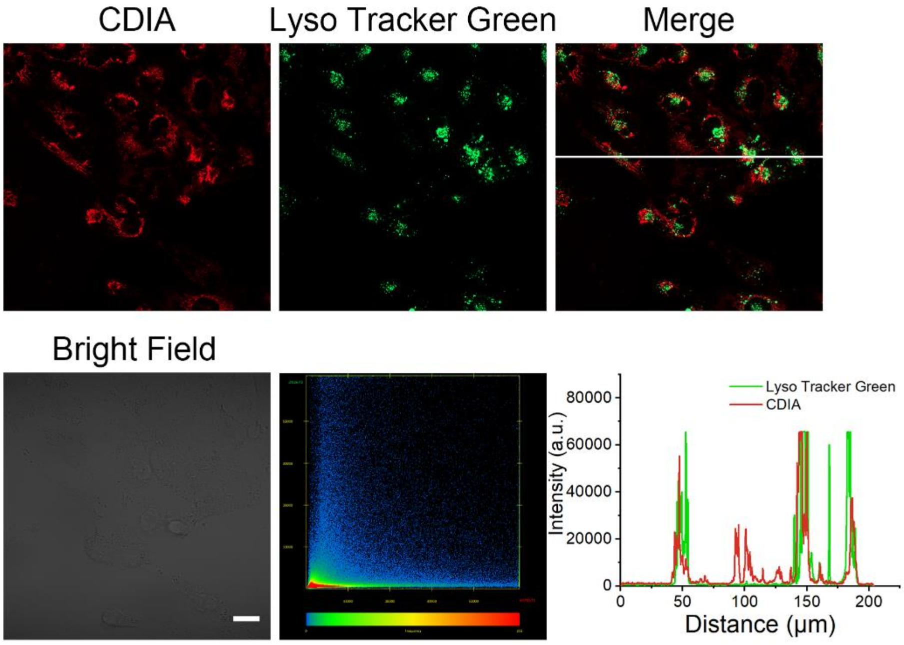

Figure S21. Confocal fluorescence images of 75 μM CDDP-treated HK-2 cells co-stained with CDIA and Lyso Tracker Green (Pearson's correlation R_r = 0.2469). Scale bar: 20 μm.

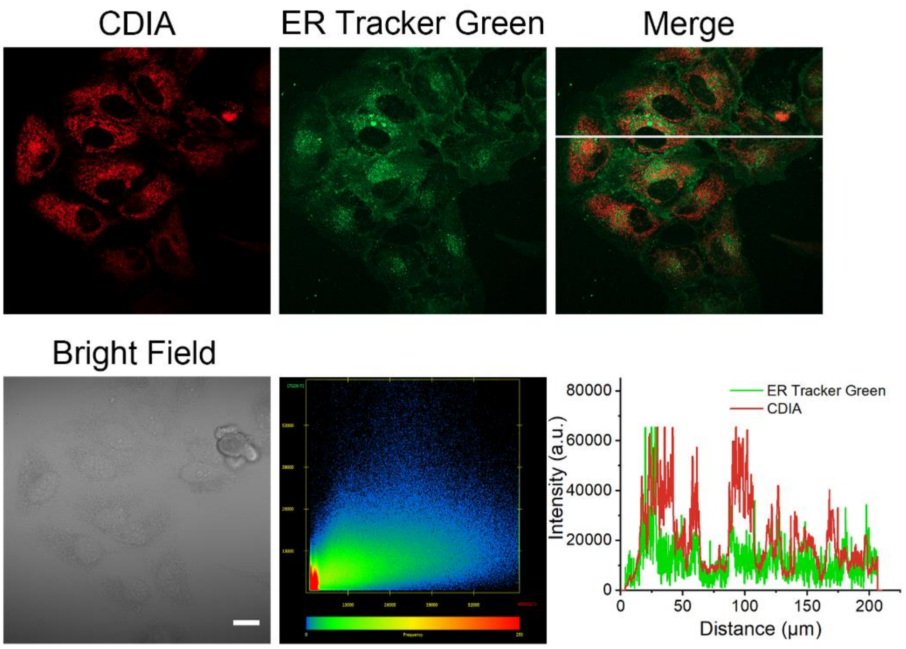

Figure S22. Confocal fluorescence images of 150 μM CDDP-treated HK-2 cells co-stained with CDIA and ER Tracker Green (Pearson's correlation R_r = 0.3064). Scale bar: 20 μm.

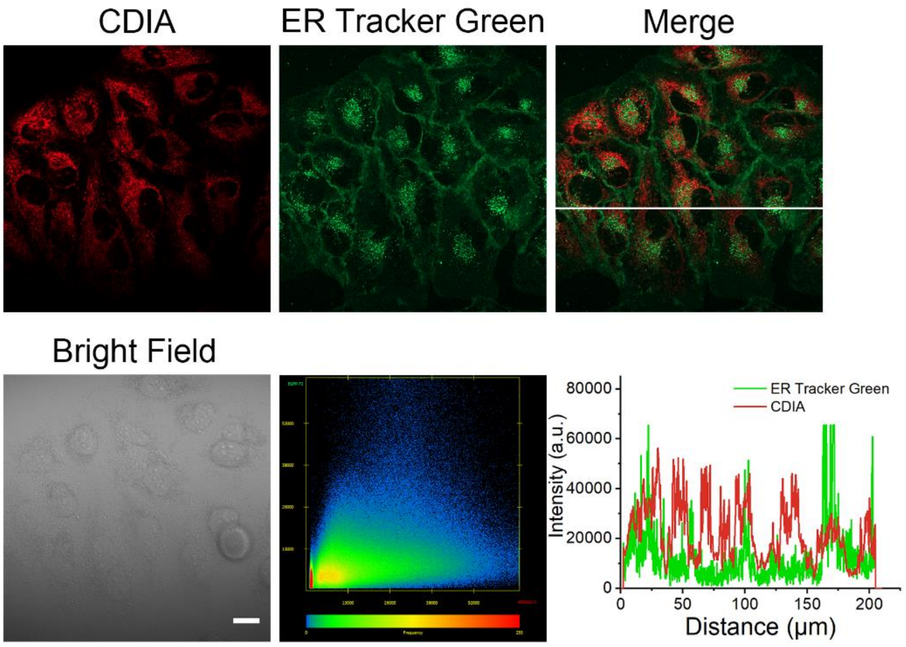

Figure S23. Confocal fluorescence images of 75 μM CDDP-treated HK-2 cells co-stained with CDIA and ER Tracker Green (Pearson's correlation R_r = 0.2749). Scale bar: 20 μm.

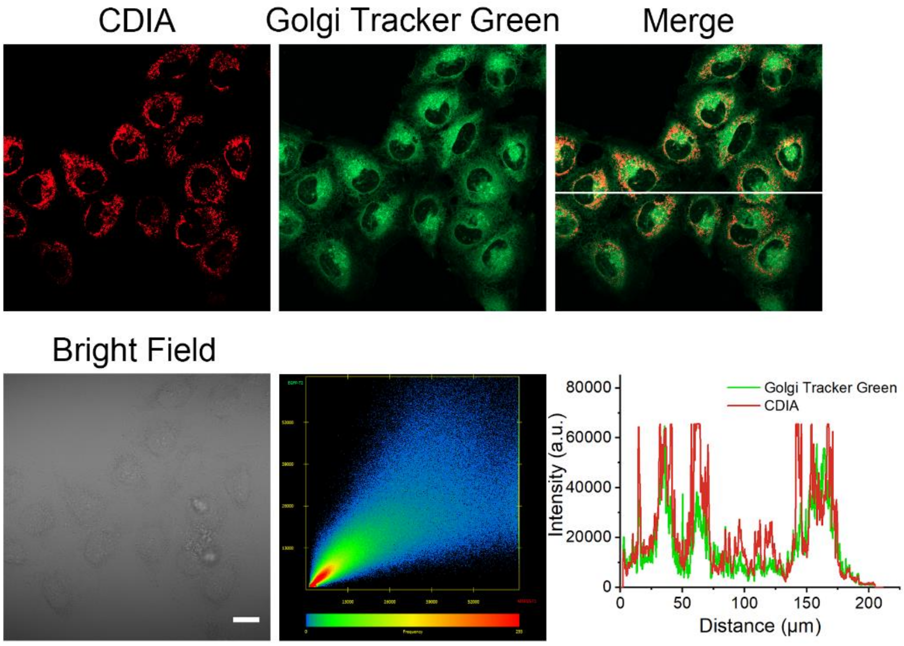

Figure S24. Confocal fluorescence images of 150 μM CDDP-treated HK-2 cells co-stained with CDIA and Golgi Tracker Green (Pearson's correlation R_r = 0.4683). Scale bar: 20 μm.

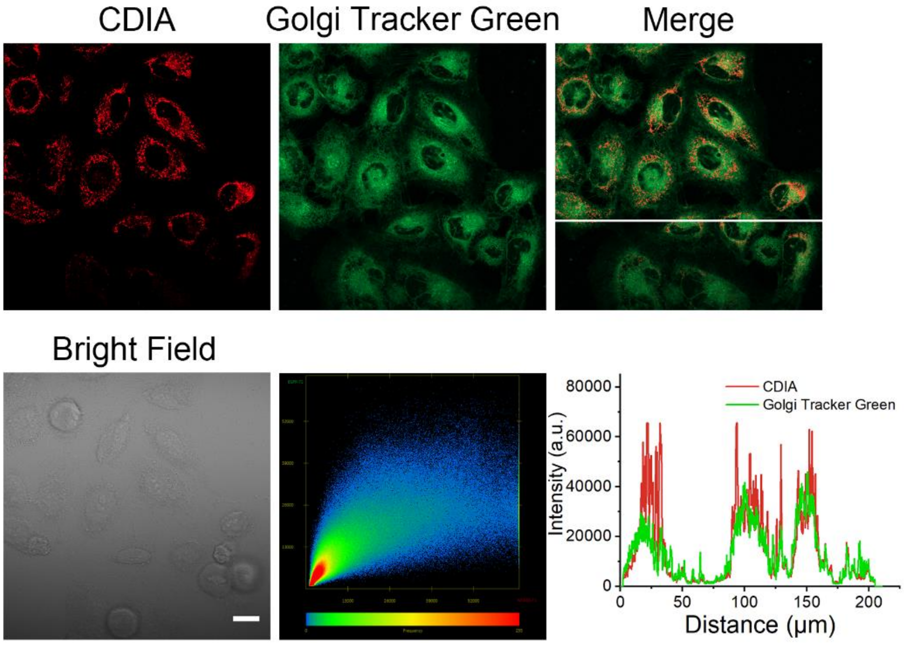

Figure S25. Confocal fluorescence images of 75 μM CDDP-treated HK-2 cells co-stained with CDIA and Golgi Tracker Green (Pearson's correlation R_r = 0.3771). Scale bar: 20 μm.

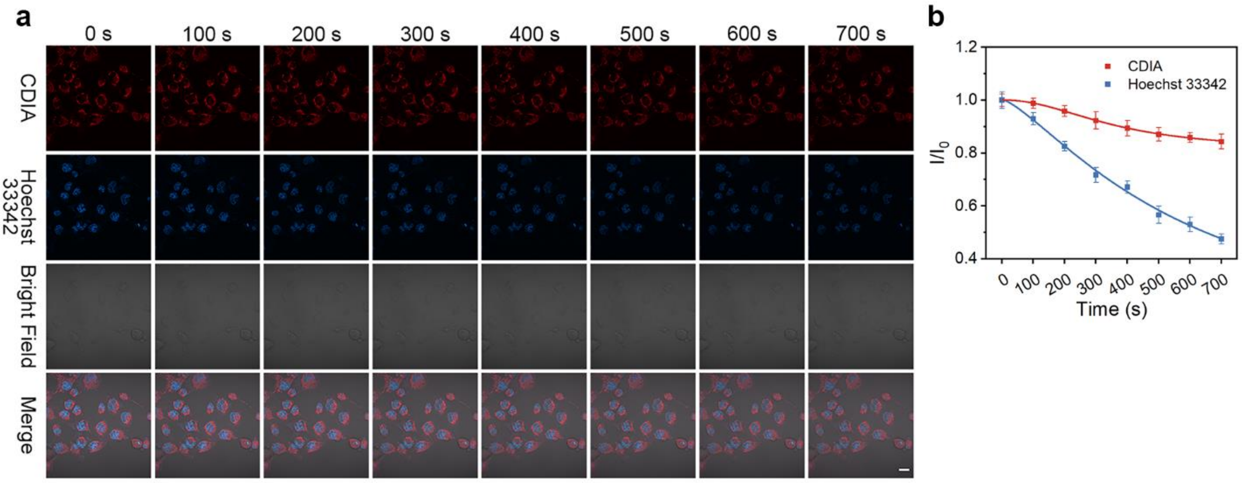

Figure S26. (a) Confocal fluorescence imaging of HK-2 cells incubated with 10 μM CDIA and 10 μg/mL Hoechst 33342, respectively. After the incubated cells were mounted on the microscope stage, eight images were taken consecutively at 100 s intervals. Scale bar: 20 μm. (b) Fluorescence intensity plots of HK-2 cells incubated with 10 μM CDIA (red) and 10 μg/mL Hoechst 33342 (blue), respectively. Data represent mean ± SD (n = 5).

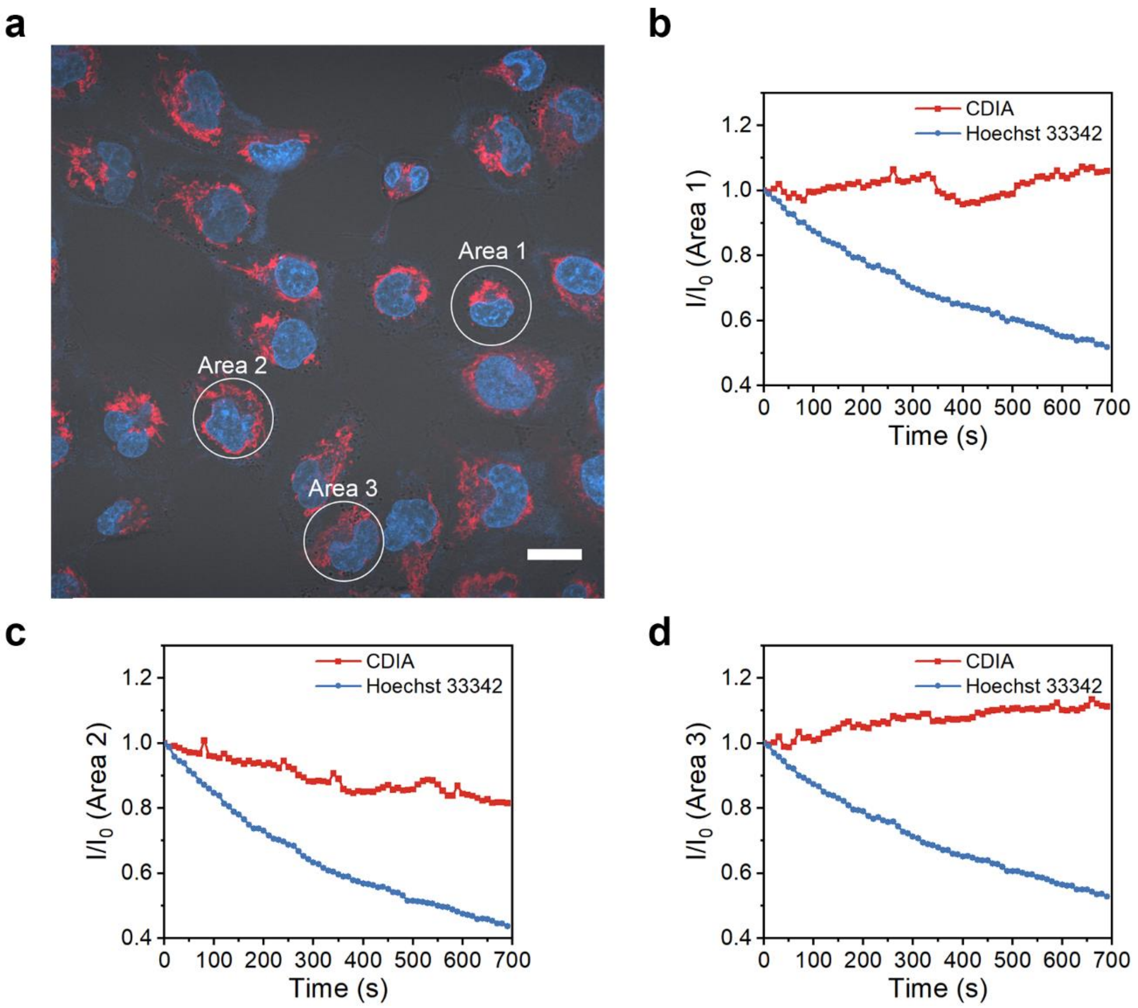

Figure S27. (a) Merged fluorescence and bright field image during laser irradiation. Scale bar: 20 mm. (b–d) Time course of fluorescence intensity collected at circles 1–3 shown in (a), corresponding to the areas (1–3).

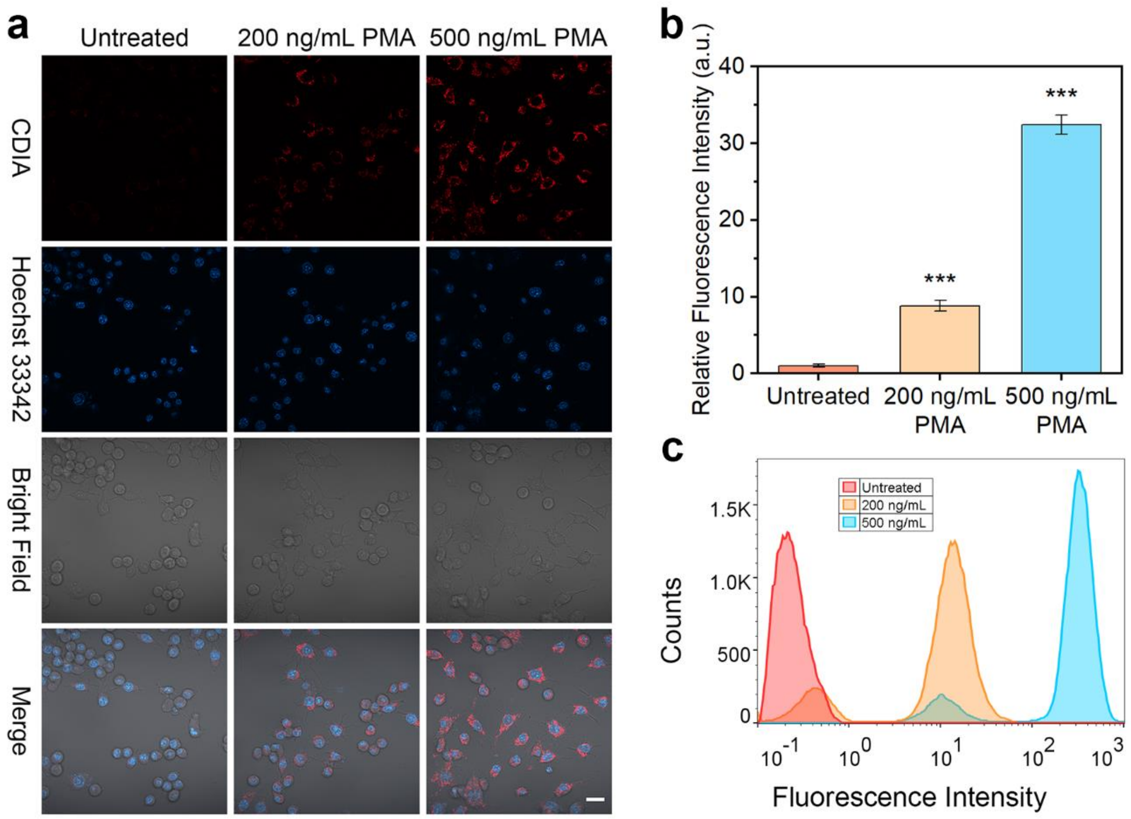

Figure S28. (a) Confocal fluorescence imaging of RAW264.7 cells with untreated, 200 ng/mL PMA or 500 ng/mL PMA for 4 h, then incubated with 10 µM CDIA at 37 °C for 30 min. Scale bar: 20 µm. (b) Fluorescence intensity plots of RAW264.7 cells for different groups. (c) Flow cytometric assays of RAW264.7 cells for different groups. Data represent mean ± SD (n = 5), **P*<0.05, ***P*<0.01, ****P*<0.001 for treatment compared to the control group using one-way ANOVA with multiple comparisons test.

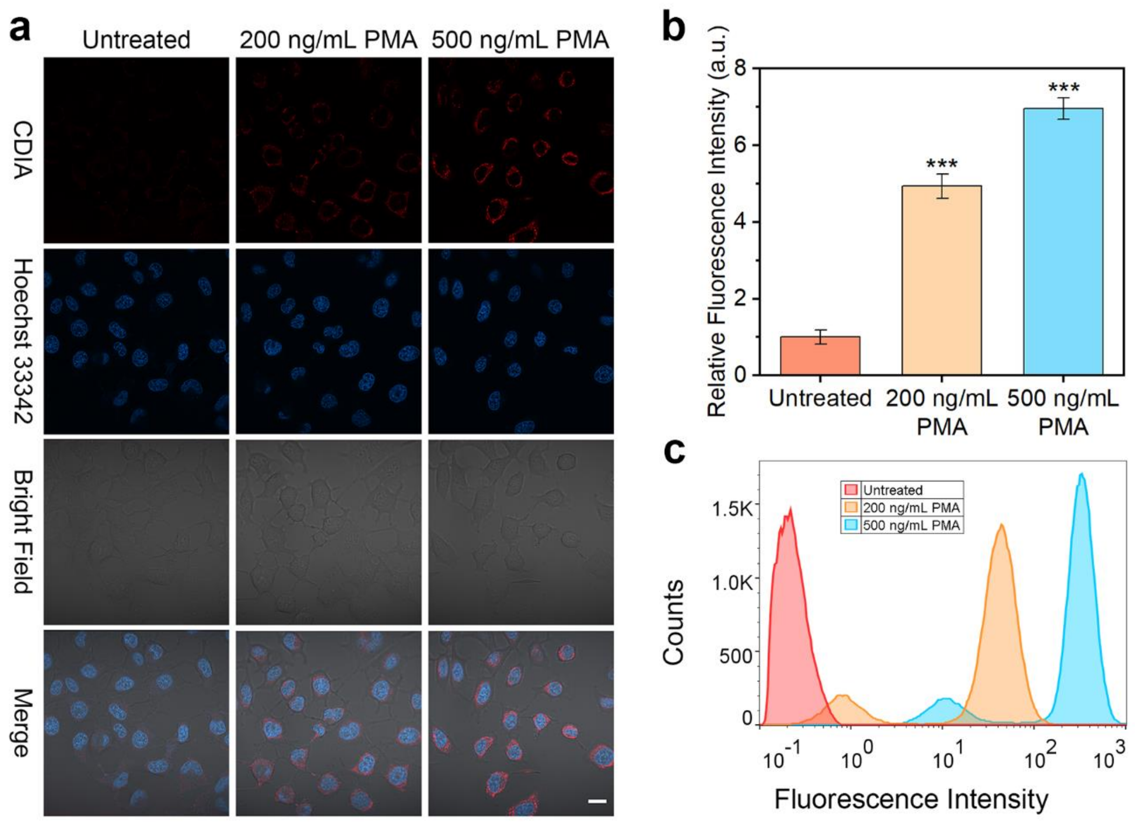

Figure S29. (a) Confocal fluorescence imaging of HeLa cells with untreated, 200 ng/mL PMA or 500 ng/mL PMA for 4 h, then incubated with 10 μM CDIA at 37 °C for 30 min. Scale bar: 20 μm. (b) Fluorescence intensity plots of HeLa cells for different groups. (c) Flow cytometric assays of HeLa cells for different groups. Data represent mean ± SD (n = 5), **P*<0.05, ***P*<0.01, ****P*<0.001 for treatment compared to the control group using one-way ANOVA with multiple comparisons test.

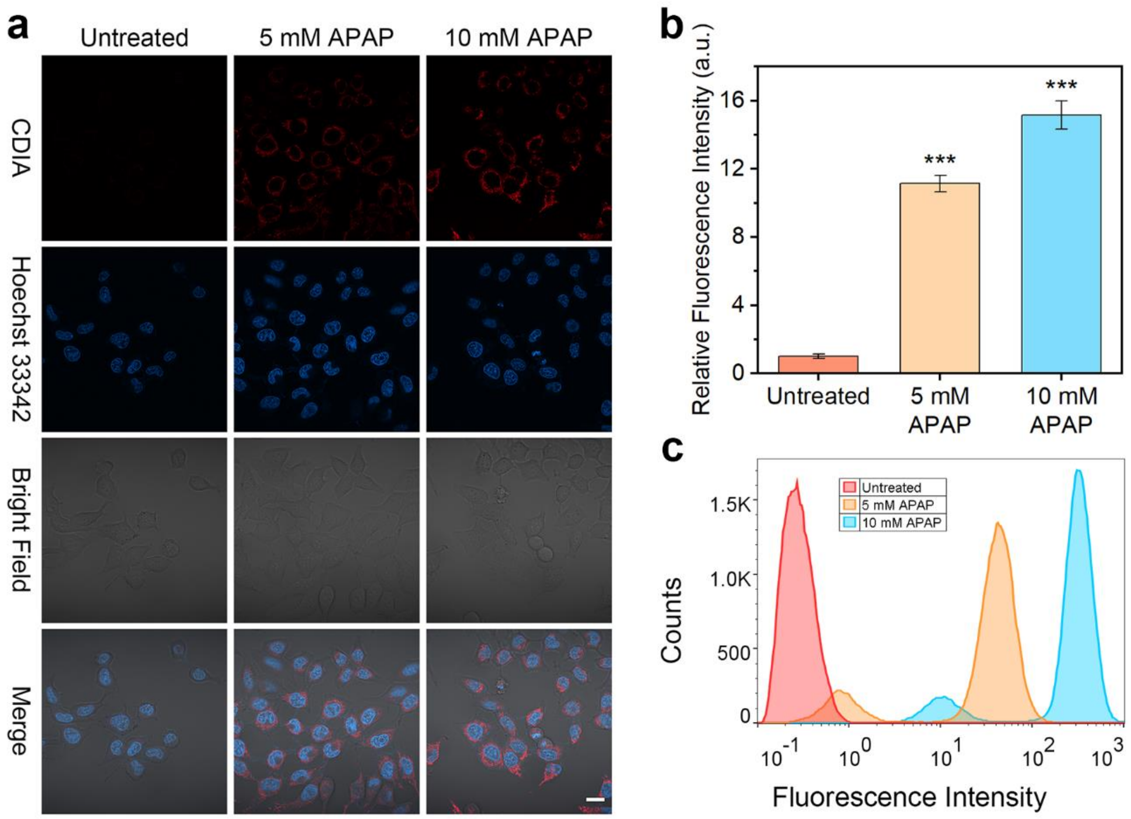

Figure S30. (a) Confocal fluorescence imaging of HepG2 cells with untreated, 5 mM APAP or 10 mM APAP for 24 h, then incubated with 10 μM CDIA at 37 °C for 30 min. Scale bar: 20 μm. (b) Fluorescence intensity plots of HepG2 cells for different groups. (c) Flow cytometric assays of HepG2 cells for different groups. Data represent mean ± SD (n = 5), **P*<0.05, ***P*<0.01, ****P*<0.001 for treatment compared to the control group using one-way ANOVA with multiple comparisons test.

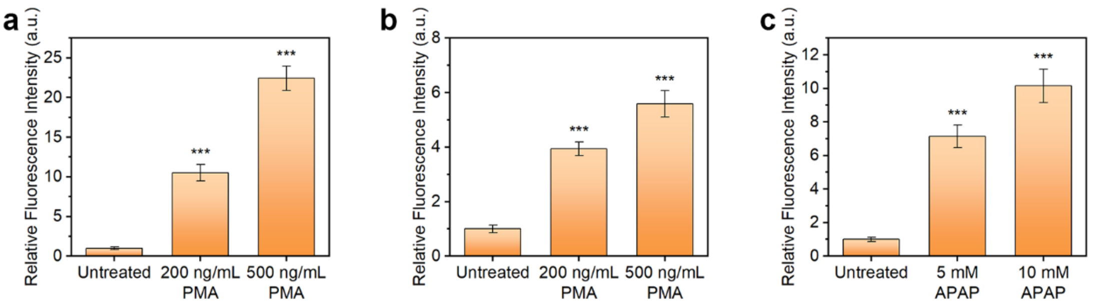

Figure S31. ROS detection in different dosing model groups. The cells in different groups were incubated with 10 μM H_2DCFDA for 30 min at 37 °C, respectively. (a) RAW264.7 cells with untreated, 200 ng/mL PMA or 500 ng/mL PMA for 4 h at 37 °C; (b) HeLa cells with untreated, 200 ng/mL PMA or 500 ng/mL PMA for 4 h at 37 °C; (c) HepG2 cells with untreated, 5 mM APAP or 10 mM APAP for 24 h at 37 °C. Data represent mean ± SD (n = 5), *P<0.05, **P<0.01, ***P<0.001 for treatment compared to the control group using one-way ANOVA with multiple comparisons test.

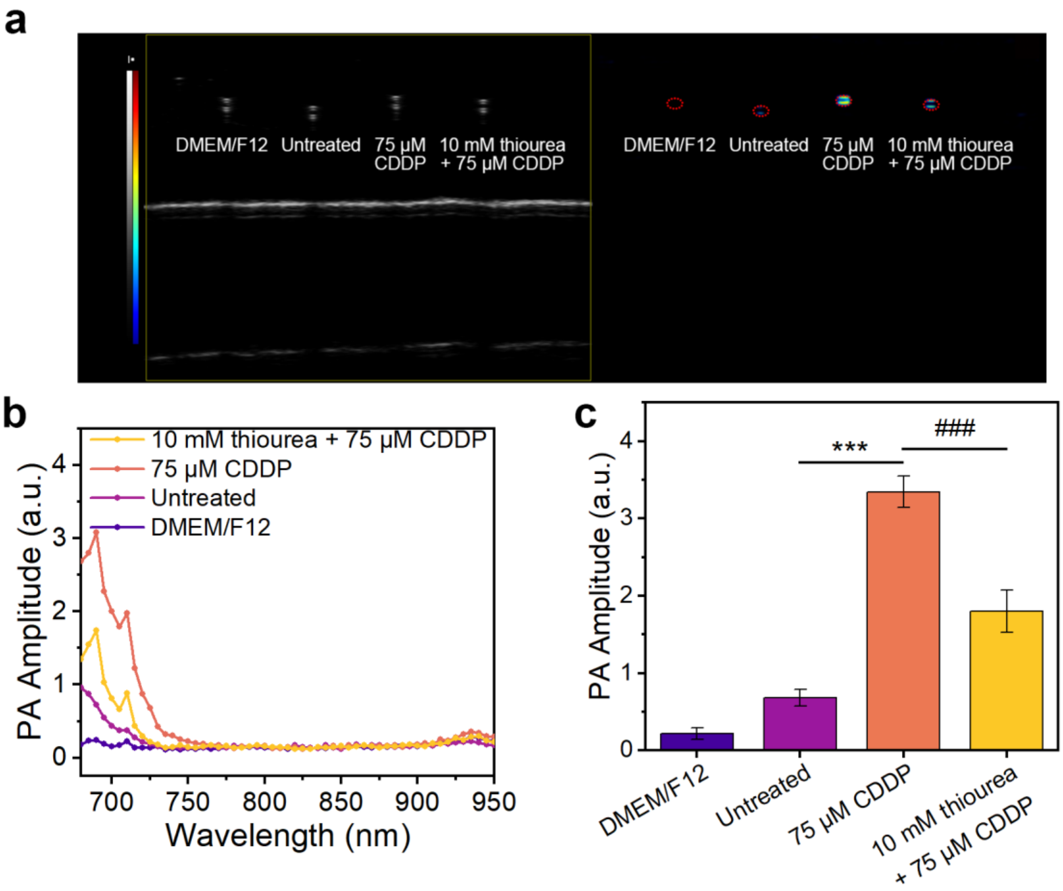

Figure S32. (a) PA imaging of HK-2 cell pellets with untreated, 75 μM CDDP or pre-treated with 10 mM thiourea for 2 h before 75 μM CDDP for 24 h (DMEM/F12 served as control), then incubated with 10 μM CDIA at 37 °C for 30 min. (b) The PA spectra of corresponding cell pellets in (a). (c) The PA amplitude plots of HK-2 cells for different groups. Data represent mean ± SD (n = 5), $^{*}P < 0.05$, $^{**}P < 0.01$, $^{***}P < 0.001$, $^{\#}P < 0.05$, $^{\#\#}P < 0.01$, $^{\#\#\#}P < 0.001$; one-way ANOVA with multiple comparisons test.

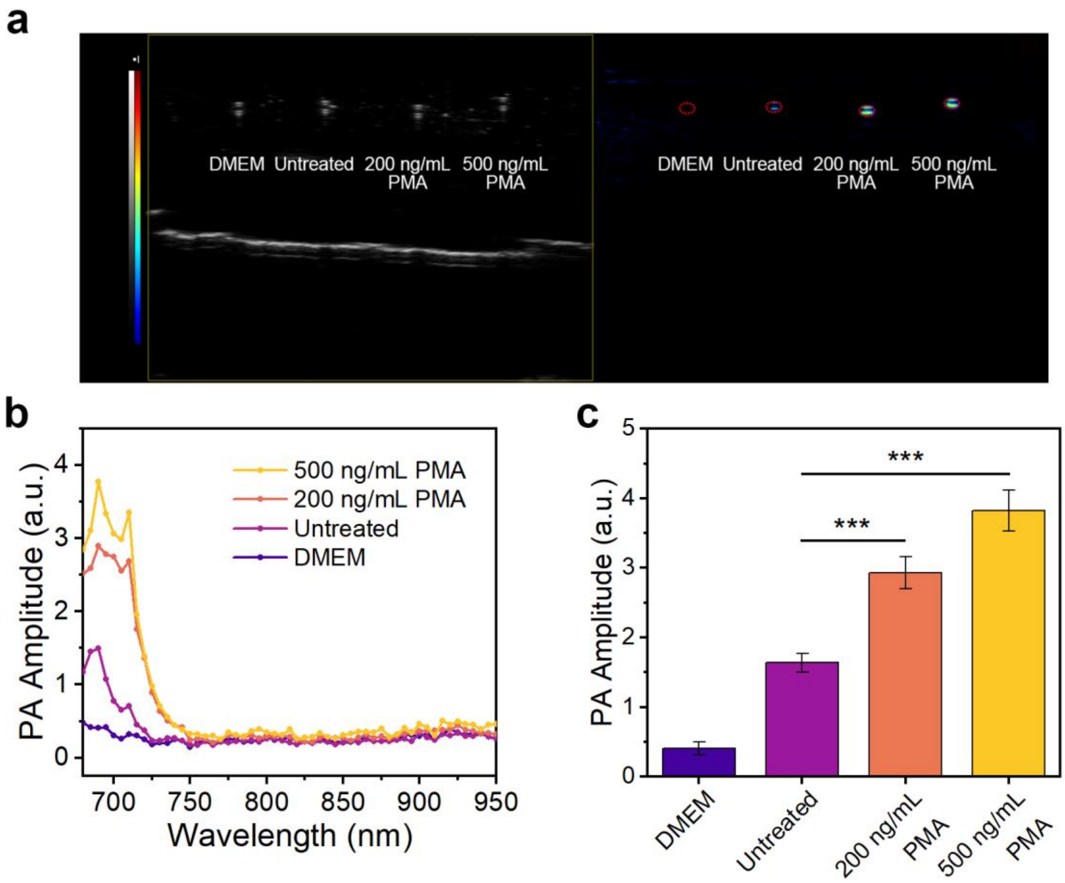

Figure S33. (a) PA imaging of RAW264.7 cell pellets with untreated, 200 ng/mL PMA or 500 ng/mL PMA for 4 h (DMEM served as control), then incubated with 10 μM CDIA at 37 °C for 30 min. (b) The PA spectra of corresponding cell pellets in (a). (c) The PA amplitude plots of RAW264.7 cells for different groups. Data represent mean ± SD (n = 5), **P*<0.05, ***P*<0.01, ****P*<0.001 for treatment compared to the control group using one-way ANOVA with multiple comparisons test.

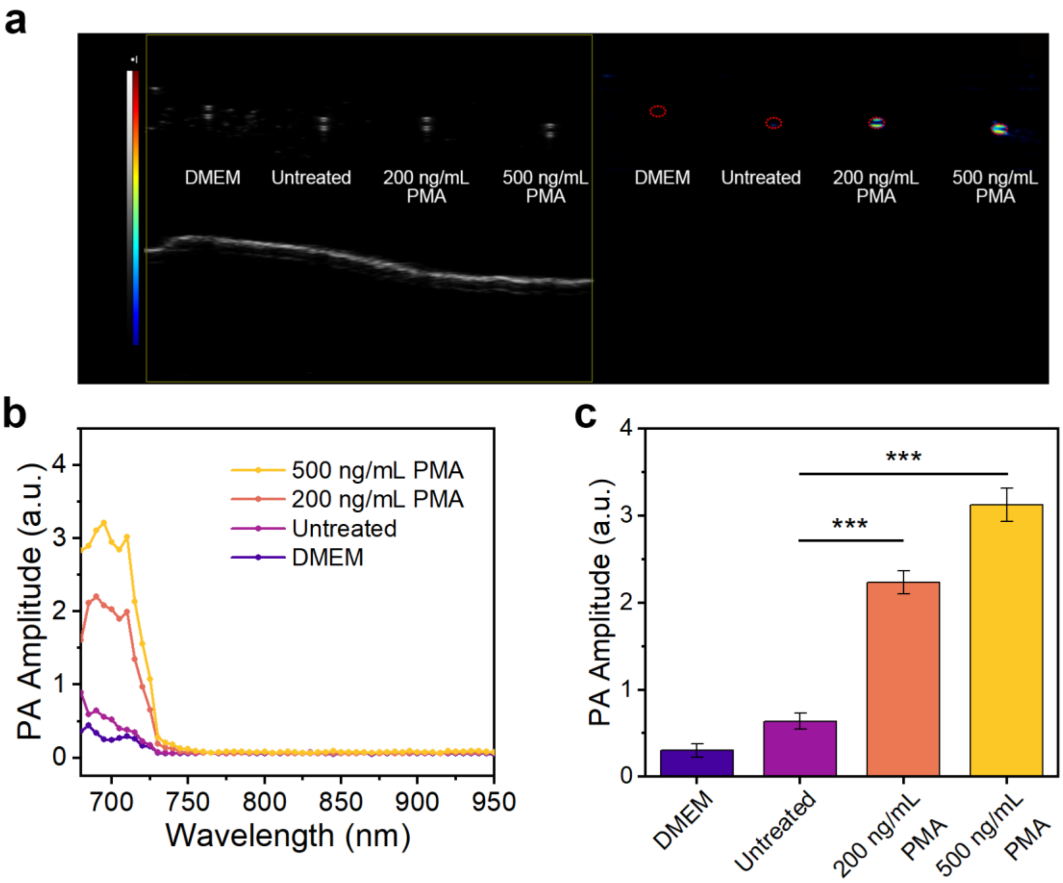

Figure S34. (a) PA imaging of HeLa cell pellets with untreated, 200 ng/mL PMA or 500 ng/mL PMA for 4 h (DMEM served as control), then incubated with 10 μM CDIA at 37 °C for 30 min. (b) The PA spectra of corresponding cell pellets in (a). (c) The PA amplitude plots of HeLa cells in different groups. Data represent mean ± SD (n = 5), **P*<0.05, ***P*<0.01, ****P*<0.001 for treatment compared to the control group using one-way ANOVA with multiple comparisons test.

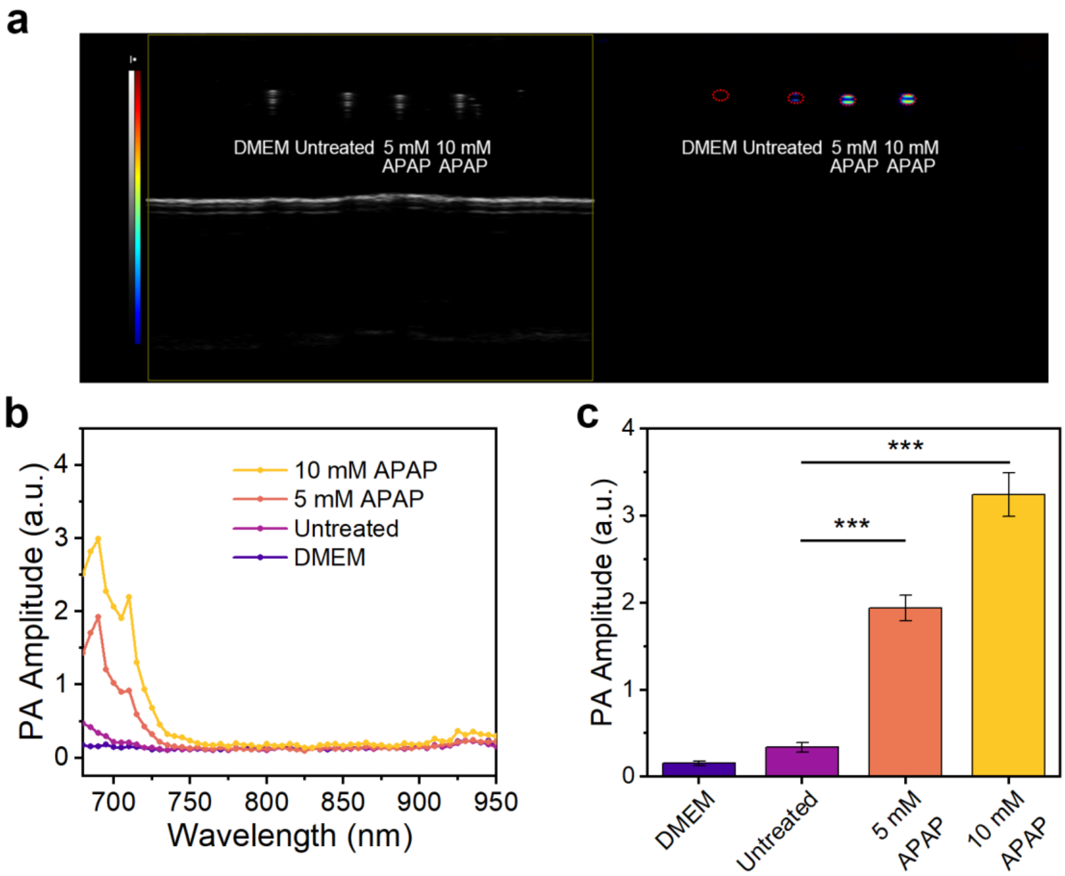

Figure S35. (a) PA imaging of HepG2 cell pellets with untreated, 5 mM APAP or 10 mM APAP for 24 h (DMEM served as control), then incubated with 10 μM CDIA at 37 °C for 30 min. (b) The PA spectra of corresponding cell pellets in (a). (c) The PA amplitude plots of HepG2 cells for different groups. Data represent mean ± SD (n = 5), **P*<0.05, ***P*<0.01, ****P*<0.001 for treatment compared to the control group using one-way ANOVA with multiple comparisons test.

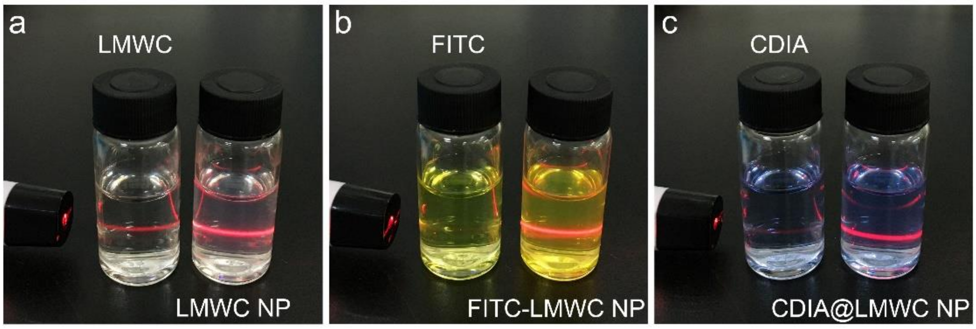

Figure S36. White light images of (a) LMWC NP (20 μg/mL), (b) FITC-LMWC NP (20 μg/mL) and (c) CDIA@LMWC NP (20 μg/mL), respectively. Chitosan particles showed the unique colloid Tyndall phenomenon.

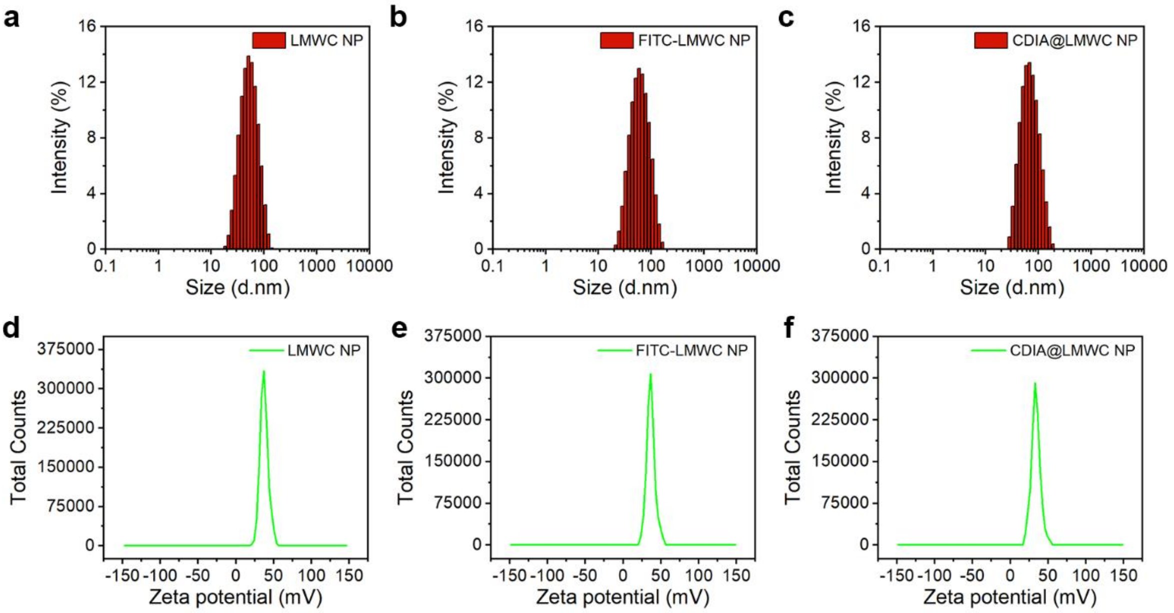

Figure S37. Size distribution of (a) LMWC NP, (b) FITC-LMWC NP, (c) CDIA@LMWC NP, and zeta potential of (d) LMWC NP, (e) FITC-LMWC NP, (f) CDIA@LMWC NP, respectively.

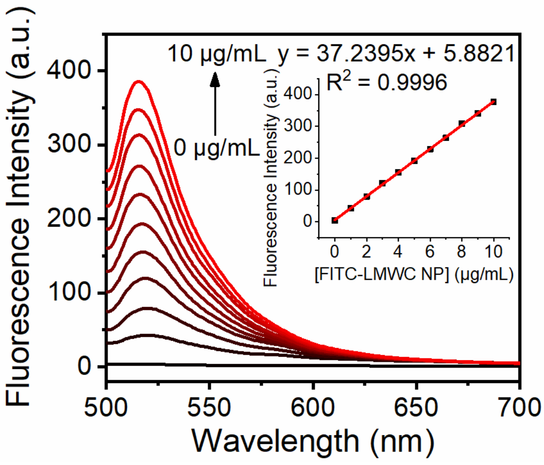

Figure S38. Fluorescence emission spectra of FITC-LMWC NP with different concentrations (λ_{ex} = 490 nm). Inset: The standard curve of FITC-LMWC NP determined by measuring the fluorescence intensity at $\lambda_{ex}/\lambda_{em}$ = 490/518 nm.

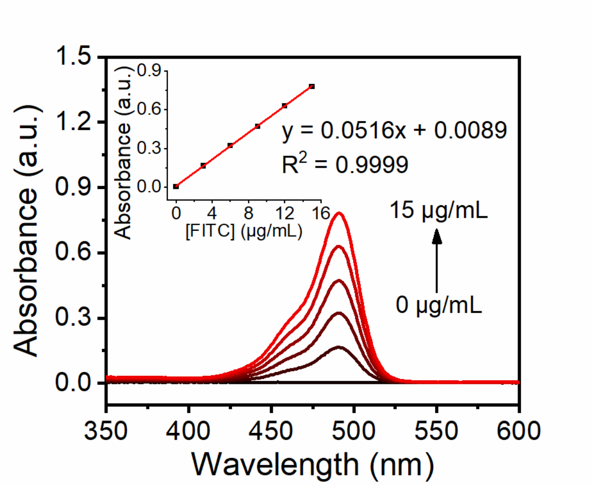

Figure S39. UV-vis absorption spectra of FITC with different concentrations. Inset: The standard curve of FITC determined by measuring the absorbance at 490 nm.

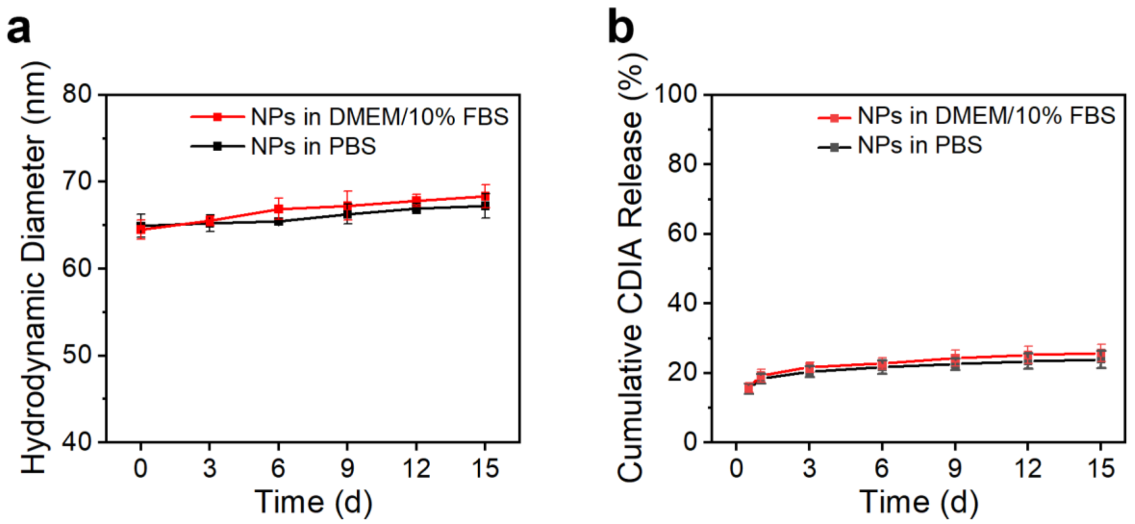

Figure S40. Long-term-stability study of CDIA@LMWC NP in PBS buffer (10 mM, pH 7.4) and DMEM with 10% FBS at 4 °C. (a) Hydrodynamic diameter recording of CDIA@LMWC NP. (b) In vitro release profile of CDIA from CDIA@LMWC NP. Data represent mean ± SD (n = 5).

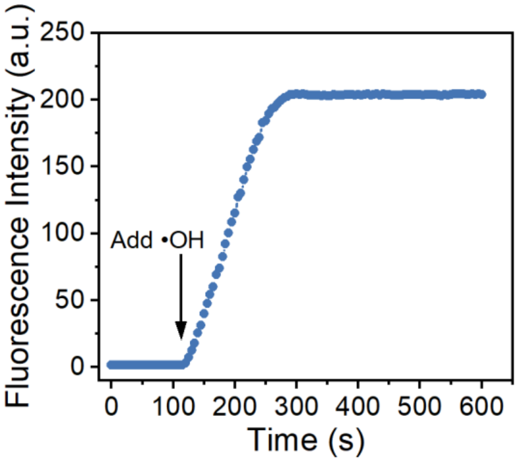

Figure S41. Time-dependent fluorescence changes of 10 μM CDIA with 20 μg/mL LMWC NP toward 100 μM •OH in PBS buffer (10 mM, pH 7.4) at 37 °C, $\lambda_{ex/em}$ = 650/720 nm.

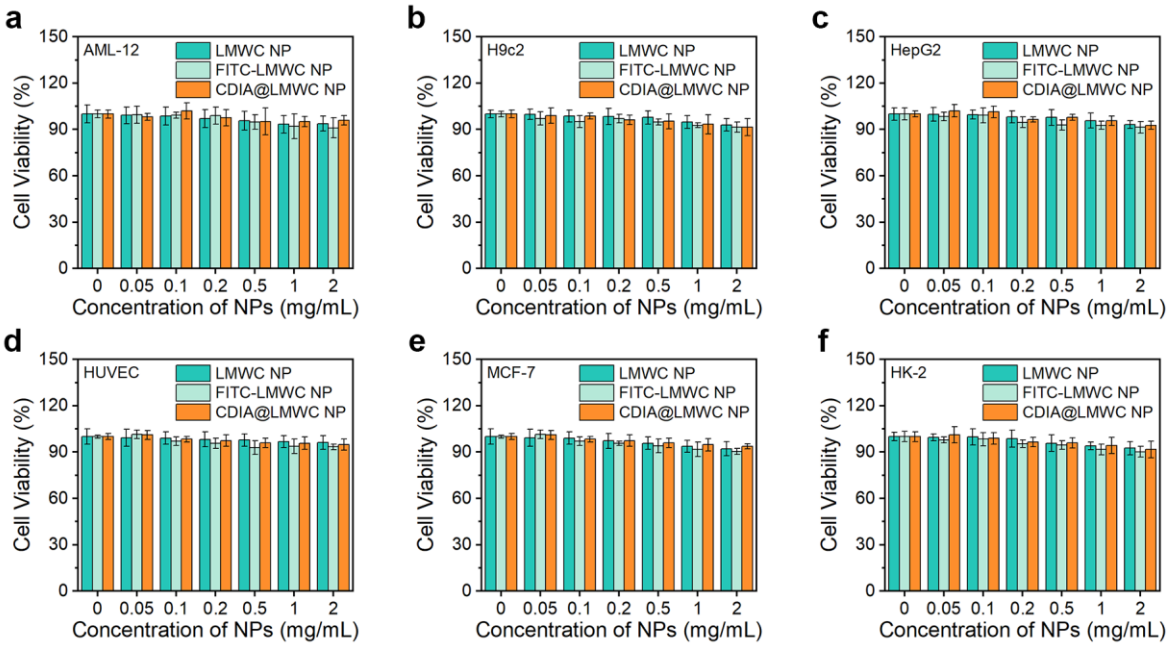

Figure S42. Cell viability assay of AML-12, H9c2, HepG2, HUVEC, MCF-7 and HK-2 cells after being incubated with different concentrations of nanoparticles as indicated at 37 °C for 24 h. Data represent mean ± SD (n = 5).

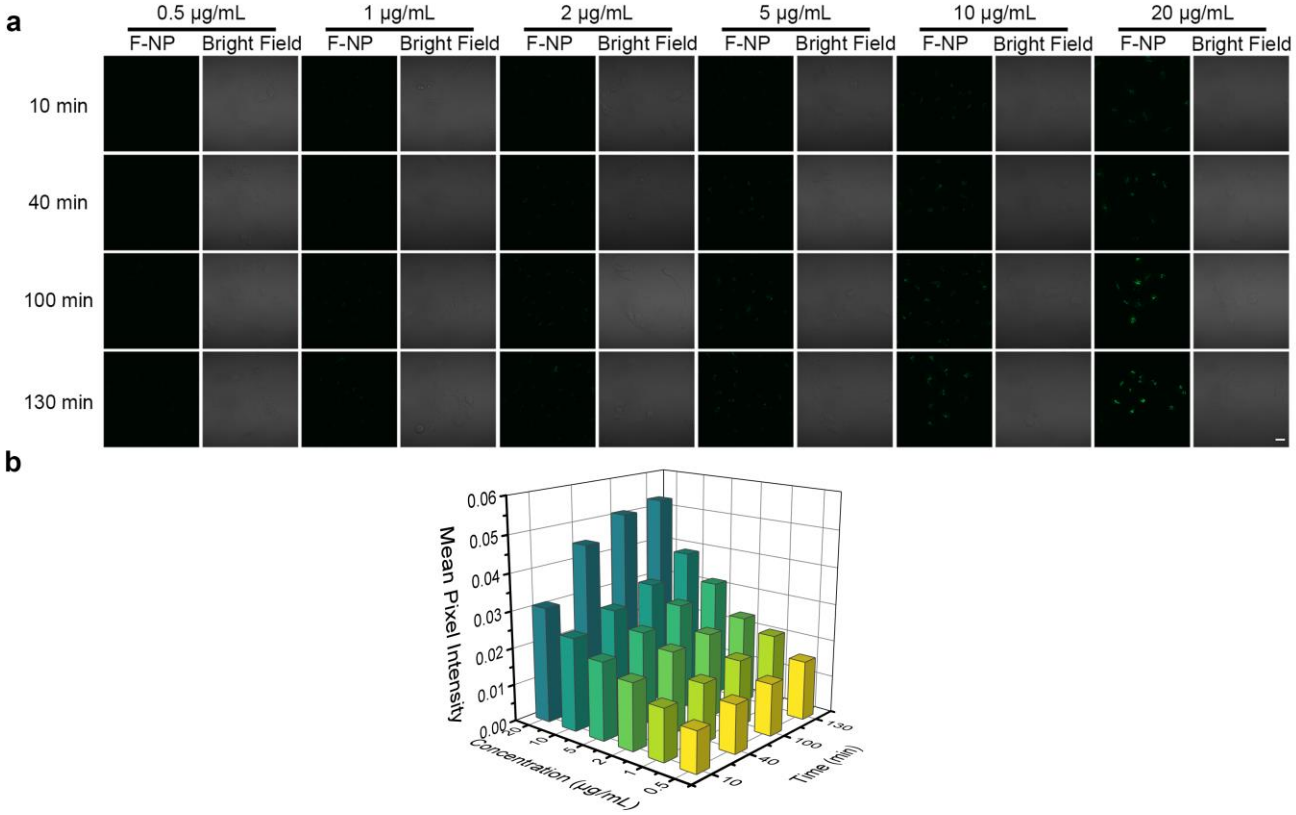

Figure S43. (a) Confocal fluorescence imaging of HK-2 cells incubated with different concentrations of FITC-LMWC NP for different time points at 37 °C. Scale bar: 20 μm. (b) Mean pixel intensity plots of different time points when HK-2 cells incubated with different concentrations of FITC-LMWC NP, respectively.

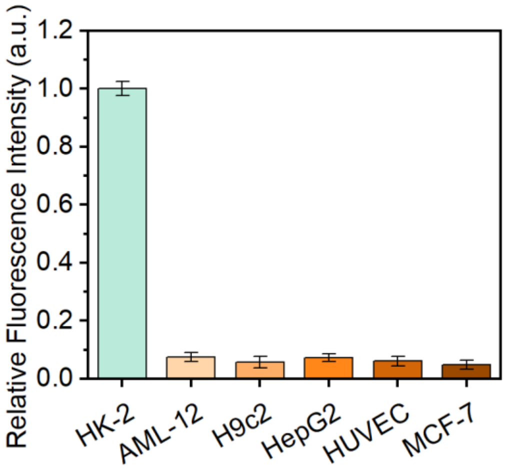

Figure S44. Fluorescence intensity plots of different cells incubated with 20 μg/mL FITC-LMWC NP at 37 °C for 2 h, respectively. Data represent mean ± SD (n = 5).

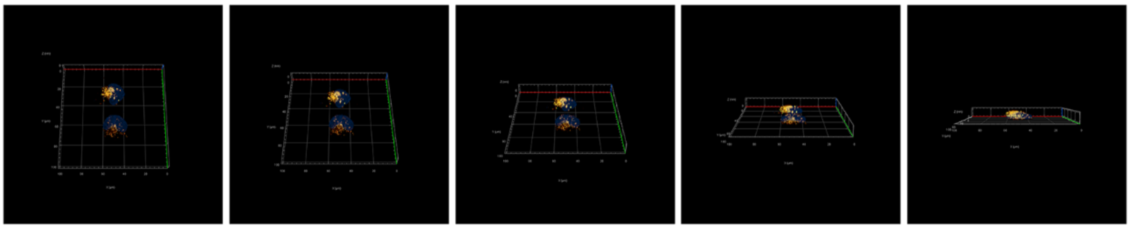

Figure S45. Confocal fluorescence imaging of live HK-2 cells co-stained with 20 μg/mL FITC-LMWC NP, 100 nM Lyso Tracker Red and 10 μg/mL Hoechst 33342 at different sections through the z-axis.

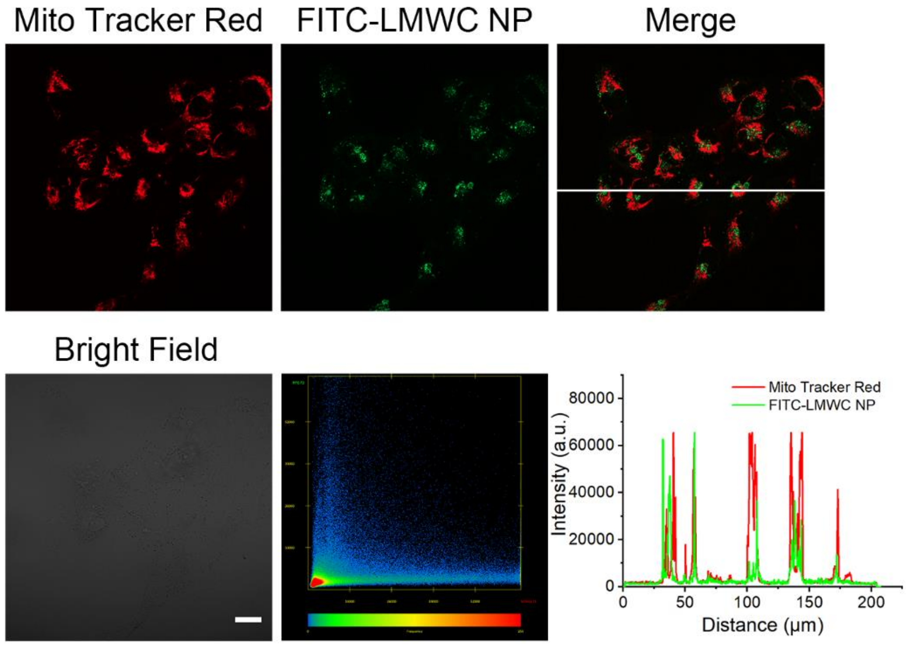

Figure S46. Confocal fluorescence images of HK-2 cells co-stained with FITC-LMWC NP and Mito Tracker Red (Pearson's correlation R_r = 0.1861). Scale bar: 20 μm.

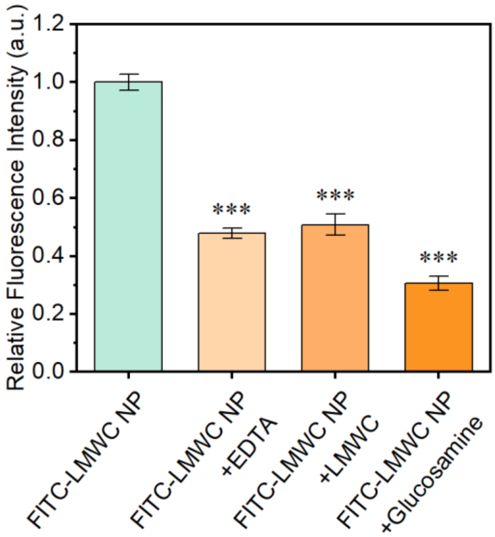

Figure S47. Fluorescence intensity plots of HK-2 cells incubated with 20 μg/mL FITC-LMWC NP and different inhibitors (1 μM EDTA, 50 μg/mL LMWC, or 50 μg/mL glucosamine) at 37 °C for 2 h. Data represent mean ± SD (n = 5), **P*<0.05, ***P*<0.01, ****P*<0.001 for treatment compared to the control group using one-way ANOVA with multiple comparisons test.

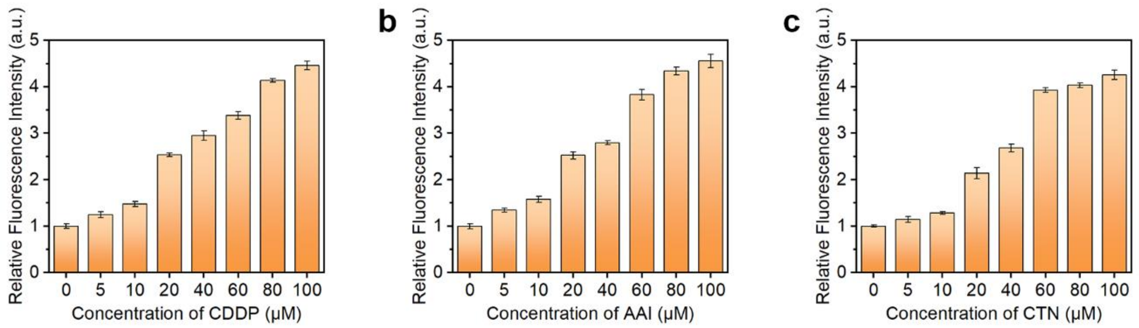

Figure S48. ROS detection for HK-2 cells incubated with different concentrations of (a) CDDP, (b) AAI and (c) CTN for 24 h, respectively, and then incubated with 10 μM H_2DCFDA for 30 min at 37 °C. Data represent means ± SD (n = 5).

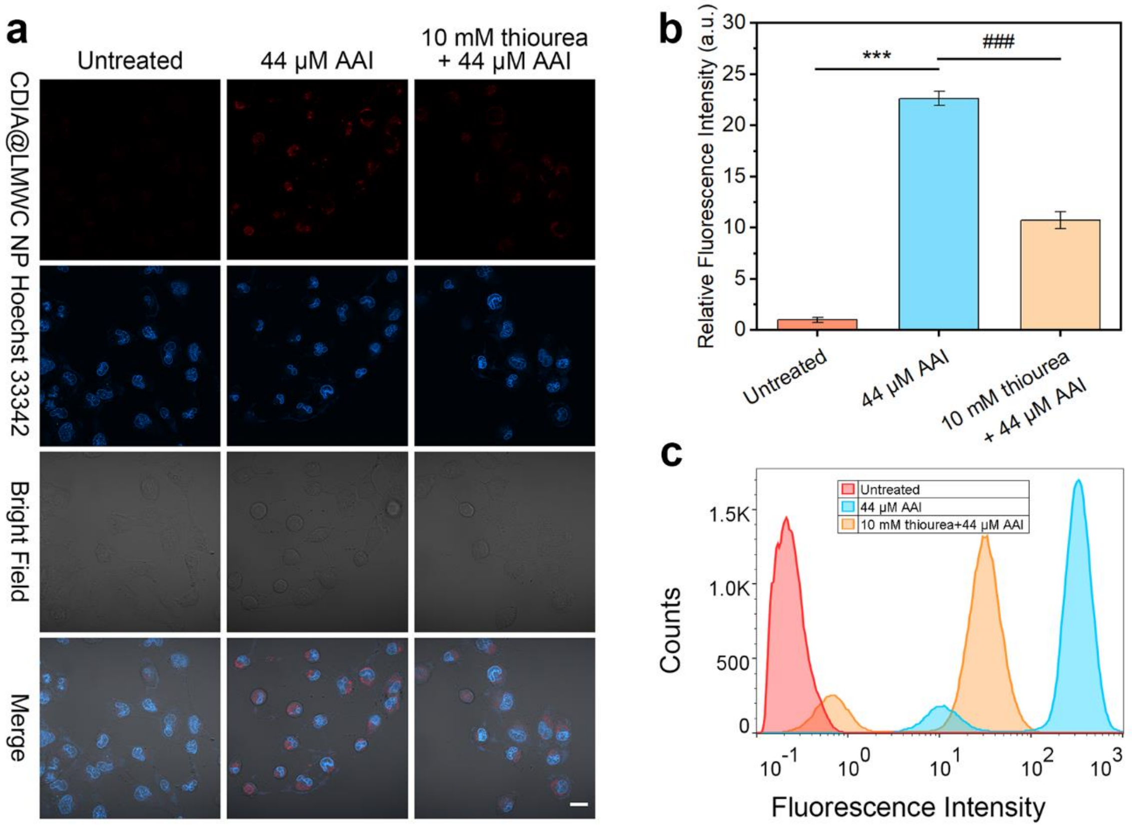

Figure S49. (a) Confocal fluorescence imaging of HK-2 cells with untreated, 44 μM AAI or pre-treated with 10 mM thiourea for 2 h before 44 μM AAI for 24 h, then incubated with 20 μg/mL CDIA@LMWC NP at 37 °C for 2 h. Scale bar: 20 μm. (b) Fluorescence intensity plots of HK-2 cells for different groups. (c) Flow cytometric assays of HK-2 cells for different groups. Data represent mean ± SD (n = 5), $^{*}P < 0.05$, $^{**}P < 0.01$, $^{***}P < 0.001$, $^{\#}P < 0.05$, $^{\#\#}P < 0.01$, $^{\#\#\#}P < 0.001$; one-way ANOVA with multiple comparisons test.

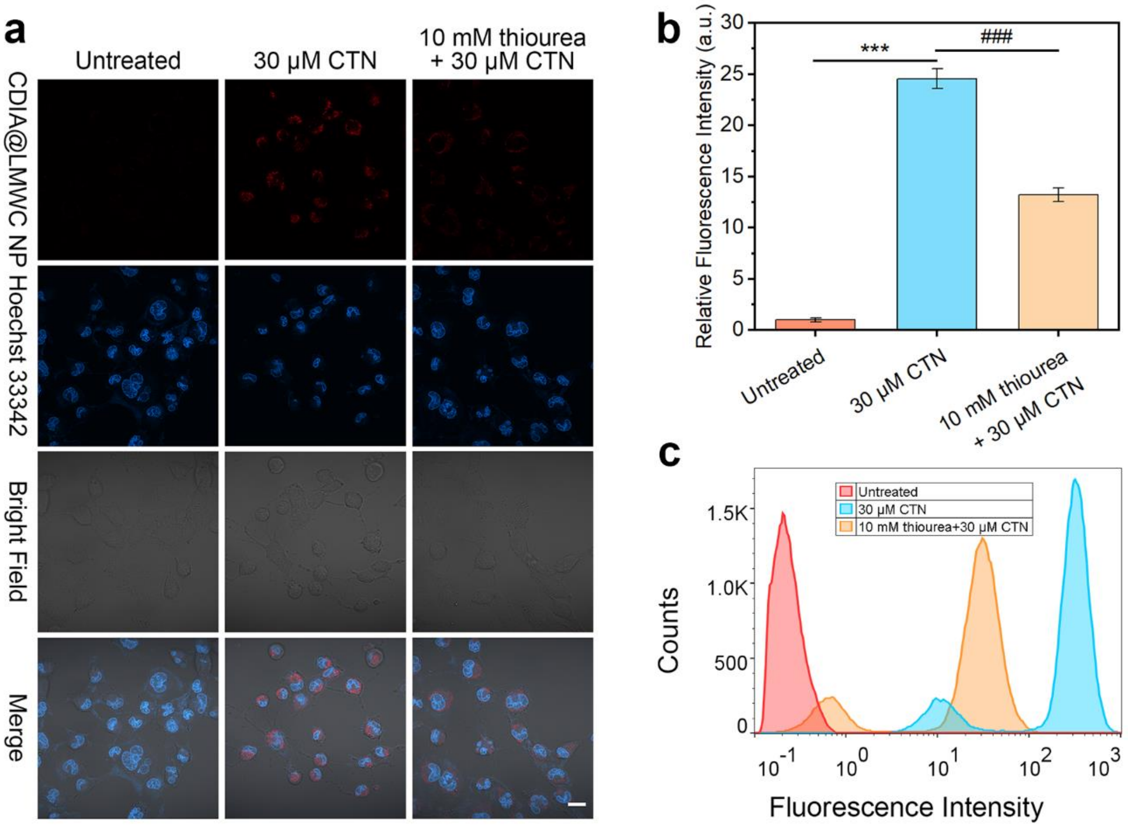

Figure S50. (a) Confocal fluorescence imaging of HK-2 cells with untreated, 30 μM CTN or pre-treated with 10 mM thiourea for 2 h before 30 μM CTN for 24 h, then incubated with 20 μg/mL CDIA@LMWC NP at 37 °C for 2 h. Scale bar: 20 μm. (b) Fluorescence intensity plots of HK-2 cells for different groups. (c) Flow cytometric assays of HK-2 cells for different groups. Data represent mean ± SD (n = 5), $^{*}P < 0.05$, $^{**}P < 0.01$, $^{***}P < 0.001$, $^{\#}P < 0.05$, $^{\#\#}P < 0.01$, $^{\#\#\#}P < 0.001$; one-way ANOVA with multiple comparisons test.

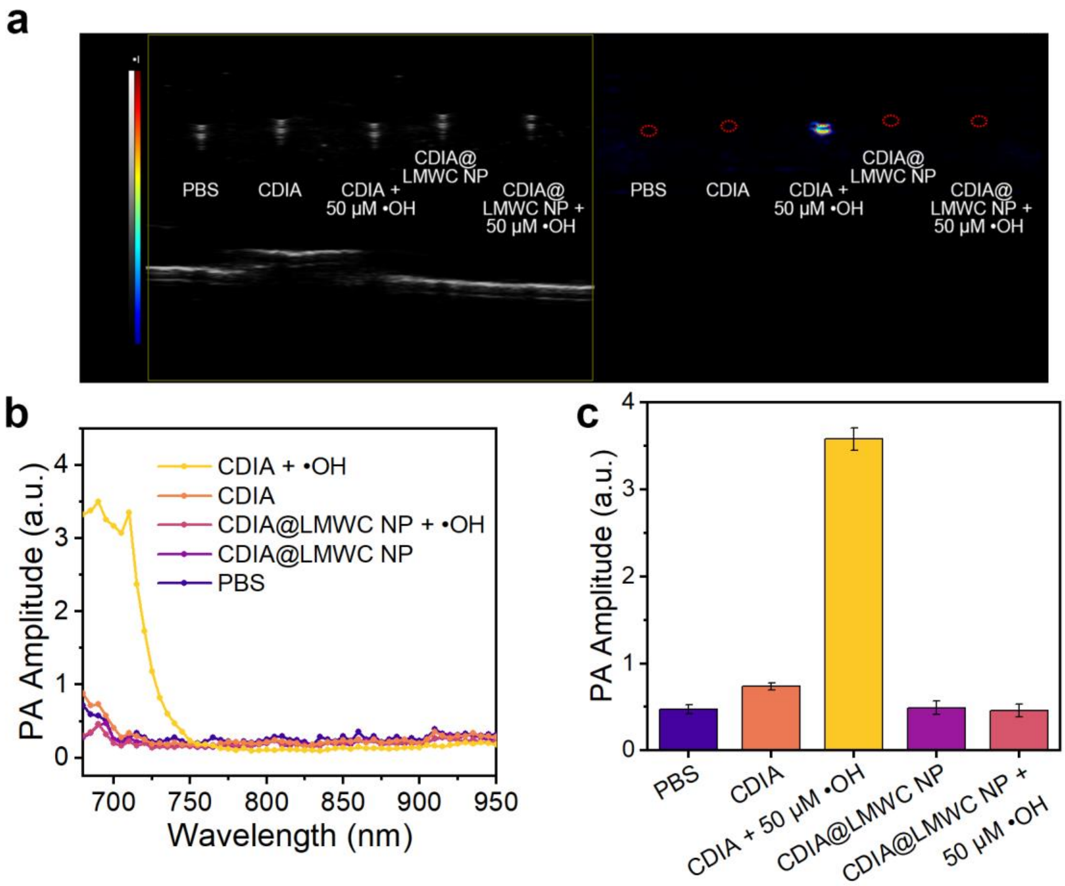

Figure S51. (a) PA imaging of CDIA (10 μM) or CDIA@LMWC NP (20 μg/mL) in the absence or presence of •OH (50 μM) in PBS buffer (10 mM, pH 7.4) at 37 °C for 30 min. (b) The PA spectra of CDIA (10 μM) or CDIA@LMWC NP (20 μg/mL) in the absence or presence of •OH (50 μM) in PBS buffer (10 mM, pH 7.4) at 37 °C for 30 min. (c) The PA amplitude plots of CDIA (10 μM) or CDIA@LMWC NP (20 μg/mL) in the absence or presence of •OH (50 μM) in PBS buffer (10 mM, pH 7.4) at 37 °C for 30 min. Data represent mean ± SD (n = 5).

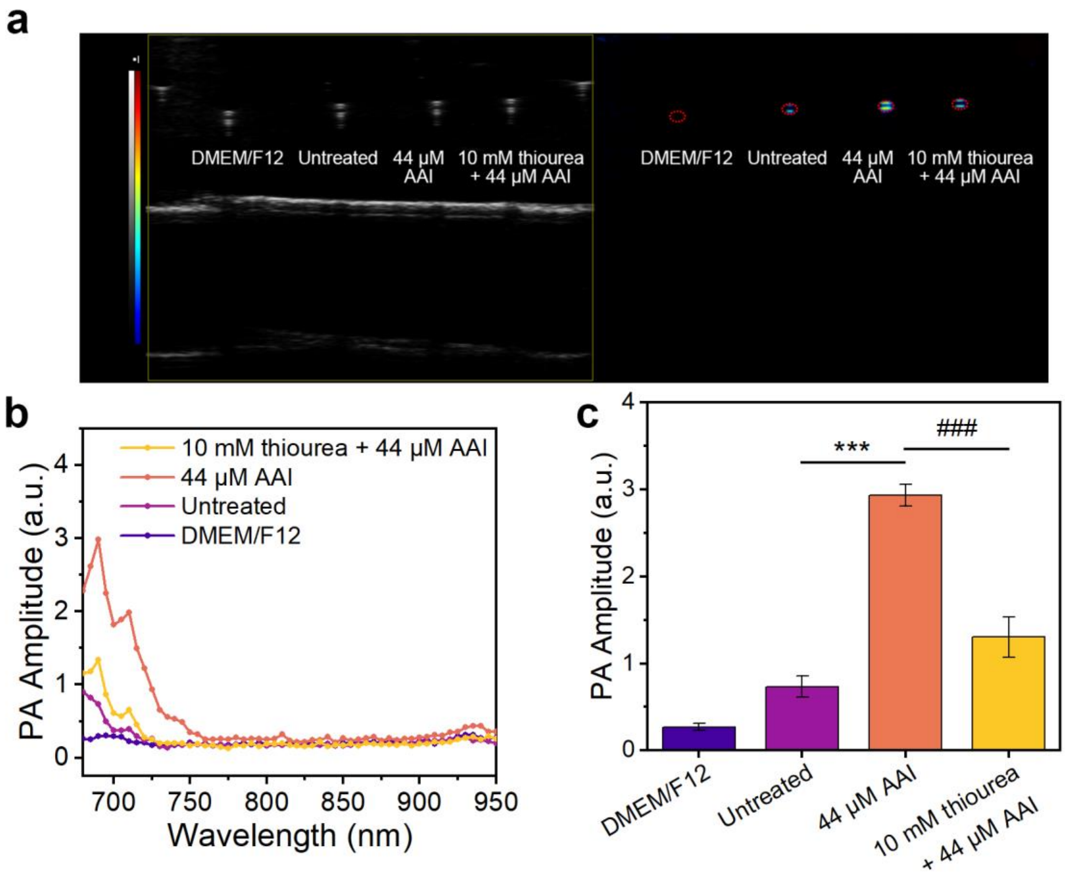

Figure S52. (a) PA imaging of HK-2 cell pellets with untreated, 44 μM AAI or pre-treated with 10 mM thiourea for 2 h before 44 μM AAI for 24 h (DMEM/F12 served as control), then incubated with 20 μg/mL CDIA@LMWC NP at 37 °C for 2 h. (b) The PA spectra of corresponding cell pellets in (a). (c) The PA amplitude plots of HK-2 cells for different groups. Data represent mean ± SD (n = 5), $^{*}P < 0.05$, $^{**}P < 0.01$, $^{***}P < 0.001$, $^{\#}P < 0.05$, $^{\#\#}P < 0.01$, $^{\#\#\#}P < 0.001$; one-way ANOVA with multiple comparisons test.

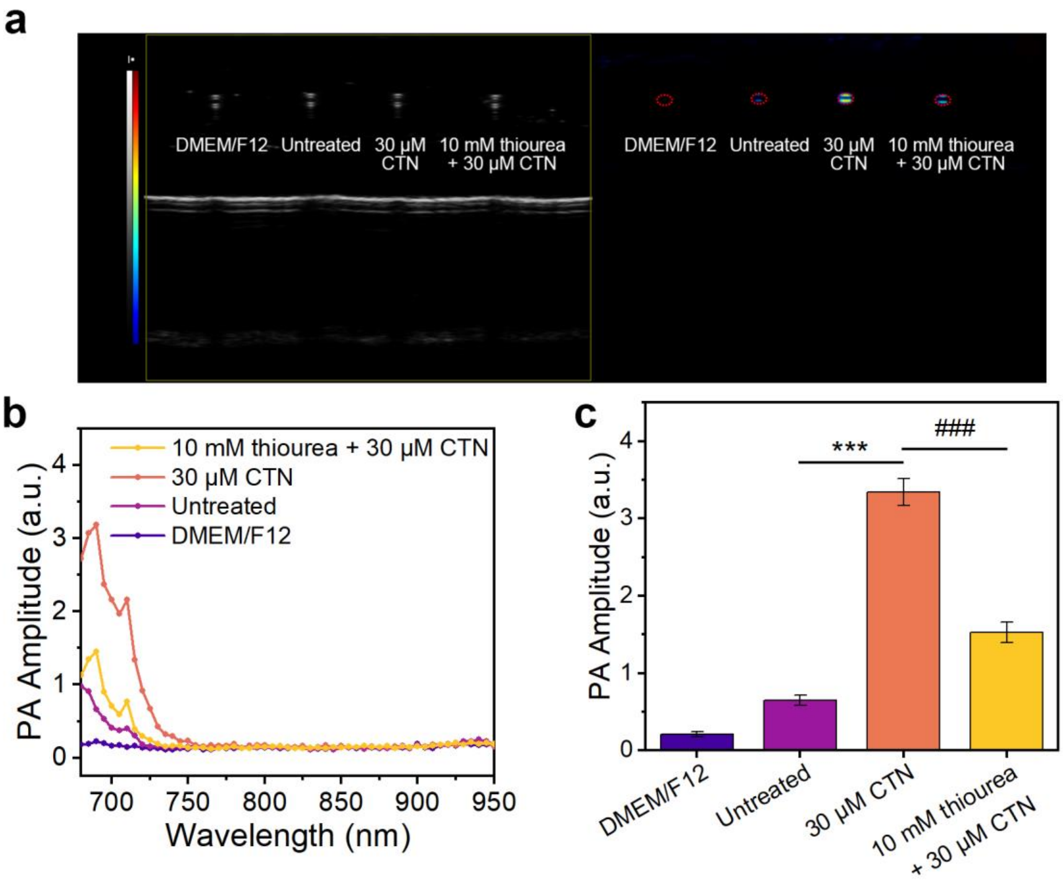

Figure S53. (a) PA imaging of HK-2 cell pellets with untreated, 30 μM CTN or pre-treated with 10 mM thiourea for 2 h before 30 μM CTN for 24 h (DMEM/F12 served as control), then incubated with 20 μg/mL CDIA@LMWC NP at 37 °C for 2 h. (b) The PA spectra of corresponding cell pellets in (a). (c) The PA amplitude plots of HK-2 cells for different groups. Data represent mean ± SD (n = 5), $^{*}P < 0.05$, $^{**}P < 0.01$, $^{***}P < 0.001$, $^{\#}P < 0.05$, $^{\#\#}P < 0.01$, $^{\#\#\#}P < 0.001$; one-way ANOVA with multiple comparisons test.

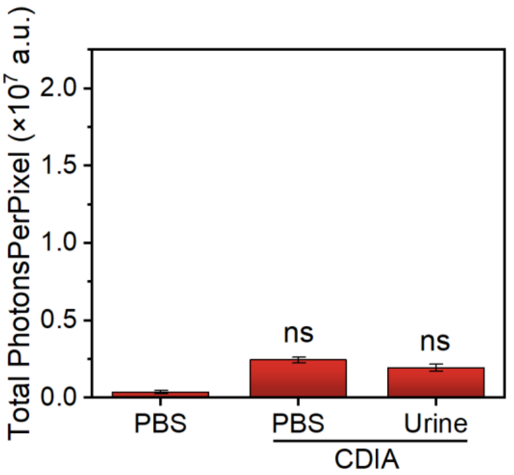

Figure S54. Quantification of NIR fluorescence intensities of CDIA (10 μM) after treatment with PBS and urine, respectively. Data represent mean ± SD (n = 5), *P<0.05, **P<0.01, ***P<0.001 for treatment compared to the control group using one-way ANOVA with multiple comparisons test.

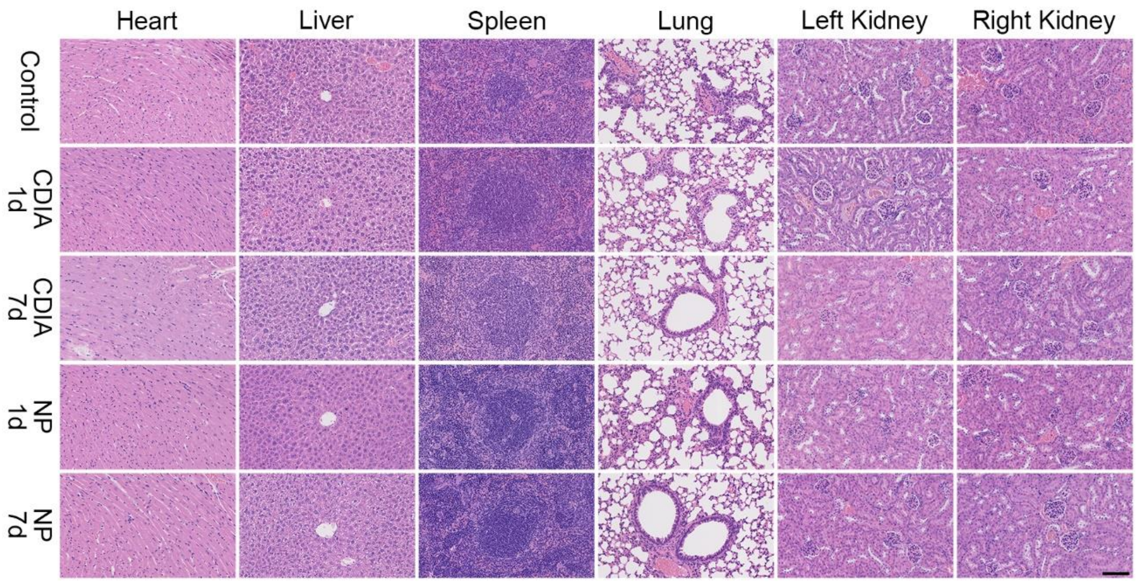

Figure S55. H&E staining images of the main organs from mice after intravenous injected with CDIA (500 μM, 0.2 mL) and CDIA@LMWC NP (20 mg/kg) for 1 d and 7 d, respectively. The images showed that mice have no pathological change in the main organs after injection of CDIA or CDIA@LMWC NP for 1 d and 7 d compared to the control group injected with PBS. Scale bar: 100 μm.

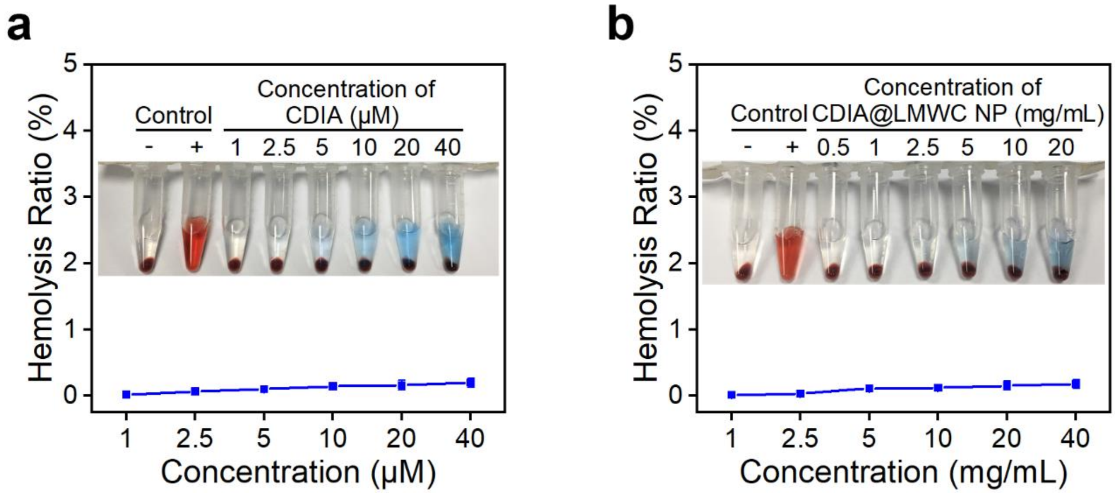

Figure S56. Hemolytic activity of CDIA and CDIA@LMWC NP at different concentrations after incubation with RBCs at 37 ºC for 2 h, respectively.

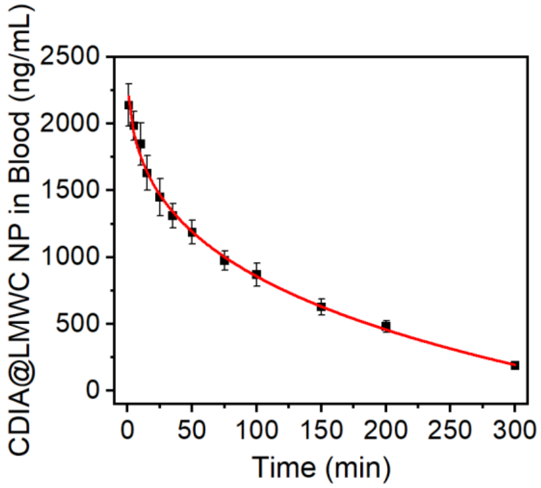

Figure S57. Blood concentration (ng/mL) decay of CDIA@LMWC NP after i.v. injection into living mice. Data represent mean ± SD (n = 5).

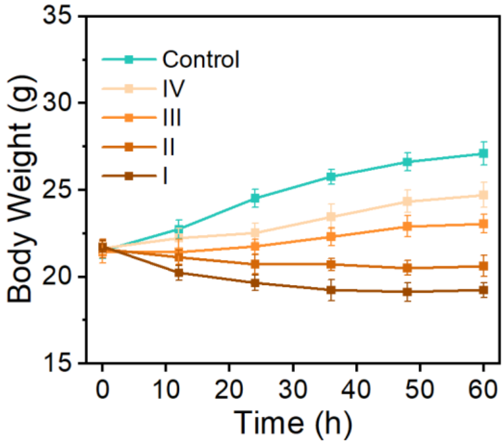

Figure S58. Body weight changes of mice during different treatments. Data represent mean ± SD (n = 5). The mice in 4 groups were treated intraperitoneally with CDDP at the same total dosage of 18 mg/kg body weight. Mice in group I were treated once with CDDP at a dosage of 18 mg/kg; mice in group II were treated twice with CDDP at each dosage of 9 mg/kg; mice in group III were treated thrice with CDDP at each dosage of 6 mg/kg; mice in group IV were treated with CDDP at each dosage of 3 mg/kg for six times. The mice in control group were treated with PBS.

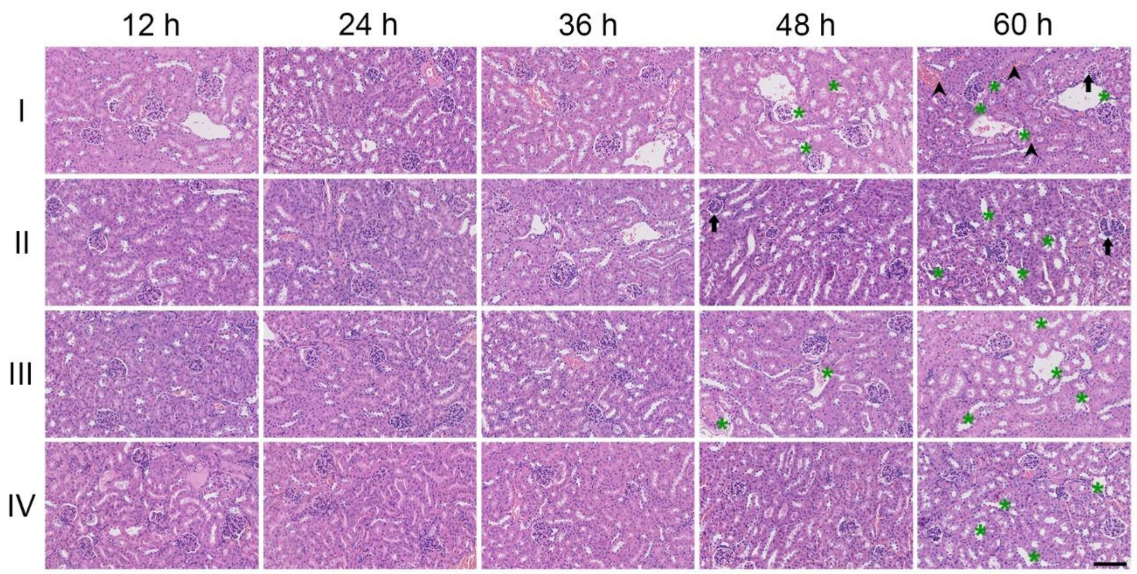

Figure S59. H&E staining images of the kidneys from mice in different groups at different time post-treated with CDDP. Stars indicate for moderate tubules atrophy and dilatation; Arrows indicates for glomeruloatrophy; Arrow head indicates for sloughed cells in the tubular lumen. n = 5 independent animals/group. Scale bar: 100 μm.

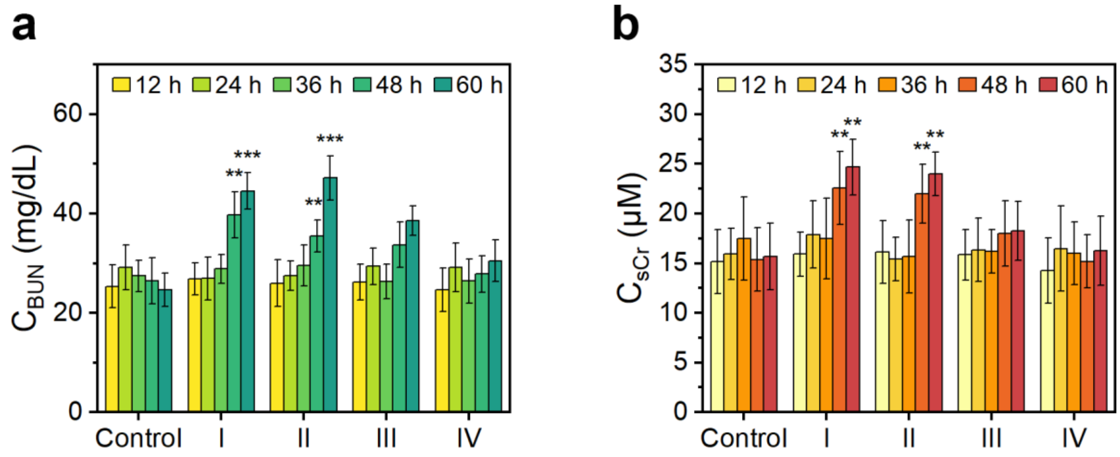

Figure S60. BUN and sCr concentration of the CDDP treated mice in different groups. The graphic showed that only the mice treated with high initial dose of CDDP (group I & group II, 18 mg/kg & 9 mg/kg at first dose, respectively) showed abnormal BUN and sCr increment at 48 h post treatment. Mice treated with multiple injection low dose of CDDP showed similar BUN and sCr level as the control group (treated with PBS). The measurement of BUN and sCr to detect kidney injury was 36 h lag behind the •OH responsive fluorescence-based imaging strategy. Data represent mean ± SD (n = 5), **P*<0.05, ***P*<0.01, ****P*<0.001 for treatment compared to the control group using one-way ANOVA with multiple comparisons test.

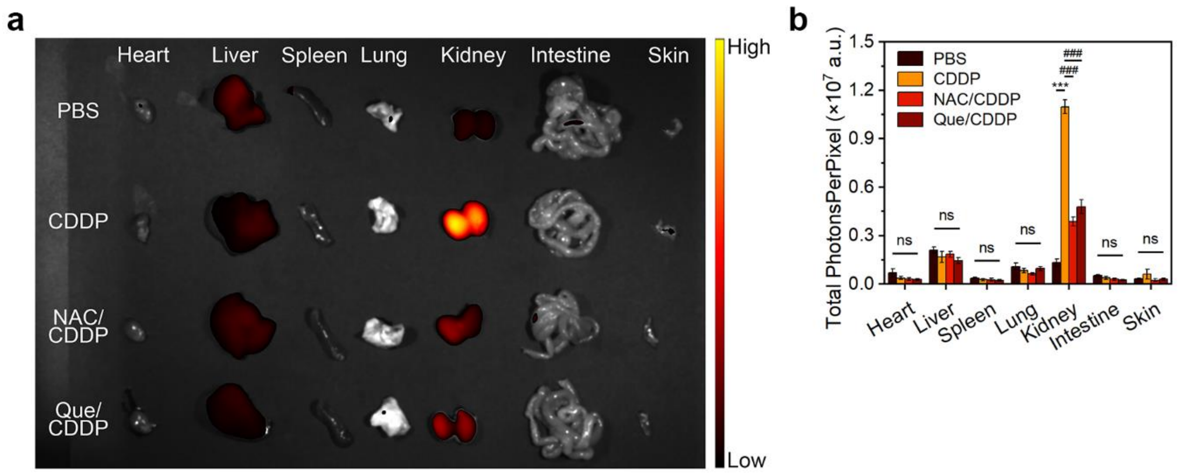

Figure S61. (a) *Ex vivo* NIR images of resected organs from mice after treatment of PBS, or post-treatment of CDDP, or NAC and quercetin prior to CDDP administration, followed by i.v injection of CDIA@LMWC NP (10 mg/kg body weight). (b) Signal quantification of resected organs from mice after treatment of PBS, or post-treatment of CDDP, or NAC and quercetin prior to CDDP administration, followed by i.v. injection of CDIA@LMWC NP (10 mg/kg body weight). Mice were dissected, and major organs were resected after 3 h i.v. injection of CDIA@LMWC NP. Data represent mean ± SD (n = 3) , $^{*}P < 0.05$, $^{**}P < 0.01$, $^{***}P < 0.001$, $^{\#}P < 0.05$, $^{\#\#}P < 0.01$, $^{\#\#\#}P < 0.001$; one-way ANOVA with multiple comparisons test.

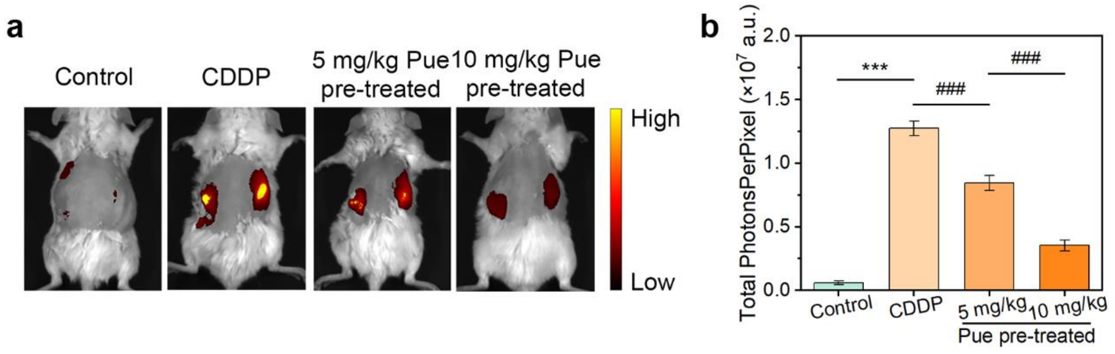

Figure S62. (a) *In vivo* NIR fluorescence images of living mice after injection of CDIA@LMWC NP (10 mg/kg body weight) to different treatment groups. Control: the mice were treated with saline. CDDP: the mice were treated with 18 mg/kg CDDP for 36 h. 5 mg/kg or 10 mg/kg Pue pre-treated: the mice were treated with 5 mg/kg or 10 mg/kg Pue (i.v. injection) 3 days prior to CDDP administration. (b) Fluorescence intensities of mice kidney in (a). Data represent mean ± SD (n = 5), $^{*}P < 0.05$, $^{**}P < 0.01$, $^{***}P < 0.001$, $^{\#}P < 0.05$, $^{\#\#}P < 0.01$, $^{\#\#\#}P < 0.001$; one-way ANOVA with multiple comparisons test.

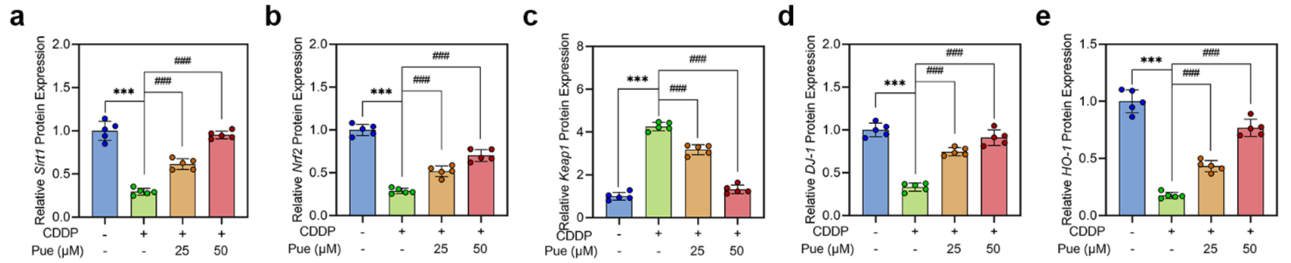

Figure S63. (a) Western blot corresponding densitometric analysis of the (a) Sirt1, (b) Nrf2, (c) Keap1, (d) DJ-1 and (e) HO-1 levels in HK-2 cells after treatments. Data represent mean ± SD (n = 5), $^{*}P < 0.05$, $^{**}P < 0.01$, $^{***}P < 0.001$, $^{\#}P < 0.05$, $^{\#\#}P < 0.01$, $^{\#\#\#}P < 0.001$; one-way ANOVA with multiple comparisons test.

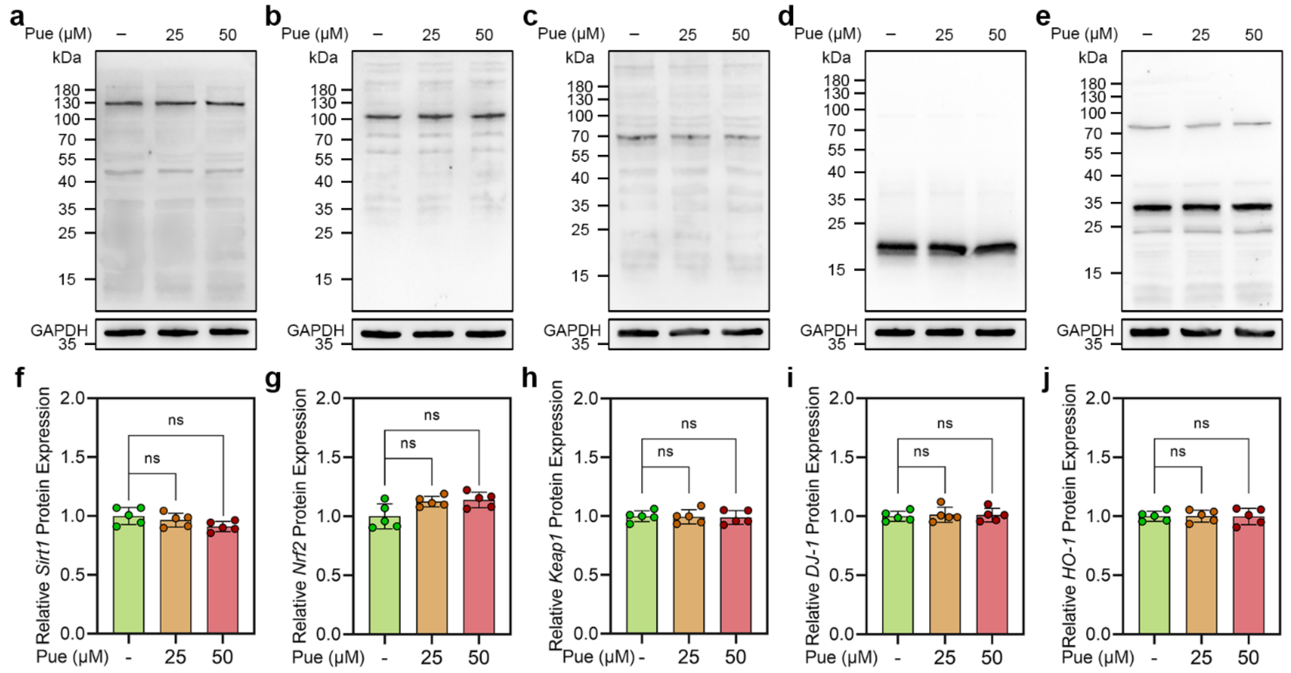

Figure S64. Western blot analysis of the (a) Sirt1, (b) Nrf2, (c) Keap1, (d) DJ-1 and (e) HO-1 levels in HK-2 cells after different concentrations Pue treatments. The corresponding densitometric analysis of the (f) Sirt1, (g) Nrf2, (h) Keap1, (i) DJ-1 and (j) HO-1 levels in HK-2 cells after treatments. Data represent mean ± SD (n = 5), $^{*}P < 0.05$, $^{**}P < 0.01$, $^{***}P < 0.001$, $^{\#}P < 0.05$, $^{\#\#}P < 0.01$, $^{\#\#\#}P < 0.001$; one-way ANOVA with multiple comparisons test.

Table S1. Photophysical properties of CDIA and CyOH.

Probe	λ_{ab} (nm)	λ_{em} (nm)	Stokes shift (nm)	Φ_1/Φ_2
CDIA	650	-	-	0.01
CyOH	690	720	30	0.27

Note: λ_{ab} and λ_{em}: wavelength of maximum absorbance and emission, respectively; Φ_1 and Φ_2 are the fluorescence quantum yields in the absence and presence of •OH, respectively.

Table S2. Analytical performance of reported methods and proposed method for •OH detection.

Limit of detection (M)	Linear range (M)	Detection strategy	References
3×10^{-8}	$0–1.25 \times 10^{-5}$	Rhodamine B labeled upconversion nanoprobe	Chen *et al.*[8]
2.1×10^{-7}	$0–1 \times 10^{-5}$	Carminic acid labeled upconversion nanoprobe	Mei *et al.*[9]
4.48×10^{-6}	$0–2 \times 10^{-4}$	Fluorescent silicon quantum dots–Ce6 complex probe	Zhao *et al.*[10]
7×10^{-8}	$0.001–1.6 \times 10^{-1}$	Coumarin-3-carboxylic acid conjugated carbon nanodots	Zhou *et al.*[11]
1.2×10^{-8}	$0.018–6 \times 10^{-6}$	Terephthalic acid conjugated graphene quantum dots	Hai *et al.*[12]
2×10^{-9}	$0–5 \times 10^{-8}$	Methylene blue coated upconversion nanoparticles	Yu *et al.*[13]
5.30×10^{-9}	$0.001–3 \times 10^{-5}$	An activatable NIR/PA probe	This work

Table S3. Sizes and zeta potentials of different nanoparticles.

Nanoparticles	Hydrodynamic diameter (nm)	Zeta potential (mV)
LMWC NP	58.3 ± 1.9	+ 37.4
FITC-LMWC NP	62.7 ± 1.5	+ 36.8
CDIA@LMWC NP	65.8 ± 2.6	+ 32.9

Table S4. Encapsulation efficiency (EE) and loading content (LC) of CDIA.

Probe	EE (%)	LC (%)
CDIA	83.62 ± 0.17	10.57 ± 0.04

Table S5. Information on the natural compounds in the library used for the high-throughput screening.

Compounds	CAS NO.	Compounds	CAS NO.
**Coumarins**		**Steroids**	
Psoralen	66-97-7	Ursodeoxycholic acid	128-13-2
6,7-Dihydroxycoumarin	305-01-1	Testosterone	58-22-0
Esculin hydrate	531-75-9	**Alkaloids**	
Fraxetin	574-84-5	Berberine	633-65-8
6-Hydroxycoumarin	6093-68-1	Sinomenine	115-53-7
**Sesquiterpenes**		Piperine	94-62-2
Dihydroartemisinin	81496-81-3	Rutecarpine	84-26-4
Curdione	13657-68-6	Capsaicin	404-86-4
Alantolactone	546-43-0	10-Hydroxy camptothecin	19685-09-7
Artesunate	88495-63-0	**Polyphenols**	
**Monoterpenes**		Polydatin	27208-80-6
Catalpol	2415-24-9	Salidroside	10338-51-9
Perillyl alcohol	536-59-4	Resveratrol	501-36-0
**Diterpenes**		Sinapic acid	530-59-6
Andrographolide	5508-58-7	Ferulic acid	1135-24-6
**Triterpenes**		Protocatechuic acid	99-50-3
Oleanolic acid	508-02-1	Guaiacol	90-05-1
Saikosaponin D	20874-52-6	p-Hydroxy-cinnamic acid	7400-08-0
Ginsenoside Re	257-814-6	Chlorogenic acid	327-97-9
**Flavones**		**Quinones**	
(-)-Epicatechin gallate	1257-08-5	Tanshinone IIA	568-72-9
Genistein	446-72-0	5-Hydroxy-2-Methyl-1,4-Naphthoquinon	481-42-5
Myricetin	529-44-2	Rhein	478-43-3
Luteolin	491-70-3	**Others**	
Formononetin	485-72-3	Cantharidin	56-25-7
Isoflavone aglycone	552-66-9	Diosbulbin B	20086-06-0
Naringenin	93602-28-9	Astaxanthin	472-61-7
Curcumin	458-37-7	Limonin	1180-71-8
Chrysin	480-40-0		
Quercetin	117-39-5		
Puerarin	3681-99-0		

Table S6. Detailed sequences of the primers used for qRT-PCR in this research.

Name	Sequence (5’ to 3’)
Human *Sirt1*_forward primer	TGGCAAAGGAGCAGATTAGTAGG
Human *Sirt1*_reverse primer	CTGCCACAAGAACTAGAGGATAAGA
Human *Nrf2*_forward primer	TACTCCCAGGTTGCCCACA
Human *Nrf2*_reverse primer	CATCTACAAACGGGAATGTCTGC
Human *Keap1*_forward primer	TTCGCCTACACGGCCTC
Human *Keap1*_reverse primer	GAAGTTGGCGATGCCGATG
Human *HO-1*_forward primer	TCTTGGCTGGCTTCCTTAC
Human *HO-1*_reverse primer	CATAGGCTCCTTCCTCCTTTC
Human *DJ-1*_forward primer	CCATATGATGTGGTGGTTCTAC
Human *DJ-1*_reverse primer	ACTTCCACAACCTATTTCATGAG
Human *GAPDH*_forward primer	GGTGTGAACCATGAGAAGTATGA
Human *GAPDH*_reverse primer	GAGTCCTTCCACGATACCAAAG

Movie S1. Real-time imaging of fluorescence intensity change of Hoechst 33342 at $\lambda_{ex/em}$ of 405/420–480 nm and CDIA at $\lambda_{ex/em}$ of 640/650–750 nm, respectively.

Movie S2. Real-time monitoring of CDIA@FITC-LMWC NP in CDDP stimulated HK-2 cells at $\lambda_{ex/em}$ of 488/500–650 nm for FITC and $\lambda_{ex/em}$ of 640/650–750 nm for CDIA, respectively.

References

[1] Feng, W.; Zhang, Y.; Li, Z.; Zhai, S.; Lv, W.; Liu, Z. Anal. Chem., 2019, 91, 15757−15762.

[2] Peng, T.; Wong, N. K.; Chen, X.; Chan, Y. K.; Ho, D. H. H.; Sun, Z.; Hu, J. J.; Shen, J.; El-Nezami, H.; Yang, D. J. Am. Chem. Soc., 2014, 136, 11728−11734.

[3] Gao, S.; Hein, S.; Dagnaes-Hansen, F.; Weyer, K.; Yang, C.; Nielsen, R.; Christensen, E. I.; Fenton, R. A.; Kjems, J. Theranostics, 2014, 4, 1039−1051.

[4] Onishi, H.; Machida, Y. Biomaterials, 1999, 20, 175−182.

[5] Huang, L.; Luo, Y.; Sun, X.; Ju, H.; Tian, J.; Yu, B. Y. Biosens. Bioelectron., 2017, 92, 724−732.

[6] Colbay, M.; Yuksel, S.; Uslan, I.; Acarturk, G.; Karaman, O.; Bas, O.; Mollaoglu, H.; Yagmurca, M.; Ozen, O. A. Exp. Toxicol. Pathol., 2010, 62, 81−89.

[7] Li, M.; Tang Z.; Lv S.; Song W.; Hong H.; Jing X.; Zhang Y.; Chen X. Biomaterials, 2014, 35, 3851−3864.

[8] Chen, Z.; Liu, Z.; Li, Z.; Ju, E.; Gao, N.; Zhou, L.; Ren, J.; Qu, X. Biomaterials, 2015, 39, 15–22.

[9] Mei, Q.; Li, Y.; Li, B. N.; Zhang, Y. Biosens. Bioelectron. 2015, 64, 88–93.

[10] Zhao, Q.; Zhang, R.; Ye, D.; Zhang, S.; Chen, H.; Kong, J. ACS Appl. Mater. Interfaces, 2017, 9, 2052–2058.

[11] Zhou, D.; Huang, H.; Wang, Y.; Wang, Y.; Hu, Z.; Li, X. J. Mater. Chem. B, 2019, 7, 3737–3744.

[12] Hai, X.; Guo, Z.; Lin, X.; Chen, X.; Wang, J. ACS Appl. Mater. Interfaces, 2018, 10, 5853–5861.

[13] Yu, G.; Feng, N.; Zhao, D.; Wang, H.; Jin, Y.; Liu, D.; Li, Z.; Yang, X.; Ge, K.; Zhang, J. Sci. China: Life Sci., 2021, 64, 434–442.
